# Supplementary material for: Differences in family planning outcomes between military and general populations in Kinshasa, Democratic Republic of the Congo: a cross-sectional analysis
Source: BMJ Open. 2018 Dec 22;8(12):e022295. doi: 10.1136/bmjopen-2018-022295 (PMC6318504; doi:10.1136/bmjopen-2018-022295)
Supplement: Supplementary data [file bmjopen-2018-022295supp002.pdf]

Household Questionnaire

NO	QUESTIONS AND FILTERS	CODING CATEGORIES	SKIP
**IDENTIFICATION**<br>**Please record the following identifying information prior to beginning the interview.**			
A	**Your name: Is this your name?**<br>[ODK will display the name associated with the phone's serial number.]<br>*Check the button next to the name if that is your name and select 'yes' here. Do not check the button if that is not your name and select 'no' here (long press to remove response next to the name if needed).*	Yes ......1<br>No 0	
	**Enter your name below.**<br>*Please record your name*	Interviewer's Name	
B	**Current date and time.**<br>[ODK will display on screen]<br>**Is this date and time correct?**	Yes ......1<br>No 0	Skip to D if Yes
C	**Record the correct date and time**	Date / Month / Day / Year<br>Time / Hour / Minutes / AM/PM	
D1	**Province**	Kinshasa ...... 1<br>Kongo Central 2	
D2	**City (Kinshasa) / District (Kongo Central)**	ODK will populate the city of Kinshasa and districts in Kongo Central based on the province selected.	
D3	**Commune (Kinshasa) / Aire de santé (Kongo Central)**	ODK will populate a list of appropriate communes in Kinshasa and *aire de santé* in Kongo Central based on the city or district selected.	If Kinshasa was selected in D1, skip to D6
D4	**Quartier (Kinshasa) / Village (Kongo Central)**	ODK will populate a list of appropriate quartiers in Kinshasa and villages in Kongo Central and chiefdoms based on the commune or *aire de santé* selected.	
D5	**Enumeration area**	ODK will populate a list of appropriate enumeration areas based on the neighborhood in Kinshasa or the village in Kongo Central selected.	If Kongo Central selected in D1, skip to E
D6	**Please note the street or avenue number where the household is located**	*THE SUPERVISOR WILL GIVE YOU THE STREET OR AVENUE NAME.*	
E	**Structure number**<br>*Please record the structure number from the household listing form.*	Number	

NO	QUESTIONS AND FILTERS	CODING CATEGORIES	SKIP
F	**Household number**<br>*Please record the household number from the household listing form.*	Number ☐	
	**Check: Have you already sent a form for this structure and household?**<br>*Do not duplicate any form unless you are correcting a mistake in an earlier form.*	Yes ....1<br>No 0	Skip to G if No
	**WARNING: Contact your supervisor before sending this form again.**		
	**CHECK: Why are you resending this form?**<br>*Choose al that apply.*	There are new household members on this form ....1<br>I am correcting a mistake made on a previous form ....2<br>The previous form disappeared from my phone without being sent....3<br>I submitted the previous form and my supervisor told me that is was not received ....4<br>Other reason(s) 5	
G	**Is a member of the household and competent respondent present and available to be interviewed today?**	Yes ....1<br>No 0	Skip to 31 if No
G2	**Did this household participate in a previous PMA2020 survey?**	Yes ....1<br>No ....0<br>Do not know....-88<br>No response ....-99	
**INFORMED CONSENT**<br>**Find a competent member of the household. Read the greeting on the following screen.**			
Hello. My name is ________________________________ and I am working for the School of Public Health of Kinshasa in collaboration with the Ministry of Health. We are conducting a survey in Kinshasa and Kongo Central about various health issues in the Democratic Republic of Congo. We would very much appreciate your participation in this survey. This information will help us inform the government to better plan health services. Whatever information you provide will be kept strictly confidential and will not be shown to anyone other than members of our survey team.<br>Participation in this survey is voluntary, and if we should come to any question you don't want to answer, just let me know and I will go on to the next question; or you can stop the interview at any time. However, we hope that you will participate in this survey since your views are important.<br>I am going to ask you questions about your family and other household members. We would then like to ask a different set of questions to female members of this household who are between the ages of 15 and 49 years.<br>At this time, do you want to ask me anything about the survey?			
H	Provide a paper copy of the Consent Form to the respondent and explain it. Then, ask: **May I begin the interview now?**	Yes ....1<br>No 0	Skip to 32 if No
	**Respondent's signature**<br>*Please ask the respondent to sign or check the box in agreement of their participation.*	Gather signature:<br>Check box: ☐	

NO	QUESTIONS AND FILTERS	CODING CATEGORIES	SKIP
I	**Interviewer's name**<br>*Please record your name as a witness to the consent process. You previously entered "[NAME FROM HQ B]."*		
J	**Respondent's first name.**<br>*Please record the first name of the respondent.*		

SECTION 1 – Household Roster<br>I am now going to ask you a series of questions about each usual member of the household or anyone who slept in the house last night.								
No	1	2	3	4	5	6	7	8
	First name	Sex	Age (years) If less than one year old, record 0.	Marital Status	Relationship to head of household	Family ID	Is this person a usual member of the household or has he/she slept in the house last night?	Eligible female respondent
		Male .... 1<br>Female 2		Married .... 1<br>Living with a partner .. 2<br>Divorced / separated . 3<br>Widow / widower .... 4<br>Never Married .... 5<br>No response........... -99	Head .... 1<br>Wife/Husband .... 2<br>Son/Daughter .... 3<br>Son/Daughter-in-law . 4<br>Grandchild................ 5<br>Parent .... 6<br>Parent in law .... 7<br>Brother/Sister .... 8<br>Other .... 9<br>Don't know .... -88<br>No response.......... -99		Usual member of the household who slept in the house last night .... 1<br>Usual member of the household who did NOT sleep in the house last night .... 2<br>Visitor who slept in the house last night . 3<br>No response .... -99	Yes ..... 1<br>No....... 0<br><br>ODK will determine and display eligibility
1								
2								
3								
4								
5								

After recording information for one household member, the following prompt is asked to activate a looping script to record information for another member

9	Are there any other usual members of your household or persons who slept in the house last night?	Yes .... 1<br>No 0	
	**READ THIS CHECK OUT LOUD: There are [NUMBER OF HOUSEHOLD MEMBERS ENTERED] household members who are named [NAMES OF ENTERED HOUSEHOLD MEMBERS]. Is this a complete list of the household members?**<br><br>*Remember to include all children in the household.*	Yes .... 1<br>No 0	Skip to 10 if Yes

Section 2 – Household Characteristics<br>Now I would like to ask you a few questions about the characteristics of your household.					
**NO**	**QUESTIONS AND FILTERS**	**CODING CATEGORIES**	**Yes**	**No**	**SKIP**
10	**Please tell me about the items you're your household owns. Does your household have:**<br><br>*Read out all types and select all that apply. Scroll to bottom to see all choices.*<br><br>*If an item is reported broken but said to be out of use only temporarily, select the item. Otherwise do not select the item.*	Electricity?	1	0	
		A wall clock?	1	0	
		A radio?	1	0	
		A black/white television?	1	0	
		A color television?	1	0	
		A mobile phone?	1	0	
		A landline telephone?	1	0	
		A refrigerator?	1	0	
		A freezer?	1	0	
		A generator/invertor(s)?	1	0	
		A washing machine?	1	0	
		A computer?	1	0	
		A digital photo camera?	1	0	
		A non digital photo camera?	1	0	
		A video deck?	1	0	
		A DVD/CD?	1	0	
		A sewing machine?	1	0	
		A bed?	1	0	
		A table?	1	0	
		A cabinet/cupboard?	1	0	
		A bicycle?	1	0	
		A motorcycle or motor scooter?	1	0	
		A car or truck?	1	0	
		A boat with a motor?	1	0	
		A boat without a motor?	1	0	
		A gas or electric stove?	1	0	
		A chair(s)?	1	0	
		A lamp(s)?	1	0	
		An oven?	1	0	
		A hoe(s)?	1	0	
		Animal-drawn cart?	1	0	
		A canoe or motorized canoe?	1	0	
		A rental house?	1	0	
		None of the above	-77		
		No response	-99		
11a	**Does this household own any livestock, herds, other farm animals, or poultry?**<br><br>*These livestock can be kept anywhere, not necessarily on the homestead.*	Yes ....1<br>No ....0<br>No response ....-99			Skip to 12a if No or NR

NO	QUESTIONS AND FILTERS	CODING CATEGORIES	SKIP
11b	**How many of the following animals does this household own?**<br>**Zero is a possible answer. Enter -88 for do not know. Enter -99 for no response.**<br>*The household can keep the livestock anywhere but must own the livestock recorded here.*	Cows or bulls ☐<br>Horses, donkeys, or mules ☐<br>Goats ☐<br>Sheep ☐<br>Hogs/pigs ☐<br>Ducks/canes ☐<br>Hens/cocks/other poultry ☐<br>Other ☐	
12a	**Does this household keep any livestock, herds, other farm animals, or poultry ON THE HOMESTEAD, regardless of who owns these livestock?**<br>*Homestead includes the structure and yard that is close to the structure.*	Yes .... 1<br>No .... 0<br>No response .... -99	Skip to 13 if No or NR
12b	**How many of the following animals does this household keep ON THE HOMESTEAD?**<br>**Zero is a possible answer. Enter -88 for do not know. Enter -99 for no response.**<br>*The household does not need to own the livestock recorded here.*	Cows or bulls ☐<br>Horses, donkeys, or mules ☐<br>Goats ☐<br>Sheep ☐<br>Hogs/pigs ☐<br>Ducks/canes ☐<br>Hens/cocks/other poultry ☐<br>Other ☐	
**<u>Section 3 – Household Observation</u>**<br>**Please observe the floors, roof and exterior walls.**			
13	**Main material of the floor**<br>*Observe.*	NATURAL MATERIAL<br>EARTH / SAND .... 11<br>COW DUNG .... 12<br><br>RUDIMENTARY MATERIAL<br>WOODEN BOARDS .... 21<br>PALM / BAMBOO .... 22<br><br>DEVELOPED MATERIAL<br>PARQUET OR POLISHED WOOD .. 31<br>TAPES VINYL / ASPHALT .... 32<br>TILES .... 33<br>CEMENT .... 34<br>CARPET .... 35<br>OTHER .... 96<br>NO RESPONSE .... -99	

NO	QUESTIONS AND FILTERS	CODING CATEGORIES	SKIP
14	**Main material of the roof**<br>*Observe.*	NATURAL MATERIAL<br>NO ROOF .... 11<br>THATCH/PALM ROOF .... 12<br>EARTH MOTTES .... 13<br><br>RUDIMENTARY MATERIAL<br>MATS. .... 21<br>PALM / BAMBOO .... 22<br>WOODEN BOARDS .... 23<br>CARDBOARD .... 24<br><br>DEVELOPED MATERIAL<br>SHEET METAL .... 31<br>WOOD .... 32<br>ZINC / FIBER CEMENT .... 33<br>TILE .... 34<br>CEMENT .... 35<br>SHINGLES .... 36<br>OTHER .... 96<br>NO REPONSE .... -99	
15	**Main material of the exterior walls**<br>*Observe.*	NATURAL MATERIAL<br>NO WALL .... 11<br>BAMBOO / CANE / PALM / TRUNK. 12<br>EARTH .... 13<br><br>RUDIMENTARY MATERIAL<br>BAMBOO WITH MUD .... 21<br>STONES WITH MUD .... 22<br>ADOBE NOT COVERED .... 23<br>PLYWOOD .... 24<br>CARDBOARD .... 25<br>RECOVERED WOOD .... 26<br><br>DEVELOPED MATERIAL<br>CEMENT .... 31<br>STONES WITH LIME / CEMENT ... 321<br>BRICKS .... 332<br>CEMENT BLOCKS .... 343<br>COVERED ADOBE .... 354<br>WOOD BOARD / SHINGLES .... 365<br>OTHER .... 96<br>NO RESPONSE .... -99	
**Section 4 – Water, Sanitation and Hygiene**<br>**Now I would like to ask you a few questions about water, sanitation and hygiene.**			
16	**Do you have a place to wash your hands, or do you have a movable container that is not kept in a fixed location, such as a bowl or kettle, that is commonly used for hand washing?**<br><br>*If the container is always in the same location, then count it as a fixed place*	Yes, fixed place .... 1<br>Yes, movable container .... 2<br>No .... 0<br>Don't know .... -88<br>Refuse to answer .... -99	Skip to 19 if No<br><br>Go to 17a if 16 is 1<br><br>Skip to 17b if 16 is 2
17a	**Can you show it to me?**	Yes .... 1<br>No 0	Skip to 19 if No

NO	QUESTIONS AND FILTERS	CODING CATEGORIES	Yes	No	SKIP
18a	**AT THE PLACE WHERE THE HOUSEHOLD WASHES THEIR HANDS, OBSERVE IF:**	Soap is present<br>Water source is present: stored water<br>Water source is present: running water<br>Hand washing area is near a sanitation facility<br>None of the above	Yes<br>1<br>1<br>1<br>1<br>-77	No<br>0<br>0<br>0<br>0	
17b	**Can you show me any soap, water, and movable container available in the household used for hand washing?**	Yes .... 1<br>No 0			
18b	**ANYWHERE WITHIN THE HOUSEHOLD, OBSERVE IF:**	Soap is present<br>Water source is present: stored water<br>Water source is present: running water<br>Hand washing container Is observed<br>None of the above	Yes<br>1<br>1<br>1<br>-77	No<br>0<br>0<br>0	
19	**Which of the following water sources does your household use on a regular basis for any part of the year for any purpose?**<br>*Read out all types and check all that are used. Scroll to the bottom to see all choices.*	Piped Water<br>Piped into dwelling/indoor<br>Pipe to yard/plot<br>Public tap/standpipe<br>Tube well or borehole<br>Dug Well<br>Protected Well<br>Unprotected Well<br>Water from Spring<br>Protected Spring<br>Unprotected Spring<br>Rainwater<br>Tanker Truck<br>Cart with Small Tank<br>Surface water (River / Dam / Lake / Pond / Stream /Canal / Irrigation Channel)<br>Bottled Water<br>Sachet Water<br>No Response	Yes<br><br>1<br>1<br>1<br>1<br><br>1<br>1<br><br>1<br>1<br>1<br>1<br>1<br>1<br>1<br>1<br>-99	No<br><br>0<br>0<br>0<br>0<br><br>0<br>0<br><br>0<br>0<br>0<br>0<br>0<br>0<br>0<br>0	If only one source is select ed, skip to HQ22
20	**What is the main source of drinking water for members of your household?**<br>**Selections from HQ19: [ODK will list water sources selected for HQ19]**<br>*Read out HQ19 selections only.*	Piped Water<br>Piped into dwelling/indoor .... 1<br>Pipe to yard/plot .... 2<br>Public tap/standpipe .... 3<br>Tube well or borehole .... 4<br>Dug Well<br>Protected Well .... 5<br>Unprotected Well .... 6<br>Water from Spring<br>Protected Spring .... 7<br>Unprotected Spring .... 8<br>Rainwater .... 9<br>Tanker Truck .... 10<br>Cart with Small Tank .... 11<br>Surface water (River / Dam / Lake / Pond / Stream / Canal / Irrigation Channel) .... 12<br>Bottled Water .... 13<br>Sachet Water .... 14<br>No Response .... -99			

NO	QUESTIONS AND FILTERS	CODING CATEGORIES	SKIP
21	**What is the main source of water used by your household for other purposes such as cooking and hand washing?**<br>**Selections from HQ19: [ODK will list water sources selected for HQ19]**<br>*Read out HQ19 selections only.*	Piped Water<br>Piped into dwelling/indoor ....1<br>Pipe to yard/plot ....2<br>Public tap/standpipe ....3<br>Tube well or borehole ....4<br>Dug Well<br>Protected Well ....5<br>Unprotected Well ....6<br>Water from Spring<br>Protected Spring ....7<br>Unprotected Spring ....8<br>Rainwater.. 9<br>Tanker Truck ....10<br>Cart with Small Tank ....11<br>Surface water<br>(River / Dam / Lake / Pond / Stream / Canal / Irrigation Channel) .... 12<br>Bottled Water ....13<br>Sachet Water ....14<br>No Response ....-99	
	**Questions HQ 22 to HQ 25 will repeat x times, once for each water source selected in HQ 19. These sources include:**<br>**[ODK will display HQ19 selections.]**		
22	**You mentioned that you used [WATER SOURCE]. At any time of the year, does your household use water from this source for:**	Yes / No<br>Drinking .... 1 / 0<br>Cooking .... 1 / 0<br>Livestock .... 1 / 0<br>Gardening / agriculture .... 1 / 0<br>Business venture........................... 1 / 0<br>Washing .... 1 / 0<br>No response......................... -99	
23	**Is [WATER SOURCE] typically available:**<br>*Read all choices out loud.*	All of the year .... 1<br>Some of the year.. 2<br>Small part of the year .... 3<br>No response..-99	
24	**At a time of year when you expect to have water from [WATER SOURCE], is it usually available?**	Yes, always .... 1<br>No, intermittent and predictable .... 2<br>No, intermittent and unpredictable .... 3<br>No response..-99	
25	**How long does it take to go to [WATER SOURCE], get water, and come back?**<br>*Zero is a possible answer*<br>*Enter -88 for do not know*<br>*Enter -99 for no response*<br>*Convert time into minutes.*<br>*Answer includes waiting time in line.*	Minutes:	
26	**Does your household have a garden?**<br>*A garden is a place to grow vegetables.*	Yes .... 1<br>No.. 0<br>No response..-99	

NO	QUESTIONS AND FILTERS	CODING CATEGORIES			SKIP
27	**Do members of your household use any of the following toilet facilities?**<br>*Read out all types and check all that are used. Scroll to the bottom to see all choices.*		Yes	No	
		Flush/pour flush toilets connected to:			
		Piped sewer system	1	0	
		Septic tank	1	0	
		Pit Latrine	1	0	
		Elsewhere	1	0	
		Unknown / Not sure / Don't know	1	0	
		Ventilated improved pit latrine	1	0	
		Pit latrine with slab	1	0	
		Pit latrine without slab	1	0	
		Bucket toilet	1	0	
		Composting toilet	1	0	
		Hanging toilet /Hanging latrine	1	0	
		No facility / bush / field	1	0	
		Other:	1	0	
		No Response	-99		
28	**What is the main toilet facility used by members of your household?**<br>**HQ27: [ODK will display HQ27 selections]**<br>*The main facility must be selected in HQ 27.*	Flush/pour flush toilets connected to:<br>Piped sewer system ..... 1<br>Septic tank ..... 2<br>Pit Latrine ..... 13<br>Elsewhere ..... 3<br>Unknown / Not sure / Don't know ..... 4<br>Ventilated improved pit latrine ..... 5<br>Pit latrine with slab ..... 6<br>Pit latrine without slab/open pit ..... 7<br>Bucket toilet ..... 8<br>Composting toilet ..... 9<br>Hanging toilet /Hanging latrine ..... 10<br>No facility / bush / field ..... 11<br>Other: ..... 12<br>No Response ..... -99			
	**Question HQ 29 will repeat x times, once for each sanitation facility selected in HQ27. These facilities include:**<br>**HQ27: [ODK will display HQ27 selections]**				
29	**How often does your household typically use: [TOILET FACILITY TYPE]?**<br>*Regular practices at the household only.*	Always ..... 1<br>Most of the time ..... 2<br>Occasionally ..... 3<br>Rarely ..... 4<br>No response ..... -99			
29b	**Do you share this toilet facility with other households or the public?**	Not shared ..... 1<br>Shared with less than ten households ..... 2<br>Shared with ten or more households ..... 3<br>Shared with the public. ..... 4<br>No response ..... -99			Skip to HQ30 if not 2

NO	QUESTIONS AND FILTERS	CODING CATEGORIES	SKIP
29c	**Enter the number of households that share this facility (including your own).**<br>**[TOILET FACILITY TYPE]**<br>*Must be between 2 and 9.*<br>*If 10 or greater, swipe back to HQ29b and choose “shared with ten or more households.”*<br>*Enter -99 for no response.*	Number of Households: ☐	
30	**How many people within your household regularly use the bush / field at home or at work?**<br>**There are x people in this household. Enter -88 for do not know, -99 for no response.**	Number of People: ☐	
31	**Ask permission to take a photo of the entrance of the house.**<br>**Did you get consent to take the photo?**	Yes .... 1<br>No 0	Skip L if No
**Thank the respondent for her/his time.**<br>*The respondent is finished, but there is still more for you to complete outside the home.*			
**LOCATION AND QUESTIONNAIRE RESULT**			
K	**Location**<br>**Take a GPS point near the entrance to the household. Record location when the accuracy is smaller than 6m.**	RECORD LOCATION	
	**CHECK 32:** Permission to take photo?		Skip to M if No
L	**Ensure that no people are in the photo**	TAKE PICTURE<br>CHOOSE IMAGE	
M	**How many times have you visited this household?**	1st time .... 1<br>2nd time .... 2<br>3rd time 3	
N	**Questionnaire result**<br>*Record the result of the Household Questionnaire*	Completed .... 1<br>No household member at home or no competent respondent at home at time of visit .... 2<br>Postponed .... 3<br>Refused .... 4<br>Partly completed .... 5<br>Dwelling vacant or address not a dwelling .... 6<br>Dwelling destroyed .... 7<br>Dwelling not found .... 8<br>Entire household absent for extended period of time 9	
