# Supplementary material for: Differences in family planning outcomes between military and general populations in Kinshasa, Democratic Republic of the Congo: a cross-sectional analysis
Source: BMJ Open. 2018 Dec 22;8(12):e022295. doi: 10.1136/bmjopen-2018-022295 (PMC6318504; doi:10.1136/bmjopen-2018-022295)
Supplement: Author's manuscript [file bmjopen-2018-022295.draft_revisions.pdf]

BMJ Open is committed to open peer review. As part of this commitment we make the peer review history of every article we publish publicly available.

When an article is published we post the peer reviewers' comments and the authors' responses online. We also post the versions of the paper that were used during peer review. These are the versions that the peer review comments apply to.

The versions of the paper that follow are the versions that were submitted during the peer review process. They are not the versions of record or the final published versions. They should not be cited or distributed as the published version of this manuscript.

BMJ Open is an open access journal and the full, final, typeset and author-corrected version of record of the manuscript is available on our site with no access controls, subscription charges or pay-per-view fees (http://bmjopen.bmj.com).

If you have any questions on BMJ Open's open peer review process please email info.bmjopen@bmj.com

BMJ Open

Differences in Family Planning Outcomes between Military and General Populations in Kinshasa, Democratic Republic of Congo: A Cross-Sectional Analysis

Journal:	*BMJ Open*
Manuscript ID	bmjopen-2018-022295
Article Type:	Research
Date Submitted by the Author:	09-Feb-2018
Complete List of Authors:	Akilimali, Pierre; Universite de Kinshasa, Kinshasa School of Public Health<br>Anglewicz, Philip; Tulane University School of Public Health and Tropical Medicine, Department of Global Community Health and Behavioral Science;<br>Engale, Henri; Congolese Armed Forces, Medical Division<br>Kurhenga, Gilbert; Congolese Armed Forces, Medical Division<br>Hernandez, Julie; Tulane University, School of Public Health and Tropical Medicine<br>Kayembe, Patrick; University of Kinshasa, Kinshasa School of Public Health<br>Bertrand, Jane; Tulane University
Keywords:	Military, Fertility, Family planning, Contraceptive use, Kinshasa, Democratic Republic of Congo

SCHOLARONE™
Manuscripts

Differences in Family Planning Outcomes between Military and General Populations in Kinshasa, Democratic Republic of Congo: A Cross-Sectional Analysis

For peer review only

Authors

Pierre Akilimali
Kinshasa School of Public Health, University of Kinshasa, BP 11850 Kinshasa 1, Democratic Republic of Congo. Email: pierretulanefp@gmail.com.

Philip Anglewicz (corresponding author)
School of Public Health and Tropical Medicine, Tulane University, 1440 Canal St Suite 2210, New Orleans, LA, USA. Email: panglewi@tulane.edu Phone: (504) 988-5428; Fax: (504) 988-5480.

Henri Nzuka Engale
Medical Division, Congolese Armed Forces, Email: nzukaengale@yahoo.fr

Gilbert Kabanda Kurhenga
Chief, Medical division, Congolese Armed Forces, Email: gilkaku@yahoo.fr

Julie Hernandez
School of Public Health and Tropical Medicine, Tulane University, 1440 Canal St Suite 1900, New Orleans, LA, USA. Email: jhernan7@tulane.edu

Patrick Kayembe
Kinshasa School of Public Health, University of Kinshasa, BP 11850 Kinshasa 1, Democratic Republic of Congo. Email: patkayembe@yahoo.fr

Jane Bertrand
School of Public Health and Tropical Medicine, Tulane University, 1440 Canal St Suite 1900, New Orleans, LA, USA. Email: bertrand@tulane.edu

For peer review only - http://bmjopen.bmj.com/site/about/guidelines.xhtml

Abstract:

Objectives: To examine family planning practices among women in military camps in Kinshasa, Democratic Republic of Congo, and compare with a representative sample of non-military women in Kinshasa.

Participants: Women of reproductive ages, 15 to 49. We compare two populations: women living in military camps and the general (non-military) population in Kinshasa.

Study Design: For sampling, we used a two-stage cluster sampling design, where we first randomly selected enumeration areas (EA), and then randomly selected women within each EA (separately for each of the two populations). Selected women were administered a survey on contraceptive use and family planning. We use bivariate and multivariate analysis to compare these populations for a range of family planning outcomes, including fertility, contraceptive use (overall, modern and traditional), obtaining desired family planning method, whether the last birth was unintended, desire for an additional child, family planning knowledge, and exposure to family planning messages.

Results: We find many strong and highly statistically significant differences between the military and general population of Kinshasa. Although they do not have more children, the military are less likely to be using contraception and less knowledgeable about many family planning methods, while at the same time they are more likely to want to limit their births and less likely to have obtained their preferred family planning method.

Conclusions: The military in Kinshasa appears to be an important and underserved population with regards to family planning. Our results suggest that women in military camps have limited access to modern family planning methods.

Article Summary:

Strengths and limitations of this study:

- Examine family planning outcomes for an important but understudied population, women in military camps in a high-fertility setting;
- Use representative data for both military camp and general population in Kinshasa;
- Lacking some measures importantly related to family planning in this environment.

Key words:

Military, fertility, family planning, contraceptive use, Kinshasa, Democratic Republic of Congo.

Word count: 2847

For peer review only - http://bmjopen.bmj.com/site/about/guidelines.xhtml

Introduction

In order to increase knowledge and use of contraceptive methods, programs often target specific sub-populations that have lower contraceptive use and access, such as poor, urban, or adolescent female populations (e.g., Ahmed et al. 2010; Creanga et al. 2011; Wood and Jewkes 2006). These populations then become the subjects for targeted family planning programs, in order to improve overall population-level fertility and family planning outcomes. However, some groups that may differ in family planning outcomes are seldom examined; the military is one such population.

There is reason to believe that the military population is highly relevant for family planning research and programs. In some settings, the military population composes a non-negligible percentage of the overall population and may therefore have a noticeable impact on aggregate family planning measures. The military also may have different family planning practices than the general population: research in the United States has shown that the military population has relatively higher fertility and earlier family formation (Hickes Lundquist and Smith 2005).

The relationship between military participation and fertility is ambiguous. Some research has shown that the military is an environment that promotes family formation, and military members marry earlier and higher fertility (Hickes Lundquist and Smith 2005). Benefits offered for military families can facilitate childbearing (Hickes Lundquist and Smith 2005). As a result, it's perhaps not surprising that most studies show high rates of pregnancy and low contraceptive use among military in the United States (Erickson et al. 2017; Goyal, Borrero, and Schwarz 2012; Harrington, Shaw, and Shaw 2017; Holt et al. 2011).

On the other hand, there are also reasons why one might expect lower fertility and greater contraceptive use among female members of the military. Pregnancy inhibits the ability to train for and serve in active duty (Enewold et al. 2010), and has led to evacuation from military activity for female troops (Gehlbach 1996). Military activity may separate spouses for extended periods, thereby limiting opportunity for childbearing (Karney and Crown 2007). Military service can also cause stress within marriage, which may impact fertility (Karney and Crown

For peer review only - http://bmjopen.bmj.com/site/about/guidelines.xhtml

2007). Some studies have found higher use of oral contraceptive in the military (Enewold et al. 2010).

Existing literature on family planning practices for the military in a developing country context is particularly limited. During the HIV/AIDS epidemic, the military was seen as a population at particular risk of infection and was therefore the topic of HIV-related research (e.g., Djoko et al. 2011; Grillo et al. 2017; Miles 2003; Nwokoji and Ajuwon 2004; Tarimo et al. 2013). But research on family planning among the military in developing settings is limited to few geographic areas, such as India (Hiremath et al. 2017), and Nigeria (Abdulrazaq et al. 2014; Chimah et al. 2016; Hussain et al. 2013; Dogo 1998). The scant existing research has important limitations, such as the lack of a comparison group from the general population, so it's often not known if the military population is at particular need of family planning programs (Abdulrazaq et al. 2014; Hiremath et al. 2017; Chimah et al. 2016; Hussain et al. 2013; Dogo 1998).

In this research we examine family planning-related outcomes among women in military camps in Kinshasa, Democratic Republic of Congo. We focus on a range of family planning outcomes, such as fertility, contraceptive use (overall, modern and traditional), whether the last birth was unintended, desire for an additional child, family planning knowledge, and exposure to family planning messages. We compare family planning outcomes for the military camp population in Kinshasa with a comparable survey of that is representative of the general, non-military population in Kinshasa.

Methods

<u>Setting</u>

The DRC is Africa's fourth most-populous and one of the region's fastest growing countries (United Nations, 2015). DRC has one of the highest fertility rates in the world: the most recent Demographic and Health Survey (DHS) from 2013-14 estimated a country-level TFR of 6.6, a slight increase since the 6.3 TFR estimated from the 2007 DHS (Ministère du Plan et Macro International 2008; Ministère du Plan et Suivi de la Mise en oeuvre de la Révolution de la Modernité (MPSMRM), Ministère de la Santé Publique (MSP) et ICF International 2014). At the same time, contraceptive use is low in DRC: the modern contraceptive prevalence rate

For peer review only - http://bmjopen.bmj.com/site/about/guidelines.xhtml

(mCPR) among women age 15-49 who are married or in union is 7.8% for the country as a whole, 19.0% in Kinshasa and 17.2% in Kongo Central (Ministère du Plan et Suivi de la Mise en oeuvre de la Révolution de la Modernité (MPSMRM), Ministère de la Santé Publique (MSP) et ICF International 2014).

Military camps are located throughout the city of Kinshasa. According to government documents obtained by the authors, there are 17 military camps in Kinshasa, in which both enlisted military and their families reside. According to 2016 estimates, the total population of these camps is 305,405, which represents approximately 3% of the population of Kinshasa. The vast majority of enlisted military are male; only 4% of military members are women. Military camps are closed environments and difficult to access by the civilian population, as such they are often not targeted by mass health activities. Although the majority of enlisted military live in camps, because housing and other services are free, not all members of the military reside there; officers in the military often live outside. All military camps have a health center, which can be used by military members and their families.

Data

We use two sources of data for this analysis. Our first source is the Performance Monitoring and Accountability 2020 Project (PMA2020), which was established in part to measure uptake of contraceptive use in many of the world's most populous countries (http://www.pma2020.org/). To achieve this aim, PMA2020 collects representative data in eleven countries on an annual basis for a range of fertility and family planning-related measures.

To date, PMA2020 has collected data from two provinces in DRC, six rounds of data in Kinshasa (2013-2017), and three rounds in Kongo Central (2015-2017), a province to the west of Kinshasa. The sampling framework uses a two-stage cluster sampling approach, in which the study first randomly selects census enumeration areas within each province, then conducts a listing of all households in these EAs, and randomly selects 33 households within each EA. PMA2020 first administers a household survey to the head of household, and then all resident women of reproductive age (15-49 years) within the household are selected for interview. The PMA2020 female survey includes basic demographic information and extensive information on

For peer review only - http://bmjopen.bmj.com/site/about/guidelines.xhtml

fertility history and preferences, and contraceptive use. In 2016, PMA2020 interviewed 2,607 women in its fifth round of data collection in Kinshasa. Interviews were conducted only with female interviewers.

Data collection for the military sample also took place in 2016. In sampling this population, we used a similar two-stage cluster design approach. Out of the 17 military camps in Kinshasa, ten were randomly drawn, proportionate to population size. These ten military camps were then divided into enumeration areas (or EAs), and one EA was randomly drawn in each of the ten camps. As with PMA2020, interviewers first conducted a listing of households in each EA, after which 33 households in each EA were selected for interview (with all residing women aged 15-49 sought for interview). A total of 514 women were interviewed in the military sample. Of these women, the majority were spouses of men in the military (78.6%), while 16.6% were military members themselves. The remaining women were mothers of a military member.

For the DRC, this study has received approval to collect data from Institutional Review Boards at Johns Hopkins University, Tulane University, and the University of Kinshasa.

Measures

We focus on several categories of family planning outcomes, starting with four fertility-related outcomes: the number of lifetime births, whether the woman experienced the death of one of her children do not want another child, and whether the last birth was unintended. Next we turn to family planning use, measured as overall contraceptive use, then separated into modern and traditional methods. Among those using contraception, we examine whether women obtained their desired method, phrased as "During that visit, did you obtain the method you wanted to delay or avoid getting pregnant?" Finally, we measure family planning awareness and exposure; whether the respondent had heard of injectables, implants, condoms, withdrawal, and rhythm methods; and whether the woman had visited a health facility in the past 12 months, heard about family planning on the radio, read about family planning in the newspaper, or saw family planning billboard.

For peer review only - http://bmjopen.bmj.com/site/about/guidelines.xhtml

We also examine demographic characteristics for our military camp and general population in Kinshasa, including age, number of lifetime births, marital status, level of education, and household wealth. Household wealth is measured using a constructed wealth index based on ownership of 25 household durable assets, house and roof material, livestock ownership and water source. A wealth index was created using principal component analysis (Filmer and Pritchett 2001), which is then converted into quintiles.

Analysis

First, we tabulate background and family planning characteristics for the military and non-military populations. To do so, we account for the sampling design from data collection, using weights and accounting for clustering within enumeration areas.

Then we examine whether family planning differences persist, after controlling for background characteristics that may differ between military and non-military populations. To do so, we use multivariate regressions where the dependent variables are measures of interest for family planning, including number of lifetime births, use of contraception (overall contraceptives, modern contraceptives, traditional methods), not wanting another child, experiencing child mortality, and last birth unintended. Independent variables include age, a quadratic term for age, level of education, marital status, and household wealth quintile. To account for the study design, we cluster standard errors by enumeration area. Missing values are considered missing at random.

Results

Background characteristics (weighted) for women from both rounds of PMA2020 are shown in Table 1, including statistical tests for differences between military and non-military populations. Generally, characteristics of the military and non-military populations are similar. Women in the military population are significantly younger (27.0 years compared to 28.1), but there are no other statistically significant differences in background characteristics.

Although the populations are similar in demographic characteristics, they are very different in family planning-related outcomes (Table 1). Women in the military population are significantly

For peer review only - http://bmjopen.bmj.com/site/about/guidelines.xhtml

For peer review only

less likely to use contraception. Women in military camps are also significantly less likely to obtain their desired contraceptive method. At the same time, as shown in Table 1, the military are more likely to not want another child, and to claim that their last birth was unintended. The military are less informed of family planning methods; they are significantly less likely to have heard of implants, injectables, condoms, withdrawal, and rhythm. However, results for exposure to family planning programs are mixed: they are less likely to have visited a health facility in the past 12 months but more likely to have heard about FP on the radio, read about it in a newspaper, and saw a family planning billboard.

After controlling for demographic characteristics, the multivariate results are similar to the bivariate differences between military and non-military populations. As shown in Table 2, there is no statistically significant difference in number of lifetime births between military and non-military populations. However, the military are significantly less likely to have experienced child mortality than the non-military (OR 0.53, 95% CI 0.326-0.871). The military are significantly more likely to report that they do not want another child (OR 5.17, 95% CI 2.521, 10.619), and to report that their last birth was unintended (OR 5.19, 95% CI 2.314, 11.621).

Results in Table 3 confirm the differences in contraceptive use for these populations, in which the military are significantly less likely to be using contraception of all types, modern methods, and traditional methods (all methods OR 0.24, 95% CI 0.107-0.534; modern methods OR 0.25, 95% CI 0.080-0.789; traditional methods OR 0.41, 95% CI 0.238-0.709). As in the bivariate analysis, women in military camps were also significantly less likely to have obtained their desired family planning method (OR 0.14, 95% CI 0.032, 0.638).

We also find consistent differences in knowledge of various contraceptive methods (Table 4), both modern and traditional. The military women are significantly less likely to have heard of implants (OR 0.23, 95% CI 0.111-0.484), injectables (OR 0.19, 95% CI 0.083-0.438), condoms (OR 0.23, 95% CI 0.117-0.470), withdrawal (OR 0.05, 95% CI 0.016-0.172), and rhythm (OR 0.12, 95% CI 0.033-0.439) methods.

For peer review only - http://bmjopen.bmj.com/site/about/guidelines.xhtml

Results for exposure to family planning are shown in Table 5. Military women are less likely to have visited a health facility in the past 12 months (OR 0.28, 95% CI 0.143-0.552). However, they are significantly more likely to have heard about family planning on the radio (OR 2.81, 95% CI 1.337-5.922), read about family planning in a newspaper or magazine (OR 7.72, 95% CI 3.131-19.042), and saw a family planning billboard (OR 9.71, 95% CI 3.632-25.203).

Discussion

Although the female population in military camps is similar to the general population of women in Kinshasa in background characteristics, we find many strong and highly statistically significant differences in family planning-related outcomes. Overall, these results suggest that the military population is an important, underserved population in Kinshasa: the female population in military camps are more likely to want to limit births but are less likely to be using contraception, and less likely to have obtained their preferred family planning method. The military are more likely to be exposed to family planning messages (radio, billboard, newspaper/magazine), but are less knowledgeable about many family planning methods.

These results suggest that women in military camps have limited access to modern family planning methods. Lack of access would explain why they are less likely to use despite being more likely to want to limit births. Access limitations are also evident in the fact that women in military camps are less likely to have obtained their preferred family planning method, and are less likely to have visited a health facility in the past 12 months. Prior to this study, leadership in the DRC military (co-authors on this paper) recognized a lack of access to and limited use of modern contraception among the military population. They actively sought out a research partner to document this trend and a service delivery organization that could work with them in improving contraceptive services within the military camps of Kinshasa and Kongo Central. Our study confirms the expectations of the DRC military that contraceptive use is lower among the military, due at least in part to limited access to modern family planning.

The lack of access does not, however, explain the lower use or traditional methods and the greater exposure to family planning messaging among the military camp population. This may be explained by the timing of family planning programs. According to our authors, new family

For peer review only - http://bmjopen.bmj.com/site/about/guidelines.xhtml

planning programs were advertised in military camps recently prior to our data collection, but this was not yet matched by supply of family planning methods in military camp clinics.

This study also serves as a baseline for a programmatic initiative that started shortly after the survey in Kinshasa, which consisted of training clinical personnel at the military health center in all contraceptive methods, including Implanon NXT (highly popular in Kinshasa); training and supplying community-health workers with a non-medical profile to distribute pills, condoms and Cyclebeads at the community level; and placing billboards promoting FP near military camps that showed the father in uniform. A follow-up survey is scheduled for 2018 to assess change in FP access and use.

The strengths of this study are the rare opportunity to learn more about a neglected, but important, population for family planning; and the representative data for residents of military camps and general population of Kinshasa. However, there remain some limitations. Several measures of interest are not included in our survey instrument. Many military members and their families are likely in-migrants to Kinshasa from elsewhere in DRC; research in this setting has shown that migrants differ from Kinshasa-born residents in many characteristics related to family planning (Anglewicz, Corker, and Kayembe 2017). This study, unfortunately, did not measure migration status, birthplace, or duration resided in Kinshasa. Similarly, measures of access to a facility that provides family planning, such as distance to the nearest family planning clinic or pharmacy that has modern methods in stock, were not measured for the military camp population, but may differ from the general population- as suggested by our results.

Contributors: PA, PA and JB initially conceived the manuscript. PA and PA conducted the statistical analysis and wrote the first draft of the paper. HNE, GKK, JH, PK, and JB reviewed the paper before submission and provided comments and edits.

Funding statement: PMA2020 was supported by the Bill & Melinda Gates Foundation, Seattle, WA; under grant #OPP1079004. Data collection for military camps in Kinshasa was also supported by the Bill & Melinda Gates Foundation, Seattle, WA; under grant # OPP1128892.

For peer review only - http://bmjopen.bmj.com/site/about/guidelines.xhtml

Competing interests statement: the authors declare no competing interests.

Data sharing statement: All data collected under PMA2020 is made publically-available. More details on data access for PMA2020 is available here: http://www.pma2020.org/data-use.

For peer review only

For peer review only - http://bmjopen.bmj.com/site/about/guidelines.xhtml

Figures and Tables

Table 1: Weighted background characteristics for military and non-military women, Kinshasa 2016

	Non-military	Military
**Mean age**	28.1	27.0*
**Level of education**		
No education	2.1%	4.3%
Primary	18.6%	13.5%
Middle secondary school	63.6%	66.8%
Advanced secondary or higher	15.7%	15.4%
**Marital status**		
Married/partnered	47.0%	48.2%
Separated/divorced/widowed	5.3%	4.8%
Never married	47.7%	47.0%
**Wealth index**		
Quintile 1 (lowest)	15.1%	26.4%
Quintile 2	19.3%	27.0%
Quintile 3	21.3%	20.7%
Quintile 4	21.3%	15.1%
Quintile 5 (highest)	23.0%	10.8%
**Fertility and children**		
Number lifetime births	1.8	1.7
Experienced child mortality	19.7%	12.4%*
Do not want another child	18.0%	46.7%**
Last birth unintended	7.4%	26.3%**
**Family planning use**		
Contraceptive use	42.3%	17.4%**
Traditional contraceptive use	21.4%	6.6%*
Modern contraceptive use	20.9%	10.9%**
Obtained desired the FP method	92.4%	68.3%**
**Family planning knowledge**		
Heard of implants	83.3%	55.8%**
Heard of injectables	87.8%	60.9%**
Heard of condoms	94.7%	81.9%**
Heard of withdrawal	80.8%	46.9%**
Heard of rhythm	91.5%	44.6%**
**Family planning exposure**		
Visited health facility in past 12 months	55.2%	28.8%**
Heard about FP on the radio	34.7%	58.6%*
Read about FP in a magazine/newspaper	13.1%	50.8%**
Saw FP billboard advertisement	46.1%	89.3%**
N=	2607	514

Notes: Difference between military and non-military significant at $*p \leq 0.05$; $**p \leq 0.01$.

For peer review only

Table 2: Weighted regression results for differences in fertility and children between military and non-military, Kinshasa 2016

	Number of births		Child mortality		Do not want another		Last birth unintended	
	**Coef**	**95% CI**	**Odds**	**95% CI**	**Odds**	**95% CI**	**Odds**	**95% CI**
**Military**	**-0.05**	**-0.310, 0.215**	**0.53***	**0.326, 0.871**	**5.17****	**2.521, 10.619**	**5.19****	**2.314, 11.621**
Number of births	----	----	1.16**	1.048, 1.292	1.55**	1.313, 1.840	1.43**	1.225, 1.661
Age	0.33**	0.280, 0.373	1.03**	1.011, 1.056	1.02*	0.999, 1.050	0.99	0.961, 1.026
Age squared	-0.01**	-0.004, -0.003	----	----	----	----	----	----
Level of education								
No education (ref.)	----	----	----	----	----	----	----	----
Primary	0.13	-0.125, 0.381	1.05	0.501, 2.204	0.57	0.140, 1.067	1.01	0.140, 1.067
Middle secondary school	0.03	-0.276, 0.334	0.81	0.344, 1.925	0.48	0.198, 1.157	0.51	0.193, 1.321
Advanced secondary or higher	-0.37*	-0.729, -0.019	0.43	0.157, 1.177	0.39	0.140, 1.067	0.67	0.228, 1.977
Marital status								
Married/partnered (ref.)	----	----	----	----	----	----	----	----
Separated/divorced/widowed	-0.16**	-0.278, -0.051	0.72	0.404, 1.294	0.88	0.509, 1.529	2.82**	1.467, 5.416
Never married	-1.06**	-1.318, -0.795	1.03	0.669, 1.593	1.06	0.643, 1.745	2.81*	1.186, 6.661
Household wealth								
Quintile 1 (lowest, ref.)	----	----	----	----	----	----	----	----
Quintile 2	-0.13	-0.272, 0.011	0.85	0.480, 1.502	0.94	0.515, 1.628	0.54	0.197, 1.479
Quintile 3	-0.16*	-0.312, -0.007	0.88	0.568, 1.372	0.92	0.187, 1.258	0.69	0.262, 1.827
Quintile 4	-0.12	-0.286, 0.040	0.67	0.410, 1.096	0.48	0.307, 1.216	1.01	0.470, 2.158
Quintile 5 (highest)	-0.23**	-0.401, -0.063	0.77	0.438, 1.345	0.61	0.499, 1.644	0.82	0.296, 2.266
N=	3085		1795		2830		1813	

Notes: *p ≤ 0.05; **p ≤ 0.01; child mortality and last birth unintended are limited to women who have ever given birth.

For peer review only

Table 3: Weighted logistic regression results for differences in family planning use for military, Kinshasa 2016

	Using Contraception		Modern Contraception		Traditional Contraception		Obtained desired method	
	**Odds**	**95% CI**	**Odds**	**95% CI**	**Odds**	**95% CI**	**Odds**	**95% CI**
**Military**	**0.24****	**0.107, 0.534**	**0.25***	**0.080, 0.789**	**0.41****	**0.238, 0.709**	**0.14****	**0.032, 0.638**
Number of births	1.29**	1.165, 1.431	1.16**	1.048, 1.281	1.22**	1.130, 1.322	0.98	0.798, 1.195
Age	1.53**	1.426, 1.639	1.23**	1.138, 1.321	1.60**	1.459, 1.752	0.99	0.770, 1.276
Age quadratic	0.99**	0.992, 0.994	0.99**	0.996, 0.998	0.99**	0.991, 0.994	1.00	0.997, 1.004
Level of education								
No education (ref.)								
Primary	1.68	0.748, 3.781	0.70	0.351, 1.408	3.41*	1.243, 9.330	1.59	0.253, 9.952
Middle secondary school	2.33*	1.031, 5.273	1.16	0.551, 2.453	3.42*	1.298, 9.003	1.69	0.269, 10.627
Advanced secondary or higher	3.16**	1.326, 7.528	1.55	0.649, 3.709	3.98**	1.441, 11.020	1.13	0.159, 8.002
Marital status								
Married/partnered (ref.)								
Separated/divorced/widowed	0.45**	0.269, 0.751	0.41*	0.203, 0.812	0.74	0.455, 1.194	0.77	0.352, 1.707
Never married	1.52*	1.102, 2.110	1.16	0.825, 1.630	1.55**	1.119, 2.134	1.34	0.750, 2.391
Household wealth								
Quintile 1 (lowest, ref.)								
Quintile 2	0.91	0.451, 1.830	1.27	0.698, 2.296	0.73	0.409, 1.304	0.46	0.126, 1.698
Quintile 3	0.82	0.405, 1.656	1.17	0.573, 2.381	0.68	0.405, 1.140	0.45	0.114, 1.773
Quintile 4	0.99	0.469, 2.109	1.29	0.598, 2.794	0.81	0.495, 1.325	0.28	0.065, 1.183
Quintile 5 (highest)	0.68	0.343, 1.341	0.96	0.480, 1.934	0.62	0.360, 1.071	0.32*	0.082, 0.915
N=	3072		3072		3072		1322	

Notes: *p ≤ .05; **p ≤ .01; analysis for obtaining desired method is limited to women currently using contraception.

For peer review only - http://bmjopen.bmj.com/site/about/guidelines.xhtml

Table 4: Weighted logistic regression results for differences in family planning knowledge among military, Kinshasa 2016

	Heard of implants		Heard of injectables		Heard of condoms		Heard of withdrawal		Heard of rhythm	
	Odds	95% CI	Odds	95% CI	Odds	95% CI	Odds	95% CI	Odds	95% CI
**Military**	**0.23****	**0.111, 0.484**	**0.19****	**0.083, 0.438**	**0.23****	**0.117, 0.470**	**0.05****	**0.016, 0.172**	**0.12****	**0.033, 0.439**
Number of births	1.06	0.949, 1.191	1.27**	1.077, 1.500	0.83*	0.719, 0.965	1.09	0.906, 1.317	1.11	0.925, 1.337
Age	1.49**	1.314, 1.695	1.31**	1.203, 1.434	1.47**	1.262, 1.717	1.27**	1.115, 1.451	1.57**	1.389, 1.767
Age squared	0.99**	0.992, 0.996	0.99**	0.995, 0.997	0.99**	0.993, 0.998	0.99**	0.995, 0.999	0.99**	0.992, 0.996
Level of education										
No education (ref.)	----	----	----	----	----	----	----	----	----	----
Primary	3.01**	1.401, 6.450	1.12	0.641, 1.954	0.36	0.069, 1.901	3.14**	1.579, 6.242	2.40**	1.304, 4.407
Middle secondary school	5.51**	2.316, 13.091	3.15**	1.595, 6.216	0.97	0.132, 7.183	7.85**	3.107, 19.848	4.29**	1.973, 9.306
Advanced secondary or higher	10.85**	4.110, 28.624	6.23**	2.344, 16.556	6.57	0.547, 78.843	27.43**	5.770, 130.444	12.80**	3.739, 43.806
Marital status										
Married/partnered (ref.)	----	----	----	----	----	----	----	----	----	----
Separated/divorced/widowed	1.18	0.557, 2.479	0.96	0.394, 2.359	0.97	0.244, 3.868	1.21	0.629, 2.343	1.44	0.567, 3.635
Never married	0.65*	0.456, 0.938	0.89	0.562, 1.412	1.27	0.582, 2.751	0.95	0.675, 1.331	0.63	0.369, 1.075
Household wealth										
Quintile 1 (lowest, ref.)	----	----	----	----	----	----	----	----	----	----
Quintile 2	0.95	0.484, 1.879	0.83	0.450, 1.541	0.75	0.272, 2.073	0.38**	0.180, 0.788	0.41*	0.182, 0.921
Quintile 3	0.79	0.386, 1.624	0.86	0.419, 1.745	0.99	0.380, 2.604	0.45*	0.203, 0.980	0.48	0.205, 1.142
Quintile 4	0.68	0.325, 1.427	0.86	0.440, 1.689	0.65	0.274, 1.531	0.77	0.305, 1.928	0.60	0.264, 1.378
Quintile 5 (highest)	0.72	0.302, 1.706	0.64	0.281, 1.480	1.06	0.372, 3.007	0.60	0.245, 1.474	0.41*	0.185, 0.925
N=	3078		3077		3080		3079		3077	

Notes: *p ≤ 0.05; **p ≤ 0.01.

For peer review only - http://bmjopen.bmj.com/site/about/guidelines.xhtml

Table 5: Weighted logistic regression results for differences in family planning exposure among military, Kinshasa 2016

	Visited health facility		Heard about FP- radio		Read about FP		FP billboard	
	Odds	95% CI	Odds	95% CI	Odds	95% CI	Odds	95% CI
**Military**	**0.28****	**0.143, 0.552**	**2.81****	**1.337, 5.922**	**7.72****	**3.131, 19.042**	**9.71****	**3.632, 25.203**
Number of births	1.07	0.999, 1.148	0.96	0.900, 1.029	0.86*	0.767, 0.966	0.97	0.897, 1.046
Age	1.11**	1.042, 1.172	1.11*	1.026, 1.199	1.12	0.996, 1.252	1.11**	1.046, 1.178
Age squared	0.99**	0.997, 0.999	1.00	0.998, 1.000	1.00	0.997, 1.000	0.99**	0.998, 1.000
Level of education								
No education (ref.)	----	----	----	----	----	----	----	----
Primary	1.21	0.604, 2.420	3.30**	1.520, 7.181	3.17	0.956, 10.495	1.22	0.602, 2.558
Middle secondary school	1.36	0.673, 2.764	6.00**	2.330, 15.477	6.05**	1.800, 20.335	1.34	0.747, 2.484
Advanced secondary or higher	1.91	0.850, 4.284	13.93**	4.884, 39.738	15.99**	4.262, 59.995	2.18*	1.122, 4.298
Marital status								
Married/partnered (ref.)	----	----	----	----	----	----	----	----
Separated/divorced/widowed	1.05	0.624, 1.756	0.89	0.574, 1.382	1.00	0.554, 1.801	0.94	0.557, 1.659
Never married	0.53**	0.415, 0.679	0.84	0.591, 1.182	0.82	0.548, 1.242	1.03	0.738, 1.417
Household wealth								
Quintile 1 (lowest, ref.)	----	----	----	----	----	----	----	----
Quintile 2	0.52**	0.328, 0.828	1.05	0.550, 2.000	0.81	0.357, 1.848	1.03	0.622, 1.692
Quintile 3	0.55*	0.345, 0.882	0.86	0.432, 1.722	0.58	0.249, 1.364	1.08	0.630, 1.942
Quintile 4	0.48**	0.312, 0.753	0.84	0.430, 1.660	0.47	0.182, 1.204	0.85	0.529, 1.487
Quintile 5 (highest)	0.59*	0.370, 0.950	0.71	0.300, 1.690	0.73	0.243, 2.202	0.91	0.502, 1.766
N=	3079		3072		3042		3072	

Notes: *p ≤ 0.05; **p ≤ 0.01.

For peer review only - http://bmjopen.bmj.com/site/about/guidelines.xhtml

References

Abdulrazaq, A. G., S. Kabir, N. S. Mohammad, and I. H. Suleiman. 2014. "The Effect of Educational Intervention on Family Planning Knowledge, Attitudes, and Practices among Married Women in a Military Barrack in Northern Nigeria." *African Journal of Reproductive Health* 18 (1): 93–101.

Ahmed, Saifuddin, Andreea A. Creanga, Duff G. Gillespie, and Amy O. Tsui. 2010. "Economic Status, Education and Empowerment: Implications for Maternal Health Service Utilization in Developing Countries." *PloS One* 5 (6): e11190.

Anglewicz, Philip, Jamaica Corker, and Patrick Kayembe. 2017. "The Fertility of Internal Migrants to Kinshasa." *Genus* 1 (73): 1–18.

Chimah, U. C., T. O. Lawoyin, A. L. Ilika, and C. C. Nnebue. 2016. "Contraceptive Knowledge and Practice among Senior Secondary Schools Students in Military Barracks in Nigeria." *Nigerian Journal of Clinical Practice* 19 (2): 182–188.

Creanga, Andreea A., Duff Gillespie, Sabrina Karklins, and Amy O. Tsui. 2011. "Low Use of Contraception among Poor Women in Africa: An Equity Issue." *Bulletin of the World Health Organization* 89 (4): 258–266.

Djoko, Cyrille F., Anne W. Rimoin, Nicole Vidal, Ubald Tamoufe, Nathan D. Wolfe, Christelle Butel, Matthew LeBreton, et al. 2011. "High HIV Type 1 Group M Pol Diversity and Low Rate of Antiretroviral Resistance Mutations Among the Uniformed Services in Kinshasa, Democratic Republic of the Congo." *AIDS Research and Human Retroviruses* 27 (3): 323–29. https://doi.org/10.1089/aid.2010.0201.

Dogo, Bala. 1998. "A Survey of Knowledge, Attitude and Practice of Family Planning among the Military in the Rukuba Cantonment of Kaduna." *Journal of Environmental Sciences* 1 (2): 124–132.

Enewold, Lindsey, Louise A. Brinton, Katherine A. McGlynn, Shelia H. Zahm, John F. Potter, and Kangmin Zhu. 2010. "Oral Contraceptive Use among Women in the Military and the General US Population." *Journal of Women's Health* 19 (5): 839–845.

Erickson, Anne K., D. Alan Nelson, Jonathan G. Shaw, Pooja D. Loftus, Lianne M. Kurina, and Kate A. Shaw. 2017. "Long-Acting Reversible Contraceptive Placement Among Active-Duty U.S. Army Servicewomen." *Obstetrics and Gynecology* 129 (5): 800–809. https://doi.org/10.1097/AOG.0000000000001971.

Filmer, Deon, and Lant H. Pritchett. 2001. "Estimating Wealth Effects without Expenditure Data—or Tears: An Application to Educational Enrollments in States of India." *Demography* 38 (1): 115–132.

Gehlbach, D. 1996. "Contraceptive Needs Complications and New Directions for Research." *Womens Health Issues* 6 (6): 355–8.

Goyal, Vinita, Sonya Borrero, and Eleanor Bimla Schwarz. 2012. "Unintended Pregnancy and Contraception among Active-Duty Servicewomen and Veterans." *American Journal of Obstetrics & Gynecology* 206 (6): 463–69. https://doi.org/10.1016/j.ajog.2011.11.018.

Grillo, Michael, Margo Sloan, Che Wankie, Kelly Woodland, Elizabeth Reader, Bruce Porter, Caroline A Macera, and Richard A Shaffer. 2017. "Prevention Interventions for People Living with HIV in Military Settings." *Current HIV Research* 15 (2): 90–94.

Harrington, Lauren A., Kate A. Shaw, and Jonathan G. Shaw. 2017. "Contraception in US Servicewomen: Emerging Knowledge, Considerations, and Needs." *Current Opinion in Obstetrics & Gynecology*, September. https://doi.org/10.1097/GCO.0000000000000414.

For peer review only - http://bmjopen.bmj.com/site/about/guidelines.xhtml

Hickes Lundquist, Jennifer, and Herbert L. Smith. 2005. "Family Formation among Women in the US Military: Evidence from the NLSY." *Journal of Marriage and Family* 67 (1): 1–13.

Hiremath, R. N., A. K. Yadav, Sandhya Ghodke, and Jyoti Yadav. 2017. "Contraceptive Use and Its Determinants amongst Armed Forces Personnel." *Medical Journal Armed Forces India*. http://www.sciencedirect.com/science/article/pii/S0377123716302064.

Holt, Kelsey, Kate Grindlay, Madeline Taskier, and Daniel Grossman. 2011. "Unintended Pregnancy and Contraceptive Use among Women in the U.S. Military: A Systematic Literature Review." *Military Medicine* 176 (9): 1056–64.

Hussain, N. A., T. M. Akande, G. K. Osagbemi, S. T. Olasupo, K. Y. Salawu, and E. T. Adebayo. 2013. "Perception and Practice of Contraception among Male Soldiers in Sobi Barracks, Ilorin, Nigeria." *African Health Sciences* 13 (2): 415–422.

Karney, Benjamin R., and John S. Crown. 2007. *Families Under Stress: An Assessment of Data, Theory, and Reseach on Marriage and Divorce in the Military*. Rand Corporation.

Miles, Steven H. 2003. "HIV in Insurgency Forces in Sub-Saharan Africa—a Personal View of Policies." *International Journal of Std & Aids* 14 (3): 174–178.

Ministère du Plan et Macro International. 2008. *Enquête Démographique et de Santé, République Démocratique Du Congo 2007*. Calverton, Maryland, U.S.A.: Ministère du Plan et Macro International.

Ministère du Plan et Suivi de la Mise en oeuvre de la Révolution de la Modernité (MPSMRM), Ministère de la Santé Publique (MSP) et ICF International. 2014. *Enquête Démographique et de Santé En République Démocratique Du Congo 2013-2014.* Rockville, Maryland, USA: MPSMRM, MSP et ICF International.

Nwokoji, Ugboga Adaji, and Ademola J. Ajuwon. 2004. "Knowledge of AIDS and HIV Risk-Related Sexual Behavior among Nigerian Naval Personnel." *BMC Public Health* 4 (1): 24.

Tarimo, Edith AM, Thecla W. Kohi, Muhammad Bakari, and Asli Kulane. 2013. "A Qualitative Study of Perceived Risk for HIV Transmission among Police Officers in Dar Es Salaam, Tanzania." *BMC Public Health* 13 (1): 785.

United Nations. n.d. *World Population Prospects: The 2015 Revision, Volume II: Demographic Profiles*. 2015th ed. United Nations, New York, NY.

Wood, Kate, and Rachel Jewkes. 2006. "Blood Blockages and Scolding Nurses: Barriers to Adolescent Contraceptive Use in South Africa." *Reproductive Health Matters* 14 (27): 109–118.

For peer review only

For peer review only - http://bmjopen.bmj.com/site/about/guidelines.xhtml

BMJ Open

Differences in Family Planning Outcomes between Military and General Populations in Kinshasa, Democratic Republic of Congo: A Cross-Sectional Analysis

Journal:	*BMJ Open*
Manuscript ID	bmjopen-2018-022295.R1
Article Type:	Research
Date Submitted by the Author:	06-Jul-2018
Complete List of Authors:	Akilimali, Pierre; Universite de Kinshasa, Kinshasa School of Public Health<br>Anglewicz, Philip; Tulane University School of Public Health and Tropical Medicine, Department of Global Community Health and Behavioral Science;<br>Engale, Henri; Congolese Armed Forces, Medical Division<br>Kurhenga, Gilbert; Congolese Armed Forces, Medical Division<br>Hernandez, Julie; Tulane University, School of Public Health and Tropical Medicine<br>Kayembe, Patrick; University of Kinshasa, Kinshasa School of Public Health<br>Bertrand, Jane; Tulane University
**Primary Subject Heading**:	Global health
Secondary Subject Heading:	Reproductive medicine, Public health
Keywords:	Military, Fertility, Family planning, Contraceptive use, Kinshasa, Democratic Republic of Congo

SCHOLARONE™
Manuscripts

Differences in Family Planning Outcomes between Military and General Populations in Kinshasa, Democratic Republic of Congo: A Cross-Sectional Analysis

Authors

Pierre Akilimali
Kinshasa School of Public Health, University of Kinshasa, BP 11850 Kinshasa 1, Democratic Republic of Congo. Email: pierretulanefp@gmail.com.

Philip Anglewicz (corresponding author)
School of Public Health and Tropical Medicine, Tulane University, 1440 Canal St Suite 2210, New Orleans, LA, USA. Email: panglewi@tulane.edu Phone: (504) 988-5428; Fax: (504) 988-5480.

Henri Nzuka Engale
Medical Division, Congolese Armed Forces, Email: nzukaengale@yahoo.fr

Gilbert Kabanda Kurhenga
Chief, Medical division, Congolese Armed Forces, Email: gilkaku@yahoo.fr

Julie Hernandez
School of Public Health and Tropical Medicine, Tulane University, 1440 Canal St Suite 1900, New Orleans, LA, USA. Email: jhernan7@tulane.edu

Patrick Kayembe
Kinshasa School of Public Health, University of Kinshasa, BP 11850 Kinshasa 1, Democratic Republic of Congo. Email: patkayembe@yahoo.fr

Jane Bertrand
School of Public Health and Tropical Medicine, Tulane University, 1440 Canal St Suite 1900, New Orleans, LA, USA. Email: bertrand@tulane.edu

For peer review only

For peer review only - http://bmjopen.bmj.com/site/about/guidelines.xhtml

Abstract:

Objectives: To examine family planning outcomes among women living in military camps in Kinshasa, Democratic Republic of Congo, and compare these outcomes with a representative sample of non-military women in Kinshasa.

Participants: Women of reproductive ages, 15 to 49. We compare two populations: women living in military camps and the general (non-military) population in Kinshasa.

Study Design: For sampling, we used a two-stage cluster sampling design, where we first randomly selected enumeration areas (EA), and then randomly selected women within each EA (separately for each of the two populations). We administered a survey on contraceptive use and family planning to all participating women. We use bivariate and multivariate analysis to compare these populations for a range of family planning outcomes.

Results: We find many statistically significant differences between women in military camps and general female population of Kinshasa. Although they do not have more children, women in military camps are less likely to be using contraception (all methods OR 0.24, 95% CI 0.11-0.53; modern methods OR 0.25, 95% CI 0.08-0.79; traditional methods OR 0.41, 95% CI 0.24-0.71) and less knowledgeable about many family planning methods (less likely to have heard of implants (OR 0.23, 95% CI 0.11-0.48), injectables (OR 0.19, 95% CI 0.08-0.44), condoms (OR 0.23, 95% CI 0.12-0.47), withdrawal (OR 0.05, 95% CI 0.02-0.17), and rhythm (OR 0.12, 95% CI 0.03-0.44) methods), while at the same time they are more likely to want to limit their births (OR 5.17, 95% CI 2.52, 10.62), and less likely to have obtained their preferred family planning method (OR 0.14, 95% CI 0.03, 0.64).

Conclusions: Women in military camps in Kinshasa appear to be an important and underserved population with regards to family planning. Our results suggest that women in military camps have limited access to modern family planning methods.

Article Summary:

Strengths and limitations of this study:

- Examine family planning outcomes for an important but understudied population, women in military camps in a high-fertility setting;
- Use representative data for both military camp and general population in Kinshasa;
- Lacking some measures importantly related to family planning in this environment.

Key words:

Military, fertility, family planning, contraceptive use, Kinshasa, Democratic Republic of Congo.

Word count: 2922

For peer review only - http://bmjopen.bmj.com/site/about/guidelines.xhtml

Introduction

Research on family planning practices among the military population in developing countries is limited. During the HIV/AIDS epidemic, the military was seen as a population at particular risk of infection and was therefore the topic of HIV-related research (e.g., [1–8]). But research on family planning among the military in developing settings has been conducted in few geographic areas, such as India,[9] and Nigeria.[8,10–13] Furthermore, the scant existing research has important limitations, such as the lack of a comparison group from the general population, so it's often not known if the military population is at particular need of family planning programs.[8-13]

In order to increase knowledge and use of contraceptive methods, programs often target specific sub-populations that have lower contraceptive use and access, such as poor, urban, or adolescent female populations (e.g., [14–16]). These populations then become the subjects for targeted family planning programs, in order to improve overall population-level fertility and family planning outcomes. However, some groups that may differ in family planning outcomes are seldom examined; the military is one such population.

There is reason to believe that the military population is highly relevant for family planning research and programs. In some settings, the military population composes a non-negligible percentage of the overall population and may therefore have a noticeable impact on aggregate family planning measures. The military also may have different family planning practices than the general population: research in the United States has shown that the military population has relatively higher fertility and earlier family formation.[17]

The relationship between military participation and fertility is ambiguous. Some research has shown that the military is an environment that promotes family formation, and military members marry earlier and higher fertility.[17] Benefits offered for military families can facilitate childbearing.[17] As a result, it's perhaps not surprising that most studies show high rates of pregnancy and low contraceptive use among military in the United States.[18–21]

For peer review only - http://bmjopen.bmj.com/site/about/guidelines.xhtml

For peer review only

There are also reasons why one might expect lower fertility and greater contraceptive use among female members of the military. Pregnancy inhibits the ability to train for and serve in active duty,[22] and has led to evacuation from military activity for female troops.[23] Military activity may separate spouses for extended periods, thereby limiting opportunity for childbearing.[24] Military service can also cause stress within marriage, which may impact fertility.[24] Some studies have found higher use of oral contraceptive in the military.[22]

In this research we examine family planning-related outcomes among women in military camps in Kinshasa, Democratic Republic of Congo. We focus on a range of family planning outcomes, such as fertility, contraceptive use (overall, modern and traditional), whether the last birth was unintended, desire for an additional child, family planning knowledge, and exposure to family planning messages. We compare family planning outcomes for the female military camp population in Kinshasa with a comparable survey of that is representative of the general, non-military female population in Kinshasa.

Methods

Setting

The DRC is Africa's fourth most-populous and one of the region's fastest growing countries.[25] DRC has one of the highest fertility rates in the world: the most recent Demographic and Health Survey (DHS) from 2013-14 estimated a country-level TFR of 6.6, a slight increase since the 6.3 TFR estimated from the 2007 DHS.[26-27] At the same time, contraceptive use is low in DRC: the modern contraceptive prevalence rate (mCPR) among women age 15-49 who are married or in union is 7.8% for the country as a whole, 19.0% in Kinshasa and 17.2% in Kongo Central.[27]

Military camps are located throughout the city of Kinshasa. According to government documents obtained by the authors, there are 17 military camps in Kinshasa, in which both enlisted military and their families reside. According to 2016 estimates, the total population of these camps is 305,405, which represents approximately 3% of the population of Kinshasa. The vast majority of enlisted military are male; only 4% of military members are women. Military camps are closed environments and difficult to access by the civilian population, as such they are often not targeted by mass health activities. Although the majority of enlisted military live in

For peer review only - http://bmjopen.bmj.com/site/about/guidelines.xhtml

camps, because housing and other services are free, not all members of the military reside there; officers in the military often live outside. All military camps have a health center, which can be used by military members and their families.

Data

We use two sources of data for this analysis. Our first source is the Performance Monitoring and Accountability 2020 Project (PMA2020), which was established in part to measure uptake of contraceptive use in many of the world's most populous countries (http://www.pma2020.org/). To achieve this aim, PMA2020 collects representative data in eleven countries on an annual basis for a range of fertility and family planning-related measures.

PMA2020 has collected data from two provinces in DRC, six rounds of data in Kinshasa (2013-2017), and three rounds in Kongo Central (2015-2017), a province to the west of Kinshasa. The sampling framework uses a two-stage cluster sampling approach, in which the study first randomly selects census enumeration areas within each province, then conducts a listing of all households in these EAs, and randomly selects 33 households within each EA. PMA2020 first administers a household survey to the head of household, and then all resident women of reproductive age (15-49 years) within the household are selected for interview. The PMA2020 female survey includes basic demographic information and extensive information on fertility history and preferences, and contraceptive use. In 2016, PMA2020 interviewed 2,607 women in its fifth round of data collection in Kinshasa. Interviews were conducted only with female interviewers.

Data collection for the military sample also took place in 2016. In sampling this population, we used a similar two-stage cluster design approach. Out of the 17 military camps in Kinshasa, ten were randomly drawn, proportionate to population size. These ten military camps were then divided into enumeration areas (or EAs), and one EA was randomly drawn in each of the ten camps. As with PMA2020, interviewers first conducted a listing of households in each EA, after which 33 households in each EA were selected for interview (with all residing women aged 15-49 sought for interview). A total of 514 women were interviewed in the military sample. Of

For peer review only - http://bmjopen.bmj.com/site/about/guidelines.xhtml

these women, the majority were spouses of men in the military (78.6%), while 16.6% were military members themselves. The remaining women were mothers of a military member.

This study has received approval to collect data from Institutional Review Boards at Johns Hopkins University, Tulane University, and the University of Kinshasa. All participating women provided written and informed consent to take part in this study. In this setting, individuals aged 15-17 are considered adults, so parental consent was not necessary to interview women of these ages.

Measures

We focus on several categories of family planning outcomes, starting with four fertility-related outcomes: the number of lifetime births, whether the woman experienced the death of one of her children, whether the women does not want another child, and whether the last birth was unintended. Next, we turn to family planning use, measured as overall contraceptive use, then separated into modern and traditional methods (rhythm, withdrawal, and other traditional method (folkloric methods like amulets, herbs, etc…)). Among those using contraception, we examine whether women obtained their desired method, phrased as "During that visit, did you obtain the method you wanted to delay or avoid getting pregnant?" Finally, we measure family planning awareness and exposure; whether the respondent had heard of injectables, implants, condoms, withdrawal, and rhythm methods; and whether the woman had visited a health facility in the past 12 months, heard about family planning on the radio, read about family planning in the newspaper, or saw family planning billboard.

We also examine demographic characteristics for women in military camps and general female population in Kinshasa, including age, number of lifetime births, marital status, level of education, and household wealth. Household wealth is measured using a constructed wealth index based on ownership of 25 household durable assets, house and roof material, livestock ownership and water source. A wealth index was created using principal component analysis,[28] which is then converted into quintiles. Survey instruments are included as supplementary files (Supplementary file 1 Female survey- English and Supplementary file 2 Household survey- English).

For peer review only - http://bmjopen.bmj.com/site/about/guidelines.xhtml

Analysis

First, we tabulate background and family planning characteristics for women in military camps and the female non-military population of Kinshasa. We account for the sampling design from data collection, using weights and accounting for clustering within enumeration areas.

Next, we examine whether family planning differences persist, after controlling for background characteristics that may differ between women in military camps and the non-military population. We use multivariate regressions where the dependent variables are measures of interest for family planning, including number of lifetime births, use of contraception (overall contraceptives, modern contraceptives, traditional methods), not wanting another child, experiencing child mortality, and last birth unintended. Independent variables include age, a quadratic term for age, level of education, marital status, and household wealth quintile. To account for the study design, we cluster standard errors by enumeration area. Missing values are considered missing at random.

Patient and Public Involvement

Patients or public were not involved in the study design, development of the research question, recruitment into or conduct of the study, or outcome measures. Results will not be distributed to the participants themselves.

Results

Background characteristics (weighted) for women from both rounds of PMA2020 are shown in Table 1, including statistical tests for differences between women in military camps and the non-military population. Generally, women in military camps and the non-military population are similar in their demographic profile, there are no statistically significant differences in background characteristics.

Although the populations are similar in demographic characteristics, they are very different in family planning-related outcomes (Table 1). Women in military camps are significantly less likely to use contraception, and are significantly less likely to obtain their desired contraceptive

For peer review only - http://bmjopen.bmj.com/site/about/guidelines.xhtml

method. As shown in Table 1, women in military camps are more likely to not want another child, and to claim that their last birth was unintended. Women in military camps are less informed of family planning methods; they are significantly less likely to have heard of implants, injectables, condoms, withdrawal, and rhythm. However, results for exposure to family planning programs are mixed: they are less likely to have visited a health facility in the past 12 months but more likely to have heard about FP on the radio, read about it in a newspaper, and saw a family planning billboard.

After controlling for demographic characteristics, the multivariate results are similar to the bivariate differences between women in military camps and the non-military population. As shown in Table 2, there is no statistically significant difference in number of lifetime births between women in military camps and the non-military population. However, women in military camps are significantly less likely to have experienced child mortality than the non-military (OR 0.53, 95% CI 0.33-0.87). Women in military camps are significantly more likely to report that they do not want another child (OR 5.17, 95% CI 2.52, 10.62), and to report that their last birth was unintended (OR 5.19, 95% CI 2.31, 11.62).

Results in Table 3 confirm the differences in contraceptive use for these populations, in which women in military camps are significantly less likely to be using contraception of all types, modern methods, and traditional methods (all methods OR 0.24, 95% CI 0.11-0.53; modern methods OR 0.25, 95% CI 0.08-0.79; traditional methods OR 0.41, 95% CI 0.24-0.71). Women in military camps were also significantly less likely to have obtained their desired family planning method (OR 0.14, 95% CI 0.03, 0.64).

We also find consistent differences in knowledge of various contraceptive methods (Table 4), both modern and traditional. Women in military camps are significantly less likely to have heard of implants (OR 0.23, 95% CI 0.11-0.48), injectables (OR 0.19, 95% CI 0.08-0.44), condoms (OR 0.23, 95% CI 0.12-0.47), withdrawal (OR 0.05, 95% CI 0.02-0.17), and rhythm (OR 0.12, 95% CI 0.03-0.44) methods.

For peer review only - http://bmjopen.bmj.com/site/about/guidelines.xhtml

Results for exposure to family planning are shown in Table 5. Women in military camps are less likely to have visited a health facility in the past 12 months (OR 0.28, 95% CI 0.14-0.55). However, they are significantly more likely to have heard about family planning on the radio (OR 2.81, 95% CI 1.34-5.92), read about family planning in a newspaper or magazine (OR 7.72, 95% CI 3.13-19.04), and saw a family planning billboard (OR 9.71, 95% CI 3.63-25.20).

Discussion

Although the female population in military camps is similar to the general population of women in Kinshasa in background characteristics, we find many strong and highly statistically significant differences in family planning-related outcomes. Overall, these results suggest that the population residing in military camps is an important, underserved population in Kinshasa: the female population in military camps are more likely to want to limit births but are less likely to be using contraception, and less likely to have obtained their preferred family planning method. Women in military camps are more likely to be exposed to family planning messages (radio, billboard, newspaper/magazine), but are less knowledgeable about many family planning methods.

These results suggest that women in military camps have limited access to modern family planning methods. Lack of access would explain why they are less likely to use despite being more likely to want to limit births. Access limitations are also evident in the fact that women in military camps are less likely to have obtained their preferred family planning method, and are less likely to have visited a health facility in the past 12 months. Prior to this study, leadership in the DRC military (co-authors on this paper) recognized a lack of access to and limited use of modern contraception among the military population. They actively sought out a research partner to document this trend and a service delivery organization that could work with them in improving contraceptive services within the military camps of Kinshasa and Kongo Central. Our study confirms the expectations of the DRC military that contraceptive use is lower among women in military camps, due at least in part to limited access to modern family planning.

The lack of access does not, however, explain the lower use or traditional methods and the greater exposure to family planning messaging among the military camp population. This may

For peer review only - http://bmjopen.bmj.com/site/about/guidelines.xhtml

be explained by the timing of family planning programs. According to our authors, new family planning programs were advertised in military camps recently prior to our data collection, but this was not yet matched by supply of family planning methods in military camp clinics.

This study also serves as a baseline for a programmatic initiative that started shortly after the survey in Kinshasa, which consisted of training clinical personnel at the military health center in all contraceptive methods, including Implanon NXT (highly popular in Kinshasa); training and supplying community-health workers with a non-medical profile to distribute pills, condoms and Cyclebeads at the community level; and placing billboards promoting FP near military camps that showed the father in uniform. A follow-up survey is scheduled for 2018 to assess change in FP access and use.

The strengths of this study are the rare opportunity to learn more about a neglected, but important, population for family planning; and the representative data for residents of military camps and general population of Kinshasa. However, there remain some limitations. Several measures of interest are not included in our survey instrument. Many military members and their families are likely in-migrants to Kinshasa from elsewhere in DRC; research in this setting has shown that migrants differ from Kinshasa-born residents in many characteristics related to family planning.[29] This study, unfortunately, did not measure migration status, birthplace, or duration resided in Kinshasa. Similarly, measures of access to a facility that provides family planning, such as distance to the nearest family planning clinic or pharmacy that has modern methods in stock, were not measured for the military camp population, but may differ from the general population- as suggested by our results.

Contributors: PA, PA and JB initially conceived the manuscript. PA and PA conducted the statistical analysis and wrote the first draft of the paper. HNE, GKK, JH, PK, and JB reviewed the paper before submission and provided comments and edits.

Funding statement: PMA2020 was supported by the Bill & Melinda Gates Foundation, Seattle, WA; under grant #OPP1079004. Data collection for military camps in Kinshasa was also supported by the Bill & Melinda Gates Foundation, Seattle, WA; under grant # OPP1128892.

For peer review only - http://bmjopen.bmj.com/site/about/guidelines.xhtml

Competing interests statement: the authors declare no competing interests.

Data sharing statement: All data collected under PMA2020 is made publically-available. More details on data access for PMA2020 is available here: http://www.pma2020.org/data-use.

For peer review only

Figures and Tables

Table 1: Weighted background characteristics for military and non-military women, Kinshasa 2016

	Non-military	Military
**Age groups**		
15-20	21.6%	24.5%
21-25	21.0%	20.0%
26-30	16.7%	18.4%
31-35	14.4%	15.9%
36-40	11.5%	10.5%
41-45	8.5%	5.9%
46-49	6.3%	4.8%
**Level of education**		
No education	2.1%	4.3%
Primary	18.6%	13.5%
Middle secondary school	63.6%	66.8%
Advanced secondary or higher	15.7%	15.4%
**Marital status**		
Married/partnered	47.0%	48.2%
Separated/divorced/widowed	5.3%	4.8%
Never married	47.7%	47.0%
**Wealth index**		
Quintile 1 (lowest)	15.1%	26.4%
Quintile 2	19.3%	27.0%
Quintile 3	21.3%	20.7%
Quintile 4	21.3%	15.1%
Quintile 5 (highest)	23.0%	10.8%
**Fertility and children**		
Number lifetime births	1.8	1.7
Experienced child mortality	19.7%	12.4%*
Do not want another child	18.0%	46.7%**
Last birth unintended	7.4%	26.3%**
**Family planning use**		
Contraceptive use	42.3%	17.4%**
Traditional contraceptive use	21.4%	6.6%*
Modern contraceptive use	20.9%	10.9%**
Obtained desired the FP method	92.4%	68.3%**
**Family planning knowledge**		
Heard of implants	83.3%	55.8%**
Heard of injectables	87.8%	60.9%**
Heard of condoms	94.7%	81.9%**
Heard of withdrawal	80.8%	46.9%**
Heard of rhythm	91.5%	44.6%**
**Family planning exposure**		
Visited health facility in past 12 months	55.2%	28.8%**
Heard about FP on the radio	34.7%	58.6%*

For peer review only - http://bmjopen.bmj.com/site/about/guidelines.xhtml

Read about FP in a magazine/newspaper	13.1%	50.8%**
Saw FP billboard advertisement	46.1%	89.3%**
N=	2607	514

Notes: Difference between military and non-military significant at *$p \leq 0.05$; **$p \leq 0.01$.

For peer review only

For peer review only - http://bmjopen.bmj.com/site/about/guidelines.xhtml

Table 2: Weighted regression results for differences in fertility and children between military and non-military, Kinshasa 2016

	Number of births		Child mortality		Do not want another		Last birth unintended	
	Coef	95% CI	Odds	95% CI	Odds	95% CI	Odds	95% CI
**Military**	**-0.05**	**-0.31, 0.22**	**0.53***	**0.33, 0.87**	**5.17****	**2.52, 10.62**	**5.19****	**2.31, 11.62**
Number of births	----	----	1.16**	1.05, 1.29	1.55**	1.31, 1.84	1.43**	1.23, 1.66
Age	0.33**	0.28, 0.37	1.03**	1.01, 1.06	1.02*	1.00, 1.05	0.99	0.96, 1.03
Age squared	-0.01**	-0.00, -0.00	----	----	----	----	----	----
Level of education								
No education (ref.)	----	----	----	----	----	----	----	----
Primary	0.13	-0.13, 0.38	1.05	0.50, 2.20	0.57	0.14, 1.07	1.01	0.14, 1.07
Middle secondary school	0.03	-0.28, 0.33	0.81	0.34, 1.93	0.48	0.20, 1.16	0.51	0.19, 1.32
Advanced secondary or higher	-0.37*	-0.73, -0.02	0.43	0.16, 1.18	0.39	0.14, 1.07	0.67	0.23, 1.98
Marital status								
Married/partnered (ref.)	----	----	----	----	----	----	----	----
Separated/divorced/widowed	-0.16**	-0.28, -0.05	0.72	0.40, 1.29	0.88	0.51, 1.53	2.82**	1.47, 5.42
Never married	-1.06**	-1.32, -0.80	1.03	0.67, 1.59	1.06	0.64, 1.75	2.81*	1.19, 6.66
Household wealth								
Quintile 1 (lowest, ref.)	----	----	----	----	----	----	----	----
Quintile 2	-0.13	-0.27, 0.01	0.85	0.48, 1.50	0.94	0.52, 1.63	0.54	0.20, 1.48
Quintile 3	-0.16*	-0.31, -0.01	0.88	0.57, 1.37	0.92	0.19, 1.26	0.69	0.26, 1.83
Quintile 4	-0.12	-0.29, 0.04	0.67	0.41, 1.10	0.48	0.31, 1.22	1.01	0.47, 2.16
Quintile 5 (highest)	-0.23**	-0.40, -0.06	0.77	0.44, 1.35	0.61	0.50, 1.64	0.82	0.30, 2.27
N=	3085		1795		2830		1813	

Notes: *p ≤ 0.05; **p ≤ 0.01; child mortality and last birth unintended are limited to women who have ever given birth.

For peer review only

For peer review only - http://bmjopen.bmj.com/site/about/guidelines.xhtml

Table 3: Weighted logistic regression results for differences in family planning use for military, Kinshasa 2016

	Using Contraception		Modern Contraception		Traditional Contraception		Obtained desired method	
	Odds	95% CI	Odds	95% CI	Odds	95% CI	Odds	95% CI
**Military**	**0.24****	**0.11, 0.53**	**0.25***	**0.08, 0.79**	**0.41****	**0.24, 0.71**	**0.14****	**0.03, 0.64**
Number of births	1.29**	1.17, 1.43	1.16**	1.05, 1.28	1.22**	1.13, 1.32	0.98	0.80, 1.20
Age	1.53**	1.43, 1.64	1.23**	1.14, 1.32	1.60**	1.46, 1.75	0.99	0.77, 1.28
Age quadratic	0.99**	0.99, 0.99	0.99**	0.99, 0.99	0.99**	0.99, 0.99	1.00	0.99, 1.00
Level of education								
No education (ref.)	----	----	----	----	----	----	----	----
Primary	1.68	0.75, 3.78	0.70	0.35, 1.41	3.41*	1.24, 9.33	1.59	0.25, 9.95
Middle secondary school	2.33*	1.03, 5.27	1.16	0.55, 2.45	3.42*	1.30, 9.00	1.69	0.27, 10.63
Advanced secondary or higher	3.16**	1.33, 7.53	1.55	0.65, 3.71	3.98**	1.44, 11.02	1.13	0.16, 8.00
Marital status								
Married/partnered (ref.)	----	----	----	----	----	----	----	----
Separated/divorced/widowed	0.45**	0.27, 0.75	0.41*	0.20, 0.81	0.74	0.46, 1.19	0.77	0.35, 1.71
Never married	1.52*	1.10, 2.11	1.16	0.83, 1.63	1.55**	1.12, 2.13	1.34	0.75, 2.39
Household wealth								
Quintile 1 (lowest, ref.)	----	----	----	----	----	----	----	----
Quintile 2	0.91	0.45, 1.83	1.27	0.70, 2.30	0.73	0.41, 1.30	0.46	0.13, 1.70
Quintile 3	0.82	0.41, 1.66	1.17	0.57, 2.38	0.68	0.41, 1.14	0.45	0.11, 1.77
Quintile 4	0.99	0.47, 2.11	1.29	0.60, 2.79	0.81	0.50, 1.33	0.28	0.07, 1.18
Quintile 5 (highest)	0.68	0.34, 1.34	0.96	0.48, 1.93	0.62	0.36, 1.07	0.32*	0.08, 0.92
N=	3072		3072		3072		1322	

Notes: *p ≤ .05; **p ≤ .01; analysis for obtaining desired method is limited to women currently using contraception.

For peer review only - http://bmjopen.bmj.com/site/about/guidelines.xhtml

Table 4: Weighted logistic regression results for differences in family planning knowledge among military, Kinshasa 2016

	Heard of implants		Heard of injectables		Heard of condoms		Heard of withdrawal		Heard of rhythm	
	Odds	95% CI	Odds	95% CI	Odds	95% CI	Odds	95% CI	Odds	95% CI
**Military**	**0.23****	**0.11, 0.48**	**0.19****	**0.08, 0.44**	**0.23****	**0.12, 0.47**	**0.05****	**0.02, 0.17**	**0.12****	**0.03, 0.44**
Number of births	1.06	0.95, 1.19	1.27**	1.08, 1.50	0.83*	0.72, 0.97	1.09	0.91, 1.32	1.11	0.93, 1.34
Age	1.49**	1.31, 1.70	1.31**	1.20, 1.43	1.47**	1.26, 1.72	1.27**	1.12, 1.45	1.57**	1.39, 1.77
Age squared	0.99**	0.99, 0.99	0.99**	0.99, 0.99	0.99**	0.99, 0.99	0.99**	0.99, 0.99	0.99**	0.99, 0.99
Level of education										
No education (ref.)	----	----	----	----	----	----	----	----	----	----
Primary	3.01**	1.40, 6.45	1.12	0.64, 1.95	0.36	0.07, 1.90	3.14**	1.58, 6.24	2.40**	1.30, 4.41
Middle secondary school	5.51**	2.32, 13.10	3.15**	1.60, 6.22	0.97	0.13, 7.18	7.85**	3.11, 19.85	4.29**	1.97, 9.31
Advanced secondary or higher	10.85**	4.11, 28.62	6.23**	2.34, 16.56	6.57	0.55, 78.84	27.43**	5.77, 130.44	12.80**	3.74, 43.81
Marital status										
Married/partnered (ref.)	----	----	----	----	----	----	----	----	----	----
Separated/divorced/widowed	1.18	0.56, 2.48	0.96	0.39, 2.36	0.97	0.24, 3.87	1.21	0.63, 2.34	1.44	0.57, 3.64
Never married	0.65*	0.46, 0.94	0.89	0.56, 1.41	1.27	0.58, 2.75	0.95	0.68, 1.33	0.63	0.37, 1.08
Household wealth										
Quintile 1 (lowest, ref.)	----	----	----	----	----	----	----	----	----	----
Quintile 2	0.95	0.48, 1.88	0.83	0.45, 1.54	0.75	0.27, 2.07	0.38**	0.18, 0.79	0.41*	0.18, 0.92
Quintile 3	0.79	0.39, 1.62	0.86	0.42, 1.75	0.99	0.38, 2.60	0.45*	0.20, 0.98	0.48	0.21, 1.14
Quintile 4	0.68	0.33, 1.43	0.86	0.44, 1.70	0.65	0.27, 1.53	0.77	0.31, 1.93	0.60	0.26, 1.38
Quintile 5 (highest)	0.72	0.30, 1.71	0.64	0.28, 1.48	1.06	0.37, 3.01	0.60	0.25, 1.47	0.41*	0.19, 0.93
N=	3078		3077		3080		3079		3077	

Notes: *p ≤ 0.05; **p ≤ 0.01.

For peer review only - http://bmjopen.bmj.com/site/about/guidelines.xhtml

Table 5: Weighted logistic regression results for differences in family planning exposure among military, Kinshasa 2016

	Visited health facility		Heard about FP- radio		Read about FP		FP billboard	
	Odds	95% CI	Odds	95% CI	Odds	95% CI	Odds	95% CI
**Military**	**0.28****	**0.14, 0.55**	**2.81****	**1.34, 5.92**	**7.72****	**3.13, 19.04**	**9.71****	**3.63, 25.20**
Number of births	1.07	1.00, 1.15	0.96	0.90, 1.03	0.86*	0.77, 0.97	0.97	0.90, 1.05
Age	1.11**	1.04, 1.17	1.11*	1.03, 1.20	1.12	1.00, 1.25	1.11**	1.05, 1.18
Age squared	0.99**	0.99, 0.99	1.00	0.99, 1.00	1.00	0.99, 1.00	0.99**	0.99, 1.00
Level of education								
No education (ref.)	----	----	----	----	----	----	----	----
Primary	1.21	0.60, 2.42	3.30**	1.52, 7.18	3.17	0.96, 10.50	1.22	0.60, 2.56
Middle secondary school	1.36	0.67, 2.76	6.00**	2.33, 15.48	6.05**	1.80, 20.34	1.34	0.75, 2.48
Advanced secondary or higher	1.91	0.85, 4.28	13.93**	4.88, 39.74	15.99**	4.26, 60.00	2.18*	1.12, 4.30
Marital status								
Married/partnered (ref.)	----	----	----	----	----	----	----	----
Separated/divorced/widowed	1.05	0.62, 1.76	0.89	0.57, 1.38	1.00	0.55, 1.80	0.94	0.56, 1.66
Never married	0.53**	0.42, 0.68	0.84	0.59, 1.18	0.82	0.55, 1.24	1.03	0.74, 1.42
Household wealth								
Quintile 1 (lowest, ref.)	----	----	----	----	----	----	----	----
Quintile 2	0.52**	0.33, 0.83	1.05	0.55, 2.00	0.81	0.36, 1.85	1.03	0.62, 1.69
Quintile 3	0.55*	0.35, 0.88	0.86	0.43, 1.72	0.58	0.25, 1.36	1.08	0.63, 1.94
Quintile 4	0.48**	0.31, 0.75	0.84	0.43, 1.66	0.47	0.18, 1.20	0.85	0.53, 1.49
Quintile 5 (highest)	0.59*	0.37, 0.95	0.71	0.30, 1.69	0.73	0.24, 2.20	0.91	0.50, 1.77
N=	3079		3072		3042		3072	

Notes: *p ≤ 0.05; **p ≤ 0.01.

For peer review only - http://bmjopen.bmj.com/site/about/guidelines.xhtml

References

[1] C. F. Djoko *et al.*, “High HIV Type 1 Group M pol Diversity and Low Rate of Antiretroviral Resistance Mutations Among the Uniformed Services in Kinshasa, Democratic Republic of the Congo,” *AIDS Res. Hum. Retroviruses*, vol. 27, no. 3, pp. 323–329, Mar. 2011.

[2] M. Grillo *et al.*, “Prevention Interventions for People Living with HIV in Military Settings,” *Curr. HIV Res.*, vol. 15, no. 2, pp. 90–94, 2017.

[3] S. H. Miles, “HIV in insurgency forces in sub-Saharan Africa—a personal view of policies,” *Int. J. Std Aids*, vol. 14, no. 3, pp. 174–178, 2003.

[4] U. A. Nwokoji and A. J. Ajuwon, “Knowledge of AIDS and HIV risk-related sexual behavior among Nigerian naval personnel,” *BMC Public Health*, vol. 4, no. 1, p. 24, 2004.

[5] E. A. Tarimo, T. W. Kohi, M. Bakari, and A. Kulane, “A qualitative study of perceived risk for HIV transmission among police officers in Dar es Salaam, Tanzania,” *BMC Public Health*, vol. 13, no. 1, p. 785, 2013.

[6] C. C. Nnebue, U. C. Chimah, T. O. Lawoyin, A. L. Ilika, and C. B. Duru, “Original Article: Socio-demographic Determinants of Sexual Risk Behavior among Senior Secondary Schools Students in a Military Barracks in Nigeria,” *Am. J. Public Health Res. Am. J. Public Health Res.*, vol. 3, no. 4, pp. 148–156, Jul. 2015.

[7] E. van der Ryst, G. Joubert, F. Steyn, C. Heunis, J. le Roux, and C. Williamson, “HIV/AIDS-related knowledge, attitudes and practices among South African military recruits,” *S. Afr. Med. J.*, vol. 91, no. 7, pp. 587–591, 2001.

[8] E. J. Essien, O. Mgbere, E. Monjok, E. Ekong, S. Abughosh, and M. M. Holstad, “Predictors of frequency of condom use and attitudes among sexually active female military personnel in Nigeria,” *HIVAIDS Auckl. NZ*, vol. 2, pp. 77–88, Apr. 2010.

[9] R. N. Hiremath, A. K. Yadav, S. Ghodke, and J. Yadav, “Contraceptive use and its determinants amongst armed forces personnel,” *Med. J. Armed Forces India*, 2017.

[10] A. G. Abdulrazaq, S. Kabir, N. S. Mohammad, and I. H. Suleiman, “The effect of educational intervention on family planning knowledge, attitudes, and practices among married women in a military barrack in Northern Nigeria,” *Afr. J. Reprod. Health*, vol. 18, no. 1, pp. 93–101, 2014.

[11] U. C. Chimah, T. O. Lawoyin, A. L. Ilika, and C. C. Nnebue, “Contraceptive knowledge and practice among senior secondary schools students in military barracks in Nigeria,” *Niger. J. Clin. Pract.*, vol. 19, no. 2, pp. 182–188, 2016.

[12] N. A. Hussain, T. M. Akande, G. K. Osagbemi, S. T. Olasupo, K. Y. Salawu, and E. T. Adebayo, “Perception and practice of contraception among male soldiers in Sobi barracks, Ilorin, Nigeria,” *Afr. Health Sci.*, vol. 13, no. 2, pp. 415–422, 2013.

[13] B. Dogo, “A Survey of Knowledge, Attitude and Practice of Family Planning among the Military in the Rukuba Cantonment of Kaduna,” *J. Environ. Sci.*, vol. 1, no. 2, pp. 124–132, 1998.

[14] S. Ahmed, A. A. Creanga, D. G. Gillespie, and A. O. Tsui, “Economic status, education and empowerment: implications for maternal health service utilization in developing countries,” *PloS One*, vol. 5, no. 6, p. e11190, 2010.

[15] A. A. Creanga, D. Gillespie, S. Karklins, and A. O. Tsui, “Low use of contraception among poor women in Africa: an equity issue,” *Bull. World Health Organ.*, vol. 89, no. 4, pp. 258–266, 2011.

For peer review only - http://bmjopen.bmj.com/site/about/guidelines.xhtml

[16] K. Wood and R. Jewkes, “Blood blockages and scolding nurses: barriers to adolescent contraceptive use in South Africa,” *Reprod. Health Matters*, vol. 14, no. 27, pp. 109–118, 2006.

[17] J. Hickes Lundquist and H. L. Smith, “Family formation among women in the US military: Evidence from the NLSY,” *J. Marriage Fam.*, vol. 67, no. 1, pp. 1–13, 2005.

[18] A. K. Erickson, D. A. Nelson, J. G. Shaw, P. D. Loftus, L. M. Kurina, and K. A. Shaw, “Long-Acting Reversible Contraceptive Placement Among Active-Duty U.S. Army Servicewomen,” *Obstet. Gynecol.*, vol. 129, no. 5, pp. 800–809, May 2017.

[19] V. Goyal, S. Borrero, and E. B. Schwarz, “Unintended pregnancy and contraception among active-duty servicewomen and veterans,” *Am. J. Obstet. Gynecol.*, vol. 206, no. 6, pp. 463–469, Jun. 2012.

[20] L. A. Harrington, K. A. Shaw, and J. G. Shaw, “Contraception in US servicewomen: emerging knowledge, considerations, and needs,” *Curr. Opin. Obstet. Gynecol.*, Sep. 2017.

[21] K. Holt, K. Grindlay, M. Taskier, and D. Grossman, “Unintended pregnancy and contraceptive use among women in the U.S. military: a systematic literature review,” *Mil. Med.*, vol. 176, no. 9, pp. 1056–1064, Sep. 2011.

[22] L. Enewold, L. A. Brinton, K. A. McGlynn, S. H. Zahm, J. F. Potter, and K. Zhu, “Oral contraceptive use among women in the military and the general US population,” *J. Womens Health*, vol. 19, no. 5, pp. 839–845, 2010.

[23] D. Gehlbach, “Contraceptive needs complications and new directions for research.,” *Womens Health Issues*, vol. 6, no. 6, pp. 355–8, 1996.

[24] B. R. Karney and J. S. Crown, *Families Under Stress: An Assessment of Data, Theory, and Reseach on Marriage and Divorce in the Military*. Rand Corporation, 2007.

[25] United Nations, *World Population Prospects: The 2015 Revision, Volume II: Demographic Profiles*, 2015th ed. United Nations, New York, NY.

[26] Ministère du Plan et Macro International, *Enquête Démographique et de Santé, République Démocratique du Congo 2007*. Calverton, Maryland, U.S.A.: Ministère du Plan et Macro International., 2008.

[27] Ministère du Plan et Suivi de la Mise en oeuvre de la Révolution de la Modernité (MPSMRM), Ministère de la Santé Publique (MSP) et ICF International, *Enquête Démographique et de Santé en République Démocratique du Congo 2013-2014*. Rockville, Maryland, USA: MPSMRM, MSP et ICF International., 2014.

[28] D. Filmer and L. H. Pritchett, “Estimating wealth effects without expenditure data—or tears: an application to educational enrollments in states of India,” *Demography*, vol. 38, no. 1, pp. 115–132, 2001.

[29] P. Anglewicz, J. Corker, and P. Kayembe, “The fertility of internal migrants to Kinshasa,” *Genus*, vol. 1, no. 73, pp. 1–18, 2017.

For peer review only - http://bmjopen.bmj.com/site/about/guidelines.xhtml

Female Questionnaire

Female Questionnaire

NO	QUESTIONS AND FILTERS	CODING CATEGORIES	SKIP
**IDENTIFICATION**			
A	**Are you in the correct household?**<br>**This is the picture of the front of the home taken during the Household Questionnaire.**<br>**IF NOT, RETURN TO INTERVIEW THE CORRECT HOUSEHOLD.**<br>[ODK will display the photo attached to the linked Household Questionnaire]	Yes....1<br>No....0	
B	**Your name:**<br>[Interviewer name from **Female Questionnaire**]<br>**Is this your name?**	Yes....1<br>No....0	
	**Enter your name below.**<br>*Please record your name*	Interviewer's Name	
C	**Current date and time.** [ODK will display on screen]<br>**Is this date and time correct?**	Yes....1<br>No....0	Skip to E if Yes
D	**Record the correct date and time.**	**Day** / **Month** / **Year**<br>**Hours** / **Min** / **AM/PM**	
E	**The following information is from the Female Questionnaire. Please review to make sure you are interviewing the correct respondent.**<br>[ODK will display the province, city, commune, and quartier for Kinshasa EAs and the province, district, *aire de santé*, and village for Kongo Central EAs. In addition, the Enumeration Area, Structure Number, and Household Number entered into the Household Questionnaire linked to this Female Questionnaire will be displayed.]<br>**Is the above information correct?**	Yes....1<br>No....0	
	**CHECK: You should be attempting to interview [Respondent's Name]. Is that correct?**<br>*If misspelled, select "yes" here and update the name in question "L."*<br>*If this is the wrong person, you have two options:*<br>*(1) exit and ignore changes to this form. Open the correct form.*<br>*Or*<br>*(2) find and interview the person whose name appears above.*	Yes....1<br>No....0	
F	**Is the respondent present and available to be interviewed today?**	Yes....1<br>No....0	Skip to K if No

For peer review only - http://bmjopen.bmj.com/site/about/guidelines.xhtml

Female Questionnaire

G	**How well acquainted are you with the respondent?**	Very well acquainted........................1<br>Well acquainted ....2<br>Not well acquainted..........................3<br>Not acquainted.................................4	
G2	**Has the respondent participated in a PMA2020 survey before?**	Yes ....1<br>No...0<br>Don't know ....-88<br>No response.................................-99	

**INFORMED CONSENT**<br>*Find the woman between the ages of 15-49 associated with this Female Follow Up Questionnaire. The interview must have auditory privacy. Read the following greeting:*			
Hello. My name is ________________________________ and I am working for the Kinshasa School of Public Health in collaboration with the Ministry of Health. We are conducting a survey in Kinshasa and Kongo Central that asks women about various reproductive health issues. We would very much appreciate your participation in this survey. This information will help us inform the government to better plan health services. The survey usually takes between 15 and 20 minutes to complete. Whatever information you provide will be kept strictly confidential and will not be shown to anyone other than members of our survey team. Participation in this survey is voluntary, and if we should come to any question you don't want to answer, just let me know and I will go on to the next question; or you can stop the interview at any time. However, we hope that you will participate in this survey since your views are important.<br>At this time, do you want to ask me anything about the survey?			
H	Provide a paper copy of the Consent Form to the respondent and explain it. Then, ask:<br>**May I begin the interview now?**	Yes ....1<br>No..0	Skip to K if No
	**Respondent's signature**<br>*Please ask the respondent to sign or check the box in agreement of their participation.*	GATHER SIGNATURE:<br>Check box: ☐	
I	**Interviewer's name:** [Interviewer name from Household Questionnaire]<br>*Mark your name as a witness to the consent process.*		
J	**Respondent's name**<br>[ODK will display the Respondent's name from linked Household Roster]<br>*You may correct the spelling here if it is not correct, but you must be interviewing the person whose name appears below.*		
**Section 1 – Respondent's Background, Marital Status, HH characteristics**<br>*Now I would like to ask about your background and socioeconomic conditions.*			
0	**In what month and year were you born?**<br>**The age in the household roster is [AGE].**	Month<br>Year	
1	**How old were you at your last birthday?**<br>*Must be more than 14. Must agree with FQ0.*	Age	
2	**What is the highest level of school you attended?**	Never Attended ....0<br>Primary..1<br>Secondary ....2<br>Tertiary………………………………. 3	

DRC - Round 5

For peer review only - http://bmjopen.bmj.com/site/about/guidelines.xhtml

Female Questionnaire

		No response .... -99	
3	**Are you currently married or living together with a man as if married?**<br><br>*Probe: If no, ask whether the respondent is divorced, separated, or widowed.*	No, never in union ....0<br>Yes, currently married ....1<br>Yes, living with a man ....2<br>Not currently in union:<br>Divorced / separated ....3<br>Not currently in union: Widow ....4<br>No response .... -99	Skip to 8 if No, never in union
4	**Have you been married or lived with a man only once or more than once?**	Only once ....1<br>More than once ....2<br>No response .... -99	Skip to 5b if Only once
5a	**In what month and year did you start living with your FIRST husband / partner?**<br><br>*Enter Jan 2020 for no response.*	Month [ ]<br>Year [ ]	
	[If ≤15 years old at marriage date ODK will display:] **CHECK: Based on the response you entered in FQ5a, the respondent was possibly 15 years old or younger at the time of her first marriage. Did you enter FQ5a correctly?**	Yes ....1<br>No 0	
5b	**Now I would like to ask about when you started living with your CURRENT or MOST RECENT husband / partner. In what month and year was that?**<br><br>*Enter Jan 2020 for no response.*	Month [ ]<br>Year [ ]	
	[If ≤15 years old at marriage date ODK will display:] **CHECK: Based on the response you entered in FQ5b, the respondent was possibly 15 years old or younger at the time of her first marriage. Did you enter FQ5b correctly?**	Yes ....1<br>No 0	
	**CHECK 3:** Currently married/cohabitating?	Yes ....1<br>No 0	Skip to 8 if No
6	**Does your husband / partner have other wives or does he live with other women as if married?**	Yes ....1<br>No ....0<br>Don't know................................. -88<br>No response .... -99	
7	**Is your husband / partner living with you now or is he staying elsewhere?**	Living with respondent ....1<br>Staying elsewhere ....2<br>No response .... -99	
**Section 2 – Reproduction, Pregnancy & Fertility Preferences**<br>*Now I would like to ask about all the births you have had during your life.*			
8a	**How many times have you given birth?**<br><br>*Enter -99 for no response. 0 is a possible answer.*	Number [ ]	Skip to 13 if 0
	**Were all of those live births?**<br><br>*If no, go back and change FQ8 to record only live birth events.*	Yes ....1<br>No 0	Skip to 9 if 8 was 1
8b	**How many sons and daughters have you given birth to and who were born alive?**		

For peer review only - http://bmjopen.bmj.com/site/about/guidelines.xhtml

Female Questionnaire

		Number [ ]	
8c	**Have you ever given birth to a boy or girl who was born alive but later died?**<br>*IF NO, PROBE: Any baby who cried or showed signs of life but did not survive?*	Yes ....1<br>No 0	Skip to 8e if No
8d	**How many have died?**<br>*Enter -88 for do not know and -99 for No response.*<br>*Change FQ8c to 'No' if zero deaths.*	Number [ ]	
	**READ THIS CHECK OUT LOUD: Just to make sure I have this right: you had a total of ___ birth(s) during your life, resulting in ____ son(s) or daughter(s) born alive.**<br>**Is that correct?**	Yes ....1<br>No 0	If no, go back and probe to correct 8a-c.
8e	**When was your FIRST birth?**<br>*Please record the date of the first live birth. Date should be found by calculating forward or backward from memorable events if needed. Enter Jan 2020 for no response.*	Month [ ]<br>Year [ ]	
9	**When was your MOST RECENT birth?**<br>*Please record the date of the MOST RECENT live birth. The date should be found by calculating backwards from memorable events if needed.*<br>*Enter Jan 2020 for no response.*	Month [ ]<br>Year [ ]	Skip to 11 if not in last year and/or Q8 is 1
10	**When did you give birth before the most recent one?**<br>*Please record the date of the birth before the last. The date should be found by calculating backwards from memorable events if needed.*<br>*Enter Jan 2020 for no response.*	Month [ ]<br>Year [ ]	
11	**Is your last baby / child still alive?**	Yes ....1<br>No ....0<br>Don't know.... -88<br>No response .... -99	Skip to 13 if Yes
12	**When did your last baby / child die?**<br>*Please record the date of the child's death.*<br>*The date should be found by calculating backwards from memorable events if needed.*<br>*Enter Jan 2020 for no response.*	Month [ ]<br>Year [ ]	
13	**When did your last menstrual period start?**<br>*If you select days, weeks, months or years, you will enter a number for x on the next screen.*<br>*Enter 0 days for today, not 0 weeks/months/years.*	______ days ago<br>______ weeks ago<br>_______ months ago<br>_______ years ago	

For peer review only - http://bmjopen.bmj.com/site/about/guidelines.xhtml

		Menopausal / Hysterectomy............5<br>Before last birth ....6<br>Never menstruated...........................7<br>No response .... -99	
14	**Are you pregnant now?**	Yes ....1<br>No...0<br>Unsure...2<br>No response .... -99	Skip to 16 if No or Unsure
15	**How many months pregnant are you?**<br>**The most recent birth was: [Date of most recent birth]**<br>*Please record the number of completed months.*<br>*Enter -88 for do not know, -99 for no response.*	Number of months [ ]	
	**CHECK 14:** Currently pregnant?	Yes ....1<br>No...0	16a if no 16b if yes
16a	**Now I have some questions about the future. Would you like to have a/another child or would you prefer not to have any / any more children?**	Have a/another child ....1<br>No more..2<br>Says she can't get pregnant............3<br>Undecided / Don't know .... -88<br>No response .... -99	Skip to 17a if 1 and 18 for all other
16b	**Now I have some questions about the future. After the child you are expecting now, would you like to have another child, or would you prefer not to have any more children?**	Have a/another child ....1<br>No more/prefer no children ....2<br>Says she can't get pregnant............3<br>Undecided / Don't know .... -88<br>No response .... -99	Skip to 17b if 1 and 18 for all other
17a	**How long would you like to wait from now before the birth of a/another child?**<br>*If you select months or years, you will enter a number for x on the next screen.*<br>*Select "Years" if more than 36 months.*	Months [ ]<br>Years [ ]<br>Soon / now ....1<br>Says she can't get pregnant............2<br>Other ....3<br>Don't know.................................. -88<br>No response .... -99	
17b	**After the birth of the child you are expecting now, how long would you like to wait before the birth of another child?**<br>*If you select months or years, you will enter a number for x on the next screen.*<br>*Select "Years" if more than 36 months.*	Months [ ]<br>Years [ ]<br>Soon / now ....1<br>Says she can't get pregnant............2<br>Other ....3<br>Don't know.................................. -88<br>No response .... -99	
	**CHECK 8:** Number of births	Number of births [ ]	Skip to 19 if 0 births and 14:

For peer review only - http://bmjopen.bmj.com/site/about/guidelines.xhtml

Female Questionnaire

	**CHECK 14**: Currently pregnant?	Yes………………………………… 1<br>No……………………………….…0	No. Skip to 18a if 14: no and 18b if 14: yes
18a	**Now I would like to ask a question about your last birth.**<br>**At the time you became pregnant, did you want to become pregnant then, did you want to wait until later, or did you not want to have any / any more children at all?**	Then ....1<br>Later ....2<br>Not at all ....3<br>No response .... -99	
18b	**Now I would like to ask a question about your current pregnancy.**<br>**At the time you became pregnant, did you want to become pregnant then, did you want to wait until later, or did you not want to have any / any more children at all?**	Then ....1<br>Later ....2<br>Not at all ....3<br>No response .... -99	

<u>Section 3A – Contraception</u>

Now I would like to talk about family planning - the various ways or methods that a couple can use to delay or avoid a pregnancy.

An image will appear on the screen for some methods. If the respondent says that she has not heard of the method or if she hesitates to answer, read the probe aloud and show her the image, if available.

19	**Have you ever heard of female sterilization?**<br>PROBE: Women can have an operation to avoid having any more children.<br>[NO IMAGE]	Yes... 1<br>No .... 0<br>No response……………………..-99	
19	**Have you ever heard of male sterilization?**<br>PROBE: Men can have an operation to avoid having any more children.<br>[NO IMAGE]	Yes... 1<br>No .... 0<br>No response……………………..-99	
19	**Have you ever heard of the contraceptive implant?**<br>PROBE: Women can have one or several small rods placed in their upper arm by a doctor or nurse, which can prevent pregnancy for one or more years.<br>[IMAGE OF METHOD WILL APPEAR ON SCREEN]	Yes... 1<br>No .... 0<br>No response……………………..-99	
19	**Have you ever heard of the IUD?**<br>PROBE: Women can have a loop or coil placed inside them by a doctor or a nurse.<br>[IMAGE OF METHOD WILL APPEAR ON SCREEN]	Yes... 1<br>No .... 0<br>No response……………………..-99	
19	**Have you ever heard of injectables?**	Yes... 1<br>No 0	

For peer review only - http://bmjopen.bmj.com/site/about/guidelines.xhtml

Female Questionnaire

	PROBE: Women can have an injection by a health provider that stops them from becoming pregnant for one or more months.<br><br>[IMAGE OF SAYANA PRESS AND DEPO PROVERA WILL APPEAR ON SCREEN]	No response……………………..-99	
19	**Have you ever heard of the (birth control) pill?**<br><br>PROBE: Women can take a pill every day to avoid becoming pregnant.<br><br>[IMAGE OF METHOD WILL APPEAR ON SCREEN]	Yes.. 1<br>No .... 0<br>No response……………………..-99	
19	**Have you ever heard of emergency contraception?**<br><br>PROBE: As an emergency measure after unprotected sexual intercourse women can take special pills at any time within five days to prevent pregnancy.<br><br>[NO IMAGE]	Yes.. 1<br>No .... 0<br>No response……………………..-99	
19	**Have you ever heard of condoms?**<br><br>PROBE: Men can put a rubber sheath on their penis before sexual intercourse.<br><br>[IMAGE OF METHOD WILL APPEAR ON SCREEN]	Yes.. 1<br>No .... 0<br>No response……………………..-99	
19	**Have you ever heard of female condoms?**<br><br>PROBE: Women can put a sheath in their vagina before sexual intercourse.<br><br>[IMAGE OF METHOD WILL APPEAR ON SCREEN]	Yes.. 1<br>No .... 0<br>No response……………………..-99	
19	**Have you ever heard of the diaphragm?**<br><br>PROBE: Women can place a thin flexible disk in their vagina before sexual intercourse.<br><br>[IMAGE OF METHOD WILL APPEAR ON SCREEN]	Yes.. 1<br>No .... 0<br>No response……………………..-99	
19	**Have you ever heard of foam or jelly as a contraceptive method?**<br><br>PROBE: Women can place a suppository, jelly, or cream in their vagina before sexual intercourse to prevent pregnancy.	Yes.. 1<br>No .... 0<br>No response……………………..-99	

For peer review only - http://bmjopen.bmj.com/site/about/guidelines.xhtml

Female Questionnaire

	[IMAGE OF METHOD WILL APPEAR ON SCREEN]		
19	**Have you ever heard of the standard days method or Cycle Beads?**<br><br>PROBE: A Woman can use a string of colored beads to know the days she can get pregnant. On the days she can get pregnant, she and her partner use a condom or do not have sexual intercourse.<br><br>[IMAGE OF METHOD WILL APPEAR ON SCREEN]	Yes.. 1<br>No .... 0<br>No response……………………….-99	
19	**Have you ever heard of the Lactational Amenorrhea Method or LAM?**<br><br>[NO DESCRIPTION; NO IMAGE]	Yes.. 1<br>No .... 0<br>No response………………………..-99	
19	**Have you ever heard of the rhythm method?**<br><br>PROBE: Women can avoid pregnancy by not having sexual intercourse on the days of the month they think they can get pregnant.<br><br>[NO IMAGE]	Yes.. 1<br>No .... 0<br>No response………………………..-99	
19	**Have you ever heard of the withdrawal method?**<br><br>PROBE: Men can be careful and pull out before climax.<br><br>[NO IMAGE]	Yes.. 1<br>No .... 0<br>No response………………………..-99	
19	**Have you ever heard of any other ways or methods that women or men can use to avoid pregnancy?**	Yes.. 1<br>No .... 0<br>No response………………………..-99	
	**CHECK 14:** Currently pregnant?	Yes.. 1<br>No 0	Skip to 23 if yes
20	**Are you or your partner currently doing something or using any method to delay or avoid getting pregnant?**	Yes.. 1<br>No 0	Skip to 23 if No

For peer review only - http://bmjopen.bmj.com/site/about/guidelines.xhtml

Female Questionnaire

21	**Which method or methods are you using?**<br>**Probe: Anything else?**<br>*Select all methods mentioned. Be sure to scroll to bottom to see all choices.*	Female sterilization........................ 1<br>Male sterilization .... 2<br>Implant .... 3<br>IUD .... 4<br>Injectable .... 5<br>Pill .... 7<br>Emergency Contraception .... 8<br>Male Condom .... 9<br>Female Condom .... 10<br>Diaphragm .... 11<br>Foam/Jelly .... 12<br>Std. Days/Cycle beads .... 13<br>LAM .... 14<br>Rhythm method .... 30<br>Withdrawal .... 31<br>Other traditional method .... 39<br>No response .... -99	Skip based on most effective method only<br>If injectable is selected, skip to FQ21a<br>If LAM is selected but not injectables, skip to 21b<br>If FS or MS was selected without injectables or LAM, skip to 22<br>If LAM, FS, MS, and injectables are not selected, skip to 26b	
21a	**PROBE:** Was the injection administered via syringe or small needle?<br>*Show the image to the respondent.*<br>[IMAGES OF BOTH INJECTION SYSTEMS WILL APPEAR ON SCREEN]	Syringe .... 1<br>Small needle (Sayana Press).......... 2<br>Both .... 3<br>No response………………………..-99	Skip to CHECK FQ21	
21b	**Are you breastfeeding to delay or avoid becoming pregnant?**	Yes.. 1<br>No .... 0<br>No Response……………………… -99		
	**CHECK FQ21:** Using Female Sterilization and/or Male Sterilization?	Y / N<br>Female Sterilization........................ 1 / 0<br>Male Sterilization .... 1 / 0<br>None of the above .... -77	Skip to 26b if -77	
22	**Did the provider tell you or your partner that this method was permanent?**	Yes.. 1<br>No .... 0<br>No response………………………..-99	Skip to 26b	
23	**Do you know of a place where you can obtain a method of family planning?**	Yes.. 1<br>No .... 0<br>No response………………………..-99		
	**CHECK 14:** Currently pregnant?	Yes.. 1<br>No 0	Skip to 24b if yes	
24a	**You said that you are not currently using a contraceptive method. Do you think you will use a contraceptive method to delay or avoid getting pregnant at any time in the future?**	Yes.. 1<br>No .... 0<br>No response………………………..-99		

For peer review only - http://bmjopen.bmj.com/site/about/guidelines.xhtml

Female Questionnaire

24b	**Do you think you will use a contraceptive method to delay or avoid getting pregnant at any time in the future?**	Yes ... 1<br>No ... 0<br>No response ... -99	
25	**In the last 12 months, have you ever done something or used a method to delay or avoid getting pregnant?**	Yes ... 1<br>No ... 0<br>No response ... -99	Skip to 41 if No
26	**Which method did you use most recently?**<br>**Probe: Anything else?**<br>*Select most effective method (highest method on list). Scroll to bottom to see all choices.*	Implant ... 3<br>IUD ... 4<br>Injectable ... 5<br>Pill ... 7<br>Emergency Contraception ... 8<br>Male Condom ... 9<br>Female Condom ... 10<br>Diaphragm ... 11<br>Foam/Jelly ... 12<br>Std. Days/Cycle beads ... 13<br>LAM ... 14<br>Rhythm method ... 30<br>Withdrawal ... 31<br>Other traditional method ... 39<br>No response ... -99	Skip to FQ26b unless injectables selected
26a	**PROBE:** Was the injection administered via syringe or small needle?<br>*Show the image to the respondent.*<br>[IMAGES OF BOTH INJECTION SYSTEMS WILL APPEAR ON SCREEN]	Syringe ... 1<br>Small needle (Sayana Press) ... 2<br>Both ... 3<br>No response ... -99	
26b	**Before you started using [MOST RECENT / CURRENT METHOD], did you talk with your husband / partner about using a contraceptive method?**	Yes ... 1<br>No ... 0<br>Don't know ... -88<br>No response ... -99	
27	**When did you begin using your [MOST RECENT / CURRENT METHOD]?**<br>*Calculate backwards from memorable events if needed.*<br>**Age at first use: [Age from FQ20]**<br>**Most Recent Birth: [mm-yyyy]**<br>**Current Marriage: [mm-yyyy]**<br>*Must be at least the ages she started using a contraceptive method (FQ20).*<br>*Must be before today. Respondent must be at least 10 years old.*<br>*Enter Jan 2020 for no response.*	Month [ ]<br>Year [ ]	

For peer review only

For peer review only - http://bmjopen.bmj.com/site/about/guidelines.xhtml

Female Questionnaire

	**CHECK 20:** Currently using contraceptives?	Yes .... 1<br>No 0	Skip to 30 if Yes
28	**When did you stop using your [MOST RECENT METHOD]?**<br>*Please record the date.*<br>*The date should be found by calculating backwards from memorable events if needed. Must be after FQ27.*<br>*Enter Jan 2020 for no response.*	Month [ ]<br>Year [ ]	
29	**Why did you stop using your (MOST RECENT METHOD)?**	Infrequent sex / husband away........ 1<br>Became pregnant while using .... 2<br>Wanted to become pregnant .... 3<br>Husband / partner disapproved .... 4<br>Wanted more effective method........ 5<br>No method available........................ 6<br>Health concerns............................... 7<br>Fear of side effects .... 8<br>Lack of access / too far.................... 9<br>Costs too much.............................. 10<br>Inconvenient to use .... 11<br>Fatalistic... 12<br>Difficult to get pregnant / menopausal .... 13<br>Interferes with body's processes ... 14<br>Other .... 15<br>Don't know ....-88<br>No response ....-99	
30	**You first started using [CURRENT/MOST RECENT METHOD] in [DATE FROM FQ27]. Where did you get it at that time?**<br>*Scroll to bottom to see all choices.*	**PUBLIC SECTOR:**<br>**NATIONAL HOSPITAL..11**<br>**FAMILY PLANNING CLINIC............................... 12**<br>**HEALTH CENTRE/POSTE DE SANTE...................13**<br>**MATERNITY..14**<br>**COMMUNITY HEALTH VOLUNTEER..................15**<br>**REGIONAL HOSPITAL..16**<br><br>**PRIVATE MEDICAL SECTOR:**<br>**PRIVATE HOSPITAL/CLINIC............................... 21**<br>**PHARMACY ....22**<br>**NGO...23**<br>**PRIVATE HEALTH CENTER..................................24**<br>**PRIVATE PRACTICE ....25**<br>**PRIVATE DOCTOR ....26**<br>**MOBILE NURSE..27**<br>**Community health worker (ASC)........................28**<br>**Community-based medical student..29**<br><br>**OTHER SOURCE:**<br>**BOUTIQUE...31**<br>**RELIGIOUS INSTITUTION...................................32**<br>**FRIEND/RELATIVE ....33**<br>**BAR/NIGHT CLUB..34**<br>**LIGABLO/KIOSK..35**<br>**CHAYEUR..36**<br>**OTHER .... 37**<br>**NO RESPONSE.. -99**	

For peer review only - http://bmjopen.bmj.com/site/about/guidelines.xhtml

Female Questionnaire

31	**When you obtained your [MOST RECENT / CURRENT METHOD], were you told by the provider about side effects or problems you might have with a method to delay or avoid getting pregnant?**	Yes.......... 1<br>No .... 0<br>No response .... -99	Skip to 33 if No
32	**Were you told what to do if you experienced side effects or problems?**	Yes.......... 1<br>No .... 0<br>No response .... -99	
33	**At that time, were you told by the family planning provider about methods of family planning other than the [MOST RECENT/CURRENT METHOD] that you could use?**	Yes.......... 1<br>No .... 0<br>No response .... -99	
34	**During that visit, did you obtain the method you wanted to delay or avoid getting pregnant?**	Yes.......... 1<br>No .... 0<br>No response .... -99	Skip to 36 if yes
35	**Why didn't you obtain the method you wanted?**	Method out of stock that day .... 1<br>Method not available at all.......... 2<br>Provider not trained to provide the method.......... 3<br>Provider recommended a different method.......... 4<br>Not eligible for method.......... 5<br>Decided not to adopt a method .... 6<br>Too costly .... 7<br>Other.......... 8<br>No response .... -99	
36	**During that visit, who made the final decision about what method you got?**	You alone.......... 1<br>Provider .... 2<br>Partner .... 3<br>You and provider .... 4<br>You and partner.......... 5<br>Other.......... 6<br>No response .... -99	

For peer review only

For peer review only - http://bmjopen.bmj.com/site/about/guidelines.xhtml

Female Questionnaire

	**CHECK 30: You first started using [CURRENT/MOST RECENT METHOD] in [DATE FROM FQ27]. Where did you get it at that time?**	**PUBLIC SECTOR:**<br>**NATIONAL HOSPITAL……………………………………11**<br>**FAMILY PLANNING CLINIC………………………… 12**<br>**HEALTH CENTRE/POSTE DE SANTE………………13**<br>**MATERNITY……………………………………………14**<br>**COMMUNITY HEALTH VOLUNTEER………………15**<br>**REGIONAL HOSPITAL…………………………………16**<br><br>**PRIVATE MEDICAL SECTOR:**<br>**PRIVATE HOSPITAL/CLINIC………………………… 21**<br>**PHARMACY ……………………………………………22**<br>**NGO……………………………………………………23**<br>**PRIVATE HEALTH CENTER……………………………24**<br>**PRIVATE PRACTICE ……………………………………25**<br>**PRIVATE DOCTOR ………………………………………26**<br>**MOBILE NURSE…………………………………………27**<br>**Community health worker (ASC) ………………28**<br>**Community-based medical student……………………………………………29**<br><br>**OTHER SOURCE:**<br>**BOUTIQUE………………………………………………31**<br>**RELIGIOUS INSTITUTION……………………………32**<br>**FRIEND/RELATIVE ……………………………………33**<br>**BAR/NIGHT CLUB………………………………………34**<br>**LIGABLO/KIOSK…………………………………………35**<br>**CHAYEUR…………………………………………………36**<br>**OTHER ………………………………………………… 37**<br>**NO RESPONSE…………………………………………… -99**	Skip to 41 if 30 is 33 OR 37
37	**Would you return to this provider?**<br><br>**Provider: [Type of Provider from FQ30]**	Yes…………………………………………… 1<br>No …………………………………………… 0<br>No response ………………………… -99	
38	**Would you refer your relative or friend to this provider / facility?**	Yes…………………………………………… 1<br>No …………………………………………… 0<br>No response ………………………… -99	
39	**In the last 12 months, have you paid any fees for family planning services (including the most current method)?**	Yes…………………………………………… 1<br>No …………………………………………… 0	Skip to 41 if No
40	**How much did you pay?**<br>*Enter all prices in Congolese Francs. Enter -88 if respondent does not know, -99 for no response.*	Fee [ ]	

For peer review only - http://bmjopen.bmj.com/site/about/guidelines.xhtml

Female Questionnaire

40a	**Where did you obtain [CURRENT METHOD] the last time?**<br><br>***Scroll to bottom to see all choices.***	**PUBLIC SECTOR:**<br>**NATIONAL HOSPITAL……………………………………11**<br>**FAMILY PLANNING CLINIC…………………………… 12**<br>**HEALTH CENTRE/POSTE DE SANTE…………………13**<br>**MATERNITE………………………………………………14**<br>**COMMUNITY HEALTH VOLUNTEER………………15**<br>**REGIONAL HOSPITAL…………………………………….16**<br><br>**PRIVATE MEDICAL SECTOR:**<br>**PRIVATE HOSPITAL/CLINIC…………………………. 21**<br>**PHARMACY ……………………………………………22**<br>**NGO……………………………………………………23**<br>**PRIVATE HEALTH CENTER……………………………24**<br>**PRIVATE PRACTICE ……………………………………25**<br>**PRIVATE DOCTOR ………………………………………26**<br>**MOBILE NURSE……………………………………………27**<br>**FIELDWORKER…………………………………………28**<br>**Community health worker (ASC)………………28**<br>**Community-based medical student……………………………………29**<br><br>**OTHER SOURCE:**<br>**BOUTIQUE………………………………………………31**<br>**RELIGIOUS INSTITUTION………………………………32**<br>**FRIEND/RELATIVE ………………………………………33**<br>**BAR/NIGHT CLUB…………………………………………34**<br>**LIGABLO/KIOSK…………………………………………35**<br>**CHAYEUR…………………………………………………36**<br>**OTHER ………………………………………………… 37**<br>**NO RESPONSE…………………………………………. -99**	
41	**Have you ever done anything or tried in any way to delay or avoid getting pregnant?**	Yes ………………………………1<br>No…………………………………0<br>No response ………………… -99	Skip to 43 if No
41b	**How old were you when you first used a method to delay or avoid getting pregnant?**<br>**The respondent said she was [age from FQ1] years old at her last birthday.**<br>*Enter the age in years.*<br>*Enter -88 if respondent does not know.*<br>*Enter -99 if there is no response.*<br>*Cannot be younger than 9.*	Age ☐	
41c	**How many living children did you have at that time, if any?**<br>**Note: the respondent said that she gave birth [number of live births] times in FQ8.**<br>*Enter -99 for no response*	Number ☐	

For peer review only - http://bmjopen.bmj.com/site/about/guidelines.xhtml

Female Questionnaire

42	**Which method did you first use to delay or avoid getting pregnant?**<br>*Do not read the method choices. Be sure to scroll to bottom to see all choices.*	Female sterilization .... 1<br>Male sterilization .... 2<br>Implant .... 3<br>IUD .... 4<br>Injectable .... 5<br>Pill .... 7<br>Emergency Contraception .... 8<br>Male Condom .... 9<br>Female Condom .... 10<br>Diaphragm .... 11<br>Foam/Jelly .... 12<br>Std. Days/Cycle beads .... 13<br>LAM .... 14<br>Rhythm method .... 30<br>Withdrawal .... 31<br>Other traditional method............. 39<br>No response .... -99	If Injectables is selected, go to 42a
42a	**PROBE:** Was the injection administered via syringe or small needle?<br>*Show the image to the respondent.*<br>[IMAGES OF BOTH INJECTION SYSTEMS WILL APPEAR ON SCREEN]	Syringe .... 1<br>Small needle (Sayana Press) .... 2<br>Both .... 3<br>No response……………………..-99	
	**CHECK 16:** Desire for future child?<br><br>**CHECK 17**: 2 or more years before next child?<br><br>**CHECK 20:** Currently using contraceptive method?	Have a/another child ....1<br>No more/none.................................2<br>Says she can't get pregnant............3<br>Undecided / Don't know .... -88<br>No more/none.................................1<br>Less than 2 years ....2<br>2 or more years ....3<br>Yes, using contraceptive ....1<br>No, not using contraceptive.............0	Ask 43 to non-users (current or ever) who do not want a/another child or not before 2 years.
43	**You said that you do not want any / anymore children and that you are not using a method to avoid pregnancy.**<br>**Can you tell me the reason why you are not using a method to prevent pregnancy?**<br>**PROBE: Any other reason?**<br>*RECORD ALL REASONS MENTIONED.*<br>*Cannot select "Do Not Know" or "No response" with other options.*<br>*Cannot select "Not married" if FQ3 is "Yes, currently married".*<br>*Scroll to the bottom to see all choices.*	Not married.......................................1<br>Infrequent sex / husband away ....2<br>Menopausal/Hysterectomy...............3<br>Subfecund / infecund ....4<br>Not menstruated since last birth.......5<br>Breastfeeding ....6<br>Husband away for many days..........7<br>Up to God / fatalistic........................8<br>Respondent opposed ....9<br>Husband / partner opposed............10<br>Others opposed..............................11<br>Religious prohibition ....12<br>Knows no method ....13<br>Knows no source............................14<br>Fear of side effects.........................15<br>Health concerns ....16<br>Lack of access / too far ....17<br>Costs too much18Preferred method n<br>No method available ....20<br>Inconvenient to use 21	

For peer review only - http://bmjopen.bmj.com/site/about/guidelines.xhtml

Female Questionnaire

		Interferes with body's processes....22<br>Takes too much time away from regular duties / too busy to go get one................23<br>Intention to use one but did not get a chance to go to the clinic yet.........24<br>Mother-in-law opposed..................25<br>Other ....36<br>Don't know.................................. -88<br>No response .... -99	
44	**In the last 12 months, were you visited by a community health worker who talked to you about family planning?**	Yes ....1<br>No...0<br>No response .... -99	
44a	**In the last 12 months, did you participate in a group talk at the community level about family planning?**	Yes.. 1<br>No .... 0<br>No response ....-99	
45	**In the last 12 months, have you visited a health facility for care for yourself or your children?**<br>*For any health services*	Yes.. 1<br>No .... 0<br>No response ....-99	Skip to 47 if no
46	**Did any staff member at the health facility speak to you about family planning methods?**	Yes.. 1<br>No .... 0<br>No response ....-99	
47	**In the last few months have you:**	Yes / No / NR	
	**Heard about family planning on the radio?**...........................	1 / 0 / -99	
	**Seen anything about family planning on the television?**...........................	1 / 0 / -99	
	**Read about family planning in a newspaper or magazine?**	1 / 0 / -99	
47b	**Have you seen this image before?**<br>***Show the logo to the respondent.***	Yes.. 1<br>No .... 0<br>Don't know/Don't recall.................-88<br>No response ....-99	Skip to 47d if 0, -88, or -99
47b1	**Where did you see this image?**<br>**PROBE: Anywhere else?**<br>*Select all that apply.*	In a health center .... 1<br>In the street .... 2<br>In a pharmacy .... 3<br>On a billboard ……………………….4<br>On the TV ……………………………5<br>On a leaflet …………………………6<br>On a community heath agent's vest…7	

For peer review only

For peer review only - http://bmjopen.bmj.com/site/about/guidelines.xhtml

Female Questionnaire

		Other .... 8<br>Don't know/Don't recall .... -88<br>No response .... -99	
47c	**What is this image trying to say?**<br><br>***Select all that apply.***	Family planning .... 1<br>Birth spacing .... 2<br>Reproductive health .... 3<br>The family unit .... 4<br>Other .... 5<br>Don't know .... -88<br>No response .... -99	
47d	**Have you seen the billboard that states "How many children do you want?", and shows the FP logo ?**<br><br>**Show a photo of the billboard to the respondent.**<br>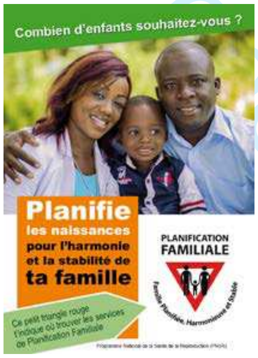 	Yes .... 1<br>No .... 0<br>Don't know/Don't recall .... -88<br>No response .... -99	Skip to 48 if 0, -88 or -99
47e	**What is this image trying to say?**<br><br>***Select all that apply.***	Family planning .... 1<br>Birth spacing .... 2<br>Reproductive health .... 3<br>The family unit .... 4<br>Other .... 5<br>Don't know / Don't recall .... -88<br>No response .... -99	
	**CHECK FOR THE PRESENCE OF OTHERS. BEFORE CONTINUING, MAKE EVERY EFFORT TO ENSURE PRIVACY.**<br>***Verbally prepare the respondent for sexual activity questions.***		
48	**How old were you when you first had sexual intercourse?**<br><br>***The respondent said she was [age from FQ1] years old at her last birthday.***<br><br>***[She has had x live births.]***<br><br>*Enter the age in years.*<br><br>*Enter -77 if she never had sex.*<br>*Enter -88 if respondent does not know.*<br>*Enter -99 for no response.*	Age ____	Skip to MM_1 if -77

DRC - Round 5

For peer review only - http://bmjopen.bmj.com/site/about/guidelines.xhtml

Female Questionnaire

	[If age at first sex <10 years:]<br>**You have entered that the respondent was X years old when she first had sexual intercourse. Is this what she said?**<br>*Go back and correct FQ48 if it is not correct.*	Yes ....1<br>No..0	
49	**When was the last time you had sexual intercourse?**<br>*If less than 12 months ago, answer must be recorded in months, weeks, or days.*<br>*Enter 0 days for today.*<br>*You will enter a number for X on the next screen.*	______ days ago<br>______ weeks ago<br>_______ months ago<br>_______ years ago	

For peer review only - http://bmjopen.bmj.com/site/about/guidelines.xhtml

Female Questionnaire

Section 4 – Exposure to Mass Media<br>*Now I would like to talk in more detail about your exposure to family planning information through mass media.*			
MM_1	**Did you watch the television show 'Libala Ya Bosembo' within the past 6 months?**	Yes ....1<br>No ....0<br>No response .... -99	Skip to MM_6 if 0 or -99
MM_2	At the end of each libala ya bosembo show a message is given. The last time you watched the show, what messages had the greatest impression on you? ?<br>**PROBE: What else?**<br>***Select all that apply.***	Couple communication....1<br>Harmony in the family ....2<br>Family planning methods ....3<br>Antenatal care ....4<br>Care for mother and child's health in the postpartum period ....5<br>Amenorrheic period after pregnancy 6<br>Birth spacing ....7<br>Breast feeding ....8<br>Other ....9<br>Don't know / Don't recall .... -88<br>No response .... -99	
MM_3	**Did they talk about using family planning methods during the show?**	Yes ....1<br>No ....0<br>Don't know / Don't recall .... -88<br>No response .... -99	Skip to MM_6 if 0, -88 or -99
MM_4	**Did you speak with someone about the family planning messages from the television show 'Libala Ya Bosembo?'**	Yes ....1<br>No ....0<br>Don't know / Don't recall .... -88<br>No response .... -99	Skip to MM_6 if 0, -88 or -99
MM_5	**With whom did you speak?**<br>**PROBE: Anyone else?**<br>***Select all that apply.***	Medical provider ....1<br>Spouse ....2<br>Other relatives ....3<br>Friends/neighbors ....4<br>Pharmacist/pharmacist's aid ....5<br>Outreach worker ....6<br>People at a seminar/community meeting ....7<br>Other ....8<br>Don't know / Don't recall .... -88<br>No response .... -99	
MM_6	**Have you ever watched the mini-television show called "Elengi"?**	Yes ....1<br>No ....0<br>No response .... -99	Skip to MM_8 if 0 or -99
MM_7	**Last time you watched the mini-television show Elengi, what was it about?**<br>**PROBE: What else?**<br>***Select all that apply.***	Couple communication....1<br>Harmony in the family ....2<br>Family planning methods ....3<br>Antenatal care ....4<br>Care for mother and child's health in the postpartum period ....5<br>Amenorrheic period after pregnancy 6<br>Birth spacing ....7<br>Breast feeding 8	

For peer review only - http://bmjopen.bmj.com/site/about/guidelines.xhtml

Female Questionnaire

		Other ...9<br>Don't know / don't recall ... -88<br>No response ... -99	
MM_8	**Have you ever called the telephone hotline '3-2-1'?**	Yes ...1<br>No...0<br>No response ... -99	Skip to SE_1 if 0 or -99
MM_9	The last time you called the hotline 3-2-1, what did you want information about?<br><br>**PROBE: Anything else?**<br><br>***Select all that apply.***	Family planning service locations ...1<br>Information about family planning methods...2<br>Side-effects of FP methods ...3<br>Post-abortion care ...4<br>Post intimate partner violence care..5<br>HIV/AIDS ...6<br>Health in general ...7<br>Other ...8<br>Don't know... -88<br>No response ... -99	

	**Section 5 – Perceived self-efficacy in contraceptive use**<br><br>*Now I would like to talk about your level of confidence in your ability to access and use family planning methods. If you are not currently married or have a regular partner, try to imagine how you would respond if you were currently in union.*					
	How confident are you that:	*Very Confident*	*Confident*	*Somewhat confident*	*Not very confident*	*Not at all confident*
SE_1	You can start a conversation with your husband/spouse/partner about family planning/birth spacing?	5	4	3	2	1
SE_2	You can convince your husband/spouse/partner to use a method of family planning/birth spacing?	5	4	3	2	1
SE_3	You can go to a site where methods of family planning / birth spacing are available if you decide to use one?	5	4	3	2	1
SE_4	You can get a method of family planning / birth spacing if you decide to use one?	5	4	3	2	1
SE_5	You could use a method of family planning / birth spacing, even if your husband/partner does not want to?	5	4	3	2	1
SE_6	You can use a method of family planning / birth spacing, even if none of your friends or neighbors use one?	5	4	3	2	1
SE_7	You can use a method of family / child spacing planning, even if your religious leader does not think you should use it?	5	4	3	2	1
SE_8	You will continue to use a method of family planning / birth spacing, even if you experience side effects?	5	4	3	2	1

For peer review only - http://bmjopen.bmj.com/site/about/guidelines.xhtml
For peer review only

Female Questionnaire

	**Section 6 – Diarrheal Disease Among Children**<br>*Now I would like to ask about your water practices.*	
50	**How many children under age 5, if any, live in this household for which you are the primary caregiver?**	Number [ ]
	Starting with the youngest child, I'd like to ask you some questions.<br><br>**[ODK Will repeat the FQ51-FQ53 each child under age 5.]**	
51	In what month and year was this child born?<br>**ENTER JAN 2020 FOR NO RESPONSE.**	Month [ ]<br>Year [ ]
52	The last time this child passed stools, what was done to dispose of the stools?<br>**FOR ALL FECES, NORMAL OR DIARRHEA.**	Yes / No
	Children use a latrine / toilet	1 / 0
	Leave waste where it is	1 / 0
	Bury waste in field / yard	1 / 0
	Dispose of waste in latrine / toilet	1 / 0
	Dispose of waste with rubbish / garbage	1 / 0
	Dispose of waste with waste water	1 / 0
	Use it as manure	1 / 0
	Burn it	1 / 0
	No response	-99
53	In the past 7 days, has this child had diarrhea?<br><br>***Diarrhea is determined as perceived by mother/ caretaker. If the respondent is not sure what we mean by diarrhea, tell her it means "three or more runny stools per day."***	Yes....1<br>No....0<br>No Response....-99
54	Is it you or your spouse who is military/police personnel?	1) Myself<br>2) Spouse<br>3) Son<br>4) Daughter<br>5) Other (specify): ______
55	In total how many members of your immediate family are part of the FARDC?	Number____________________________

For peer review only - http://bmjopen.bmj.com/site/about/guidelines.xhtml

Female Questionnaire

56	What is your/his rank in this service?	**1** Soldier 1st class<br>**2** Soldier 2nd class<br>**3** Corporal<br>**4** Sargent<br>**5** Sargent major<br>**6** 1st Sargent<br>**7** 1st Sargent Adjoint<br>**8** Adjudant de 2eme classe<br>**9** Adjudant<br>**10** Adjudant in chef<br>**11** Second Lieutenant<br>**12** Lieutenant<br>**13** Captain<br>**14** Major<br>**15** Lieutenant Colonel<br>**16** Colonel<br>**17** General Bragadier<br>**18** Lieutenant General<br>**19** General of Army Corps<br>**20** General or Army
57	Have you seen a billboard or banner about family planning that shows a military family?	0 No<br>1 Yes<br>88 Don't know
58	In what province were you born?<br><br>If Kinshasa, skip to question "K"	List provinces
59	For how many years have you lived in Kinshasa?<br><br>0=less than one year	____________Years
**Thank the respondent for her time**<br>*The respondent is finished, but there are still 2 more questions for you to complete outside the home.*		
**LOCATION**		
K	**Location**<br>*Take a GPS point near the entrance to the household. Record location when the accuracy is smaller than 6m.*<br>*GPS coordinates can only be collected when outside.*	Record Location
**QUESTIONNAIRE RESULT**		
L	**How many times have you visited this household to interview this female respondent?**	1st time .... 1<br>2nd time .... 2<br>3rd time 3
M	**Questionnaire result**<br>*Record the result of the Female Questionnaire*	Completed .... 1<br>Not at home .... 2<br>Postponed .... 3<br>Refused .... 4<br>Partly completed .... 5<br>Incapacitated 6

For peer review only - http://bmjopen.bmj.com/site/about/guidelines.xhtml

Household Questionnaire

NO	QUESTIONS AND FILTERS	CODING CATEGORIES	SKIP
**IDENTIFICATION**<br>**Please record the following identifying information prior to beginning the interview.**			
A	**Your name: Is this your name?**<br>[ODK will display the name associated with the phone's serial number.]<br>*Check the button next to the name if that is your name and select 'yes' here. Do not check the button if that is not your name and select 'no' here (long press to remove response next to the name if needed).*	Yes ....1<br>No 0	
	**Enter your name below.**<br>*Please record your name*	Interviewer's Name	
B	**Current date and time.**<br>[ODK will display on screen]<br>**Is this date and time correct?**	Yes ....1<br>No 0	Skip to D if Yes
C	**Record the correct date and time**	Date | Month | Day | Year<br>Time | Hour | Minutes | AM/PM	
D1	**Province**	Kinshasa .... 1<br>Kongo Central 2	
D2	**City (Kinshasa) / District (Kongo Central)**	ODK will populate the city of Kinshasa and districts in Kongo Central based on the province selected.	
D3	**Commune (Kinshasa) / Aire de santé (Kongo Central)**	ODK will populate a list of appropriate communes in Kinshasa and *aire de santé* in Kongo Central based on the city or district selected.	If Kinshasa was selected in D1, skip to D6
D4	**Quartier (Kinshasa) / Village (Kongo Central)**	ODK will populate a list of appropriate quartiers in Kinshasa and villages in Kongo Central and chiefdoms based on the commune or *aire de santé* selected.	
D5	**Enumeration area**	ODK will populate a list of appropriate enumeration areas based on the neighborhood in Kinshasa or the village in Kongo Central selected.	If Kongo Central selected in D1, skip to E
D6	**Please note the street or avenue number where the household is located**	*THE SUPERVISOR WILL GIVE YOU THE STREET OR AVENUE NAME.*	
E	**Structure number**<br>*Please record the structure number from the household listing form.*	Number [ ]	

For peer review only - http://bmjopen.bmj.com/site/about/guidelines.xhtml

Household Questionnaire

NO	QUESTIONS AND FILTERS	CODING CATEGORIES	SKIP
F	**Household number**<br>*Please record the household number from the household listing form.*	Number	
	**Check: Have you already sent a form for this structure and household?**<br>*Do not duplicate any form unless you are correcting a mistake in an earlier form.*	Yes ....1<br>No 0	Skip to G if No
	**WARNING: Contact your supervisor before sending this form again.**		
	**CHECK: Why are you resending this form?**<br>*Choose al that apply.*	There are new household members on this form ....1<br>I am correcting a mistake made on a previous form ....2<br>The previous form disappeared from my phone without being sent....3<br>I submitted the previous form and my supervisor told me that is was not received ....4<br>Other reason(s) 5	
G	**Is a member of the household and competent respondent present and available to be interviewed today?**	Yes ....1<br>No 0	Skip to 31 if No
G2	**Did this household participate in a previous PMA2020 survey?**	Yes ....1<br>No ....0<br>Do not know....-88<br>No response ....-99	

INFORMED CONSENT

Find a competent member of the household. Read the greeting on the following screen.

Hello. My name is ______________________ and I am working for the School of Public Health of Kinshasa in collaboration with the Ministry of Health. We are conducting a survey in Kinshasa and Kongo Central about various health issues in the Democratic Republic of Congo. We would very much appreciate your participation in this survey. This information will help us inform the government to better plan health services. Whatever information you provide will be kept strictly confidential and will not be shown to anyone other than members of our survey team.

Participation in this survey is voluntary, and if we should come to any question you don't want to answer, just let me know and I will go on to the next question; or you can stop the interview at any time. However, we hope that you will participate in this survey since your views are important.

I am going to ask you questions about your family and other household members. We would then like to ask a different set of questions to female members of this household who are between the ages of 15 and 49 years.

At this time, do you want to ask me anything about the survey?

NO	QUESTIONS AND FILTERS	CODING CATEGORIES	SKIP
H	Provide a paper copy of the Consent Form to the respondent and explain it. Then, ask: **May I begin the interview now?**	Yes ....1<br>No 0	Skip to 32 if No
	**Respondent's signature**<br>*Please ask the respondent to sign or check the box in agreement of their participation.*	Gather signature:<br>Check box: ☐	

For peer review only

For peer review only - http://bmjopen.bmj.com/site/about/guidelines.xhtml

Household Questionnaire

NO	QUESTIONS AND FILTERS	CODING CATEGORIES	SKIP
I	**Interviewer's name**<br>*Please record your name as a witness to the consent process. You previously entered "[NAME FROM HQ B]."*		
J	**Respondent's first name.**<br>*Please record the first name of the respondent.*		

For peer review only

For peer review only - http://bmjopen.bmj.com/site/about/guidelines.xhtml

Household Questionnaire

SECTION 1 – Household Roster

I am now going to ask you a series of questions about each usual member of the household or anyone who slept in the house last night.

No	1	2	3	4	5	6	7	8
	First name	Sex	Age (years) If less than one year old, record 0.	Marital Status	Relationship to head of household	Family ID	Is this person a usual member of the household or has he/she slept in the house last night?	Eligible female respondent
		Male .... 1<br>Female 2		Married .... 1<br>Living with a partner .. 2<br>Divorced / separated . 3<br>Widow / widower .... 4<br>Never Married .... 5<br>No response........... -99	Head .... 1<br>Wife/Husband .... 2<br>Son/Daughter .... 3<br>Son/Daughter-in-law . 4<br>Grandchild................ 5<br>Parent .... 6<br>Parent in law .... 7<br>Brother/Sister .... 8<br>Other .... 9<br>Don't know .... -88<br>No response.......... -99		Usual member of the household who slept in the house last night .... 1<br>Usual member of the household who did NOT sleep in the house last night .... 2<br>Visitor who slept in the house last night . 3<br>No response .... -99	Yes ..... 1<br>No....... 0<br><br>ODK will determine and display eligibility
1								
2								
3								
4								
5								

After recording information for one household member, the following prompt is asked to activate a looping script to record information for another member

9	Are there any other usual members of your household or persons who slept in the house last night?	Yes .... 1<br>No 0	
	**READ THIS CHECK OUT LOUD: There are [NUMBER OF HOUSEHOLD MEMBERS ENTERED] household members who are named [NAMES OF ENTERED HOUSEHOLD MEMBERS]. Is this a complete list of the household members?**<br><br>*Remember to include all children in the household.*	Yes .... 1<br>No 0	Skip to 10 if Yes

Section 2 – Household Characteristics<br>Now I would like to ask you a few questions about the characteristics of your household.				
**NO**	**QUESTIONS AND FILTERS**	**CODING CATEGORIES**		**SKIP**
10	**Please tell me about the items you're your household owns. Does your household have:**<br>*Read out all types and select all that apply. Scroll to bottom to see all choices.*<br>*If an item is reported broken but said to be out of use only temporarily, select the item. Otherwise do not select the item.*	Yes / No<br>Electricity? .... 1 / 0<br>A wall clock? .... 1 / 0<br>A radio? .... 1 / 0<br>A black/white television? .... 1 / 0<br>A color television? .... 1 / 0<br>A mobile phone? .... 1 / 0<br>A landline telephone? .... 1 / 0<br>A refrigerator? .... 1 / 0<br>A freezer? .... 1 / 0<br>A generator/invertor(s)? .... 1 / 0<br>A washing machine? .... 1 / 0<br>A computer? .... 1 / 0<br>A digital photo camera? .... 1 / 0<br>A non digital photo camera? .... 1 / 0<br>A video deck? .... 1 / 0<br>A DVD/CD? .... 1 / 0<br>A sewing machine? .... 1 / 0<br>A bed? .... 1 / 0<br>A table? .... 1 / 0<br>A cabinet/cupboard? .... 1 / 0<br>A bicycle? .... 1 / 0<br>A motorcycle or motor scooter? .... 1 / 0<br>A car or truck? .... 1 / 0<br>A boat with a motor? .... 1 / 0<br>A boat without a motor? .... 1 / 0<br>A gas or electric stove? .... 1 / 0<br>A chair(s)? .... 1 / 0<br>A lamp(s)? .... 1 / 0<br>An oven? .... 1 / 0<br>A hoe(s)? .... 1 / 0<br>Animal-drawn cart? .... 1 / 0<br>A canoe or motorized canoe? .... 1 / 0<br>A rental house? .... 1 / 0<br>None of the above .... -77<br>No response .... -99		
11a	**Does this household own any livestock, herds, other farm animals, or poultry?**<br>*These livestock can be kept anywhere, not necessarily on the homestead.*	Yes .... 1<br>No .... 0<br>No response .... -99		Skip to 12a if No or NR

For peer review only - http://bmjopen.bmj.com/site/about/guidelines.xhtml

Household Questionnaire

NO	QUESTIONS AND FILTERS	CODING CATEGORIES	SKIP
11b	**How many of the following animals does this household own?**<br>**Zero is a possible answer. Enter -88 for do not know. Enter -99 for no response.**<br>*The household can keep the livestock anywhere but must own the livestock recorded here.*	Cows or bulls [ ]<br>Horses, donkeys, or mules [ ]<br>Goats [ ]<br>Sheep [ ]<br>Hogs/pigs [ ]<br>Ducks/canes [ ]<br>Hens/cocks/other poultry [ ]<br>Other [ ]	
12a	**Does this household keep any livestock, herds, other farm animals, or poultry ON THE HOMESTEAD, regardless of who owns these livestock?**<br>*Homestead includes the structure and yard that is close to the structure.*	Yes .... 1<br>No .... 0<br>No response .... -99	Skip to 13 if No or NR
12b	**How many of the following animals does this household keep ON THE HOMESTEAD?**<br>**Zero is a possible answer. Enter -88 for do not know. Enter -99 for no response.**<br>*The household does not need to own the livestock recorded here.*	Cows or bulls [ ]<br>Horses, donkeys, or mules [ ]<br>Goats [ ]<br>Sheep [ ]<br>Hogs/pigs [ ]<br>Ducks/canes [ ]<br>Hens/cocks/other poultry [ ]<br>Other [ ]	
**Section 3 – Household Observation**<br>**Please observe the floors, roof and exterior walls.**			
13	**Main material of the floor**<br>*Observe.*	NATURAL MATERIAL<br>EARTH / SAND .... 11<br>COW DUNG .... 12<br><br>RUDIMENTARY MATERIAL<br>WOODEN BOARDS .... 21<br>PALM / BAMBOO .... 22<br><br>DEVELOPED MATERIAL<br>PARQUET OR POLISHED WOOD .. 31<br>TAPES VINYL / ASPHALT .... 32<br>TILES .... 33<br>CEMENT .... 34<br>CARPET .... 35<br>OTHER .... 96<br>NO RESPONSE .... -99	

For peer review only - http://bmjopen.bmj.com/site/about/guidelines.xhtml

NO	QUESTIONS AND FILTERS	CODING CATEGORIES	SKIP
14	**Main material of the roof**<br><br>*Observe.*	NATURAL MATERIAL<br>NO ROOF .... 11<br>THATCH/PALM ROOF .... 12<br>EARTH MOTTES .... 13<br><br>RUDIMENTARY MATERIAL<br>MATS. .... 21<br>PALM / BAMBOO .... 22<br>WOODEN BOARDS .... 23<br>CARDBOARD .... 24<br><br>DEVELOPED MATERIAL<br>SHEET METAL .... 31<br>WOOD .... 32<br>ZINC / FIBER CEMENT .... 33<br>TILE .... 34<br>CEMENT .... 35<br>SHINGLES .... 36<br>OTHER .... 96<br>NO REPONSE .... -99	
15	**Main material of the exterior walls**<br><br>*Observe.*	NATURAL MATERIAL<br>NO WALL .... 11<br>BAMBOO / CANE / PALM / TRUNK. 12<br>EARTH .... 13<br><br>RUDIMENTARY MATERIAL<br>BAMBOO WITH MUD .... 21<br>STONES WITH MUD .... 22<br>ADOBE NOT COVERED .... 23<br>PLYWOOD .... 24<br>CARDBOARD .... 25<br>RECOVERED WOOD .... 26<br><br>DEVELOPED MATERIAL<br>CEMENT .... 31<br>STONES WITH LIME / CEMENT ... 321<br>BRICKS .... 332<br>CEMENT BLOCKS .... 343<br>COVERED ADOBE .... 354<br>WOOD BOARD / SHINGLES .... 365<br>OTHER .... 96<br>NO RESPONSE .... -99	
**Section 4 – Water, Sanitation and Hygiene**<br>**Now I would like to ask you a few questions about water, sanitation and hygiene.**			
16	**Do you have a place to wash your hands, or do you have a movable container that is not kept in a fixed location, such as a bowl or kettle, that is commonly used for hand washing?**<br><br>*If the container is always in the same location, then count it as a fixed place*	Yes, fixed place .... 1<br>Yes, movable container .... 2<br>No .... 0<br>Don't know .... -88<br>Refuse to answer .... -99	Skip to 19 if No<br><br>Go to 17a if 16 is 1<br><br>Skip to 17b if 16 is 2
17a	**Can you show it to me?**	Yes .... 1<br>No 0	Skip to 19 if No

For peer review only - http://bmjopen.bmj.com/site/about/guidelines.xhtml

Household Questionnaire

NO	QUESTIONS AND FILTERS	CODING CATEGORIES	Yes	No	SKIP
18a	**AT THE PLACE WHERE THE HOUSEHOLD WASHES THEIR HANDS, OBSERVE IF:**	Soap is present ....<br>Water source is present: stored water ....<br>Water source is present: running water ....<br>Hand washing area is near a sanitation facility ....<br>None of the above	1<br>1<br>1<br>1<br>-77	0<br>0<br>0<br>0	
17b	**Can you show me any soap, water, and movable container available in the household used for hand washing?**	Yes .... 1<br>No 0			
18b	**ANYWHERE WITHIN THE HOUSEHOLD, OBSERVE IF:**	Soap is present ....<br>Water source is present: stored water ....<br>Water source is present: running water ....<br>Hand washing container Is observed ....<br>None of the above	1<br>1<br>1<br>-77	0<br>0<br>0	
19	**Which of the following water sources does your household use on a regular basis for any part of the year for any purpose?**<br>*Read out all types and check all that are used. Scroll to the bottom to see all choices.*	Piped Water<br>Piped into dwelling/indoor ....<br>Pipe to yard/plot ....<br>Public tap/standpipe ....<br>Tube well or borehole ....<br>Dug Well<br>Protected Well ....<br>Unprotected Well ....<br>Water from Spring<br>Protected Spring ....<br>Unprotected Spring ....<br>Rainwater ....<br>Tanker Truck ....<br>Cart with Small Tank ....<br>Surface water (River / Dam / Lake / Pond / Stream /Canal / Irrigation Channel) ....<br>Bottled Water ....<br>Sachet Water ....<br>No Response	1<br>1<br>1<br>1<br>1<br>1<br>1<br>1<br>1<br>1<br>1<br>1<br>1<br>1<br>-99	0<br>0<br>0<br>0<br>0<br>0<br>0<br>0<br>0<br>0<br>0<br>0<br>0<br>0	If only one source is selected, skip to HQ22
20	**What is the main source of drinking water for members of your household?**<br>**Selections from HQ19: [ODK will list water sources selected for HQ19]**<br>*Read out HQ19 selections only.*	Piped Water<br>Piped into dwelling/indoor ....1<br>Pipe to yard/plot ....2<br>Public tap/standpipe ....3<br>Tube well or borehole ....4<br>Dug Well<br>Protected Well ....5<br>Unprotected Well ....6<br>Water from Spring<br>Protected Spring ....7<br>Unprotected Spring ....8<br>Rainwater .... 9<br>Tanker Truck ....10<br>Cart with Small Tank ....11<br>Surface water<br>(River / Dam / Lake / Pond / Stream / Canal / Irrigation Channel) .... 12<br>Bottled Water ....13<br>Sachet Water ....14<br>No Response ....-99			

For peer review only - http://bmjopen.bmj.com/site/about/guidelines.xhtml

Household Questionnaire

NO	QUESTIONS AND FILTERS	CODING CATEGORIES	SKIP
21	**What is the main source of water used by your household for other purposes such as cooking and hand washing?**<br><br>**Selections from HQ19: [ODK will list water sources selected for HQ19]**<br><br>*Read out HQ19 selections only.*	Piped Water<br>Piped into dwelling/indoor ....1<br>Pipe to yard/plot ....2<br>Public tap/standpipe ....3<br>Tube well or borehole ....4<br>Dug Well<br>Protected Well ....5<br>Unprotected Well ....6<br>Water from Spring<br>Protected Spring ....7<br>Unprotected Spring ....8<br>Rainwater.. 9<br>Tanker Truck ....10<br>Cart with Small Tank ....11<br>Surface water<br>(River / Dam / Lake / Pond / Stream<br>/ Canal / Irrigation Channel) .... 12<br>Bottled Water ....13<br>Sachet Water ....14<br>No Response ....-99	
	**Questions HQ 22 to HQ 25 will repeat x times, once for each water source selected in HQ 19. These sources include:**<br><br>**[ODK will display HQ19 selections.]**		
22	**You mentioned that you used [WATER SOURCE]. At any time of the year, does your household use water from this source for:**	Yes / No<br>Drinking .... 1 / 0<br>Cooking .... 1 / 0<br>Livestock .... 1 / 0<br>Gardening / agriculture .... 1 / 0<br>Business venture.......................... 1 / 0<br>Washing .... 1 / 0<br>No response........................ -99	
23	**Is [WATER SOURCE] typically available:**<br><br>*Read all choices out loud.*	All of the year .... 1<br>Some of the year...................................... 2<br>Small part of the year .... 3<br>No response...-99	
24	**At a time of year when you expect to have water from [WATER SOURCE], is it usually available?**	Yes, always .... 1<br>No, intermittent and predictable .... 2<br>No, intermittent and unpredictable .... 3<br>No response...-99	
25	**How long does it take to go to [WATER SOURCE], get water, and come back?**<br><br>*Zero is a possible answer*<br>*Enter -88 for do not know*<br>*Enter -99 for no response*<br><br>*Convert time into minutes.*<br>*Answer includes waiting time in line.*	Minutes: [ ]	
26	**Does your household have a garden?**<br><br>*A garden is a place to grow vegetables.*	Yes .... 1<br>No... 0<br>No response...-99	

For peer review only - http://bmjopen.bmj.com/site/about/guidelines.xhtml

Household Questionnaire

NO	QUESTIONS AND FILTERS	CODING CATEGORIES			SKIP
27	**Do members of your household use any of the following toilet facilities?**<br>*Read out all types and check all that are used. Scroll to the bottom to see all choices.*		Yes	No	
		Flush/pour flush toilets connected to:			
		Piped sewer system	1	0	
		Septic tank	1	0	
		Pit Latrine	1	0	
		Elsewhere	1	0	
		Unknown / Not sure / Don't know	1	0	
		Ventilated improved pit latrine	1	0	
		Pit latrine with slab	1	0	
		Pit latrine without slab	1	0	
		Bucket toilet	1	0	
		Composting toilet	1	0	
		Hanging toilet /Hanging latrine	1	0	
		No facility / bush / field	1	0	
		Other:	1	0	
		No Response	-99		
28	**What is the main toilet facility used by members of your household?**<br>**HQ27: [ODK will display HQ27 selections]**<br>*The main facility must be selected in HQ 27.*	Flush/pour flush toilets connected to:<br>Piped sewer system .... 1<br>Septic tank .... 2<br>Pit Latrine .... 13<br>Elsewhere .... 3<br>Unknown / Not sure / Don't know ..... 4<br>Ventilated improved pit latrine .... 5<br>Pit latrine with slab .... 6<br>Pit latrine without slab/open pit .... 7<br>Bucket toilet .... 8<br>Composting toilet .... 9<br>Hanging toilet /Hanging latrine .... 10<br>No facility / bush / field .... 11<br>Other: .... 12<br>No Response .... -99			
	**Question HQ 29 will repeat x times, once for each sanitation facility selected in HQ27. These facilities include:**<br>**HQ27: [ODK will display HQ27 selections]**				
29	**How often does your household typically use: [TOILET FACILITY TYPE]?**<br>*Regular practices at the household only.*	Always .... 1<br>Most of the time .... 2<br>Occasionally .... 3<br>Rarely .... 4<br>No response .... -99			
29b	**Do you share this toilet facility with other households or the public?**	Not shared .... 1<br>Shared with less than ten households .... 2<br>Shared with ten or more households .... 3<br>Shared with the public. .... 4<br>No response .... -99			Skip to HQ30 if not 2

For peer review only - http://bmjopen.bmj.com/site/about/guidelines.xhtml

Household Questionnaire

NO	QUESTIONS AND FILTERS	CODING CATEGORIES	SKIP
29c	**Enter the number of households that share this facility (including your own).**<br>**[TOILET FACILITY TYPE]**<br>*Must be between 2 and 9.*<br>*If 10 or greater, swipe back to HQ29b and choose "shared with ten or more households."*<br>*Enter -99 for no response.*	Number of Households: ☐	
30	**How many people within your household regularly use the bush / field at home or at work?**<br>**There are x people in this household. Enter -88 for do not know, -99 for no response.**	Number of People: ☐	
31	**Ask permission to take a photo of the entrance of the house.**<br>**Did you get consent to take the photo?**	Yes .... 1<br>No 0	Skip L if No
**Thank the respondent for her/his time.**<br>*The respondent is finished, but there is still more for you to complete outside the home.*			
**LOCATION AND QUESTIONNAIRE RESULT**			
K	**Location**<br>**Take a GPS point near the entrance to the household. Record location when the accuracy is smaller than 6m.**	RECORD LOCATION	
	**CHECK 32:** Permission to take photo?		Skip to M if No
L	**Ensure that no people are in the photo**	TAKE PICTURE<br>CHOOSE IMAGE	
M	**How many times have you visited this household?**	1st time .... 1<br>2nd time .... 2<br>3rd time 3	
N	**Questionnaire result**<br>*Record the result of the Household Questionnaire*	Completed .... 1<br>No household member at home or no competent respondent at home at time of visit .... 2<br>Postponed .... 3<br>Refused .... 4<br>Partly completed .... 5<br>Dwelling vacant or address not a dwelling .... 6<br>Dwelling destroyed .... 7<br>Dwelling not found .... 8<br>Entire household absent for extended period of time 9	

For peer review only - http://bmjopen.bmj.com/site/about/guidelines.xhtml

STROBE Statement—Checklist of items that should be included in reports of ***cross-sectional studies***

	Item No	Recommendation
**Title and abstract**	1	(*a*) Indicate the study's design with a commonly used term in the title or the abstract
		(*b*) Provide in the abstract an informative and balanced summary of what was done and what was found
**Introduction**		
Background/rationale	2	Explain the scientific background and rationale for the investigation being reported
Objectives	3	State specific objectives, including any prespecified hypotheses
**Methods**		
Study design	4	Present key elements of study design early in the paper
Setting	5	Describe the setting, locations, and relevant dates, including periods of recruitment, exposure, follow-up, and data collection
Participants	6	(*a*) Give the eligibility criteria, and the sources and methods of selection of participants
Variables	7	Clearly define all outcomes, exposures, predictors, potential confounders, and effect modifiers. Give diagnostic criteria, if applicable
Data sources/ measurement	8*	For each variable of interest, give sources of data and details of methods of assessment (measurement). Describe comparability of assessment methods if there is more than one group
Bias	9	Describe any efforts to address potential sources of bias
Study size	10	Explain how the study size was arrived at
Quantitative variables	11	Explain how quantitative variables were handled in the analyses. If applicable, describe which groupings were chosen and why
Statistical methods	12	(*a*) Describe all statistical methods, including those used to control for confounding
		(*b*) Describe any methods used to examine subgroups and interactions
		(*c*) Explain how missing data were addressed
		(*d*) If applicable, describe analytical methods taking account of sampling strategy
		(*e*) Describe any sensitivity analyses
**Results**		
Participants	13*	(a) Report numbers of individuals at each stage of study—eg numbers potentially eligible, examined for eligibility, confirmed eligible, included in the study, completing follow-up, and analysed
		(b) Give reasons for non-participation at each stage
		(c) Consider use of a flow diagram
Descriptive data	14*	(a) Give characteristics of study participants (eg demographic, clinical, social) and information on exposures and potential confounders
		(b) Indicate number of participants with missing data for each variable of interest
Outcome data	15*	Report numbers of outcome events or summary measures
Main results	16	(*a*) Give unadjusted estimates and, if applicable, confounder-adjusted estimates and their precision (eg, 95% confidence interval). Make clear which confounders were adjusted for and why they were included
		(*b*) Report category boundaries when continuous variables were categorized
		(*c*) If relevant, consider translating estimates of relative risk into absolute risk for a meaningful time period
Other analyses	17	Report other analyses done—eg analyses of subgroups and interactions, and sensitivity analyses

For peer review only

For peer review only - http://bmjopen.bmj.com/site/about/guidelines.xhtml

**Discussion**		
Key results	18	Summarise key results with reference to study objectives
Limitations	19	Discuss limitations of the study, taking into account sources of potential bias or imprecision. Discuss both direction and magnitude of any potential bias
Interpretation	20	Give a cautious overall interpretation of results considering objectives, limitations, multiplicity of analyses, results from similar studies, and other relevant evidence
Generalisability	21	Discuss the generalisability (external validity) of the study results
**Other information**		
Funding	22	Give the source of funding and the role of the funders for the present study and, if applicable, for the original study on which the present article is based

*Give information separately for exposed and unexposed groups.

Note: An Explanation and Elaboration article discusses each checklist item and gives methodological background and published examples of transparent reporting. The STROBE checklist is best used in conjunction with this article (freely available on the Web sites of PLoS Medicine at http://www.plosmedicine.org/, Annals of Internal Medicine at http://www.annals.org/, and Epidemiology at http://www.epidem.com/). Information on the STROBE Initiative is available at www.strobe-statement.org.

For peer review only

BMJ Open

Differences in Family Planning Outcomes between Military and General Populations in Kinshasa, Democratic Republic of Congo: A Cross-Sectional Analysis

Journal:	*BMJ Open*
Manuscript ID	bmjopen-2018-022295.R2
Article Type:	Research
Date Submitted by the Author:	20-Sep-2018
Complete List of Authors:	Akilimali, Pierre; Universite de Kinshasa, Kinshasa School of Public Health<br>Anglewicz, Philip; Tulane University School of Public Health and Tropical Medicine, Department of Global Community Health and Behavioral Science;<br>Engale, Henri; Congolese Armed Forces, Medical Division<br>Kurhenga, Gilbert; Congolese Armed Forces, Medical Division<br>Hernandez, Julie; Tulane University, School of Public Health and Tropical Medicine<br>Kayembe, Patrick; University of Kinshasa, Kinshasa School of Public Health<br>Bertrand, Jane; Tulane University
**Primary Subject Heading**:	Global health
Secondary Subject Heading:	Reproductive medicine, Public health
Keywords:	Military, Fertility, Family planning, Contraceptive use, Kinshasa, Democratic Republic of Congo

SCHOLARONE™
Manuscripts

Differences in Family Planning Outcomes between Military and General Populations in Kinshasa, Democratic Republic of Congo: A Cross-Sectional Analysis

Authors

Pierre Akilimali
Kinshasa School of Public Health, University of Kinshasa, BP 11850 Kinshasa 1, Democratic Republic of Congo. Email: pierretulanefp@gmail.com.

Philip Anglewicz (corresponding author)
School of Public Health and Tropical Medicine, Tulane University, 1440 Canal St Suite 2210, New Orleans, LA, USA. Email: panglewi@tulane.edu Phone: (504) 988-5428; Fax: (504) 988-5480.

Henri Nzuka Engale
Medical Division, Congolese Armed Forces, Email: nzukaengale@yahoo.fr

Gilbert Kabanda Kurhenga
Chief, Medical division, Congolese Armed Forces, Email: gilkaku@yahoo.fr

Julie Hernandez
School of Public Health and Tropical Medicine, Tulane University, 1440 Canal St Suite 1900, New Orleans, LA, USA. Email: jhernan7@tulane.edu

Patrick Kayembe
Kinshasa School of Public Health, University of Kinshasa, BP 11850 Kinshasa 1, Democratic Republic of Congo. Email: patkayembe@yahoo.fr

Jane Bertrand
School of Public Health and Tropical Medicine, Tulane University, 1440 Canal St Suite 1900, New Orleans, LA, USA. Email: bertrand@tulane.edu

For peer review only

For peer review only - http://bmjopen.bmj.com/site/about/guidelines.xhtml

Abstract:

Objectives: To examine family planning outcomes among women living in military camps in Kinshasa, Democratic Republic of Congo, and compare these outcomes with a representative sample of non-military women in Kinshasa.

Participants: Women of reproductive ages, 15 to 49. We compare two populations: women living in military camps and the general (non-military) population in Kinshasa.

Study Design: For sampling, we used a two-stage cluster sampling design, where we first randomly selected enumeration areas (EA), and then randomly selected women within each EA (separately for each of the two populations). We administered a survey on contraceptive use and family planning to all participating women. We use bivariate and multivariate analysis to compare these populations for a range of family planning outcomes.

Results: We find many statistically significant differences between women in military camps and general female population of Kinshasa. Although they do not have more children, women in military camps are less likely to be using contraception (all methods OR 0.24, 95% CI 0.11-0.53; modern methods OR 0.25, 95% CI 0.08-0.79; traditional methods OR 0.41, 95% CI 0.24-0.71) and less knowledgeable about many family planning methods (less likely to have heard of implants (OR 0.23, 95% CI 0.11-0.48), injectables (OR 0.19, 95% CI 0.08-0.44), condoms (OR 0.23, 95% CI 0.12-0.47), withdrawal (OR 0.05, 95% CI 0.02-0.17), and rhythm (OR 0.12, 95% CI 0.03-0.44) methods), while at the same time they are more likely to want to limit their births (OR 5.17, 95% CI 2.52, 10.62), and less likely to have obtained their preferred family planning method (OR 0.14, 95% CI 0.03, 0.64).

Conclusions: Women in military camps in Kinshasa appear to be an important and underserved population with regards to family planning. Our results suggest that women in military camps have limited access to modern family planning methods.

Article Summary:

Strengths and limitations of this study:

- Examine family planning outcomes for an important but understudied population, women in military camps in a high-fertility setting;
- Use representative data for both military camp and general population in Kinshasa;
- Lacking some measures importantly related to family planning in this environment.

Key words:

Military, fertility, family planning, contraceptive use, Kinshasa, Democratic Republic of Congo.

Word count: 2922

For peer review only - http://bmjopen.bmj.com/site/about/guidelines.xhtml

Introduction

Research on family planning practices among the military population in developing countries is limited. During the HIV/AIDS epidemic, the military was seen as a population at particular risk of infection and was therefore the topic of HIV-related research (e.g., [1–8]). But research on family planning among the military in developing settings has been conducted in few geographic areas, such as India,[9] and Nigeria.[8,10–13] Furthermore, the scant existing research has important limitations, such as the lack of a comparison group from the general population, so it's often not known if the military population is at particular need of family planning programs.[8-13]

In order to increase knowledge and use of contraceptive methods, programs often target specific sub-populations that have lower contraceptive use and access, such as poor, urban, or adolescent female populations (e.g., [14–16]). These populations then become the subjects for targeted family planning programs, in order to improve overall population-level fertility and family planning outcomes. However, some groups that may differ in family planning outcomes are seldom examined; the military is one such population.

There is reason to believe that the military population is highly relevant for family planning research and programs. In some settings, the military population composes a non-negligible percentage of the overall population and may therefore have a noticeable impact on aggregate family planning measures. The military also may have different family planning practices than the general population: research in the United States has shown that the military population has relatively higher fertility and earlier family formation.[17]

The relationship between military participation and fertility is ambiguous. Some research has shown that the military is an environment that promotes family formation, and military members marry earlier and higher fertility.[17] Benefits offered for military families can facilitate childbearing.[17] As a result, it's perhaps not surprising that most studies show high rates of pregnancy and low contraceptive use among military in the United States.[18–21]

For peer review only - http://bmjopen.bmj.com/site/about/guidelines.xhtml

There are also reasons why one might expect lower fertility and greater contraceptive use among female members of the military. Pregnancy inhibits the ability to train for and serve in active duty,[22] and has led to evacuation from military activity for female troops.[23] Military activity may separate spouses for extended periods, thereby limiting opportunity for childbearing.[24] Military service can also cause stress within marriage, which may impact fertility.[24] Some studies have found higher use of oral contraceptive in the military.[22]

In this research we examine family planning-related outcomes among women in military camps in Kinshasa, Democratic Republic of Congo. We focus on a range of family planning outcomes, such as fertility, contraceptive use (overall, modern and traditional), whether the last birth was unintended, desire for an additional child, family planning knowledge, and exposure to family planning messages. We compare family planning outcomes for the female military camp population in Kinshasa with a comparable survey of that is representative of the general, non-military female population in Kinshasa.

Methods

Setting

The DRC is Africa's fourth most-populous and one of the region's fastest growing countries.[25] DRC has one of the highest fertility rates in the world: the most recent Demographic and Health Survey (DHS) from 2013-14 estimated a country-level TFR of 6.6, a slight increase since the 6.3 TFR estimated from the 2007 DHS.[26-27] At the same time, contraceptive use is low in DRC: the modern contraceptive prevalence rate (mCPR) among women age 15-49 who are married or in union is 7.8% for the country as a whole, 19.0% in Kinshasa and 17.2% in Kongo Central.[27]

Military camps are located throughout the city of Kinshasa. According to government documents obtained by the authors, there are 17 military camps in Kinshasa, in which both enlisted military and their families reside. According to 2016 estimates, the total population of these camps is 305,405, which represents approximately 3% of the population of Kinshasa. The vast majority of enlisted military are male; only 4% of military members are women. Military camps are closed environments and difficult to access by the civilian population, as such they are often not targeted by mass health activities. Although the majority of enlisted military live in

For peer review only - http://bmjopen.bmj.com/site/about/guidelines.xhtml

camps, because housing and other services are free, not all members of the military reside there; officers in the military often live outside. All military camps have a health center, which can be used by military members and their families.

Data

We use two sources of data for this analysis. Our first source is the Performance Monitoring and Accountability 2020 Project (PMA2020), which was established in part to measure uptake of contraceptive use in many of the world's most populous countries (http://www.pma2020.org/). To achieve this aim, PMA2020 collects representative data in eleven countries on an annual basis for a range of fertility and family planning-related measures.

PMA2020 has collected data from two provinces in DRC, six rounds of data in Kinshasa (2013-2017), and three rounds in Kongo Central (2015-2017), a province to the west of Kinshasa. The sampling framework uses a two-stage cluster sampling approach, in which the study first randomly selects census enumeration areas within each province, then conducts a listing of all households in these EAs, and randomly selects 33 households within each EA. PMA2020 first administers a household survey to the head of household, and then all resident women of reproductive age (15-49 years) within the household are selected for interview. The PMA2020 female survey includes basic demographic information and extensive information on fertility history and preferences, and contraceptive use. In 2016, PMA2020 interviewed 2,607 women in its fifth round of data collection in Kinshasa. Interviews were conducted only with female interviewers.

Data collection for the military sample also took place in 2016. In sampling this population, we used a similar two-stage cluster design approach. Out of the 17 military camps in Kinshasa, ten were randomly drawn, proportionate to population size. These ten military camps were then divided into enumeration areas (or EAs), and one EA was randomly drawn in each of the ten camps. As with PMA2020, interviewers first conducted a listing of households in each EA, after which 33 households in each EA were selected for interview (with all residing women aged 15-49 sought for interview). A total of 514 women were interviewed in the military sample. Of

For peer review only - http://bmjopen.bmj.com/site/about/guidelines.xhtml

these women, the majority were spouses of men in the military (78.6%), while 16.6% were military members themselves. The remaining women were mothers of a military member.

This study has received approval to collect data from Institutional Review Boards at Johns Hopkins University, Tulane University, and the University of Kinshasa. All participating women provided written and informed consent to take part in this study, and consent procedures were approved by Institutional Review Boards at all three institutions. In this setting, individuals aged 15-17 are considered adults, so parental consent was not necessary to interview women of these ages.

Measures

We focus on several categories of family planning outcomes, starting with four fertility-related outcomes: the number of lifetime births, whether the woman experienced the death of one of her children, whether the women does not want another child, and whether the last birth was unintended. Next, we turn to family planning use, measured as overall contraceptive use, then separated into modern and traditional methods (rhythm, withdrawal, and other traditional method (folkloric methods like amulets, herbs, etc…)). Among those using contraception, we examine whether women obtained their desired method, phrased as "During that visit, did you obtain the method you wanted to delay or avoid getting pregnant?" Finally, we measure family planning awareness and exposure; whether the respondent had heard of injectables, implants, condoms, withdrawal, and rhythm methods; and whether the woman had visited a health facility in the past 12 months, heard about family planning on the radio, read about family planning in the newspaper, or saw family planning billboard.

We also examine demographic characteristics for women in military camps and general female population in Kinshasa, including age, number of lifetime births, marital status, level of education, and household wealth. Household wealth is measured using a constructed wealth index based on ownership of 25 household durable assets, house and roof material, livestock ownership and water source. A wealth index was created using principal component analysis,[28] which is then converted into quintiles. Survey instruments are included as supplementary files.

For peer review only - http://bmjopen.bmj.com/site/about/guidelines.xhtml

Analysis

First, we tabulate background and family planning characteristics for women in military camps and the female non-military population of Kinshasa. We account for the sampling design from data collection, using weights and accounting for clustering within enumeration areas.

Next, we examine whether family planning differences persist, after controlling for background characteristics that may differ between women in military camps and the non-military population. We use multivariate regressions where the dependent variables are measures of interest for family planning, including number of lifetime births, use of contraception (overall contraceptives, modern contraceptives, traditional methods), not wanting another child, experiencing child mortality, and last birth unintended. Independent variables include age, a quadratic term for age, level of education, marital status, and household wealth quintile. To account for the study design, we cluster standard errors by enumeration area. Missing values are considered missing at random.

Patient and Public Involvement

Patients or public were not involved in the study design, development of the research question, recruitment into or conduct of the study, or outcome measures. Results will not be distributed to the participants themselves.

Results

Background characteristics (weighted) for women from both rounds of PMA2020 are shown in Table 1, including statistical tests for differences between women in military camps and the non-military population. Generally, women in military camps and the non-military population are similar in their demographic profile, there are no statistically significant differences in background characteristics.

Although the populations are similar in demographic characteristics, they are very different in family planning-related outcomes (Table 1). Women in military camps are significantly less likely to use contraception, and are significantly less likely to obtain their desired contraceptive

For peer review only - http://bmjopen.bmj.com/site/about/guidelines.xhtml

method. As shown in Table 1, women in military camps are more likely to not want another child, and to claim that their last birth was unintended. Women in military camps are less informed of family planning methods; they are significantly less likely to have heard of implants, injectables, condoms, withdrawal, and rhythm. However, results for exposure to family planning programs are mixed: they are less likely to have visited a health facility in the past 12 months but more likely to have heard about FP on the radio, read about it in a newspaper, and saw a family planning billboard.

After controlling for demographic characteristics, the multivariate results are similar to the bivariate differences between women in military camps and the non-military population. As shown in Table 2, there is no statistically significant difference in number of lifetime births between women in military camps and the non-military population. However, women in military camps are significantly less likely to have experienced child mortality than the non-military (OR 0.53, 95% CI 0.33-0.87). Women in military camps are significantly more likely to report that they do not want another child (OR 5.17, 95% CI 2.52, 10.62), and to report that their last birth was unintended (OR 5.19, 95% CI 2.31, 11.62).

Results in Table 3 confirm the differences in contraceptive use for these populations, in which women in military camps are significantly less likely to be using contraception of all types, modern methods, and traditional methods (all methods OR 0.24, 95% CI 0.11-0.53; modern methods OR 0.25, 95% CI 0.08-0.79; traditional methods OR 0.41, 95% CI 0.24-0.71). Women in military camps were also significantly less likely to have obtained their desired family planning method (OR 0.14, 95% CI 0.03, 0.64).

We also find consistent differences in knowledge of various contraceptive methods (Table 4), both modern and traditional. Women in military camps are significantly less likely to have heard of implants (OR 0.23, 95% CI 0.11-0.48), injectables (OR 0.19, 95% CI 0.08-0.44), condoms (OR 0.23, 95% CI 0.12-0.47), withdrawal (OR 0.05, 95% CI 0.02-0.17), and rhythm (OR 0.12, 95% CI 0.03-0.44) methods.

For peer review only - http://bmjopen.bmj.com/site/about/guidelines.xhtml

Results for exposure to family planning are shown in Table 5. Women in military camps are less likely to have visited a health facility in the past 12 months (OR 0.28, 95% CI 0.14-0.55). However, they are significantly more likely to have heard about family planning on the radio (OR 2.81, 95% CI 1.34-5.92), read about family planning in a newspaper or magazine (OR 7.72, 95% CI 3.13-19.04), and saw a family planning billboard (OR 9.71, 95% CI 3.63-25.20).

Discussion

Although the female population in military camps is similar to the general population of women in Kinshasa in background characteristics, we find many strong and highly statistically significant differences in family planning-related outcomes. Overall, these results suggest that the population residing in military camps is an important, underserved population in Kinshasa: the female population in military camps are more likely to want to limit births but are less likely to be using contraception, and less likely to have obtained their preferred family planning method. Women in military camps are more likely to be exposed to family planning messages (radio, billboard, newspaper/magazine), but are less knowledgeable about many family planning methods.

These results suggest that women in military camps have limited access to modern family planning methods. Lack of access would explain why they are less likely to use despite being more likely to want to limit births. Access limitations are also evident in the fact that women in military camps are less likely to have obtained their preferred family planning method, and are less likely to have visited a health facility in the past 12 months. Prior to this study, leadership in the DRC military (co-authors on this paper) recognized a lack of access to and limited use of modern contraception among the military population. They actively sought out a research partner to document this trend and a service delivery organization that could work with them in improving contraceptive services within the military camps of Kinshasa and Kongo Central. Our study confirms the expectations of the DRC military that contraceptive use is lower among women in military camps, due at least in part to limited access to modern family planning.

The lack of access does not, however, explain the lower use or traditional methods and the greater exposure to family planning messaging among the military camp population. This may

For peer review only - http://bmjopen.bmj.com/site/about/guidelines.xhtml

be explained by the timing of family planning programs. According to our authors, new family planning programs were advertised in military camps recently prior to our data collection, but this was not yet matched by supply of family planning methods in military camp clinics.

This study also serves as a baseline for a programmatic initiative that started shortly after the survey in Kinshasa, which consisted of training clinical personnel at the military health center in all contraceptive methods, including Implanon NXT (highly popular in Kinshasa); training and supplying community-health workers with a non-medical profile to distribute pills, condoms and Cyclebeads at the community level; and placing billboards promoting FP near military camps that showed the father in uniform. A follow-up survey is scheduled for 2018 to assess change in FP access and use.

The strengths of this study are the rare opportunity to learn more about a neglected, but important, population for family planning; and the representative data for residents of military camps and general population of Kinshasa. However, there remain some limitations. Several measures of interest are not included in our survey instrument. Many military members and their families are likely in-migrants to Kinshasa from elsewhere in DRC; research in this setting has shown that migrants differ from Kinshasa-born residents in many characteristics related to family planning.[29] This study, unfortunately, did not measure migration status, birthplace, or duration resided in Kinshasa. Similarly, measures of access to a facility that provides family planning, such as distance to the nearest family planning clinic or pharmacy that has modern methods in stock, were not measured for the military camp population, but may differ from the general population- as suggested by our results.

Contributors: PA, PA and JB initially conceived the manuscript. PA and PA conducted the statistical analysis and wrote the first draft of the paper. HNE, GKK, JH, PK, and JB reviewed the paper before submission and provided comments and edits.

Funding statement: PMA2020 was supported by the Bill & Melinda Gates Foundation, Seattle, WA; under grant #OPP1079004. Data collection for military camps in Kinshasa was also supported by the Bill & Melinda Gates Foundation, Seattle, WA; under grant # OPP1128892.

For peer review only - http://bmjopen.bmj.com/site/about/guidelines.xhtml

Competing interests statement: the authors declare no competing interests.

Data sharing statement: All data collected under PMA2020 is made publically-available. More details on data access for PMA2020 is available here: http://www.pma2020.org/data-use.

For peer review only

For peer review only - http://bmjopen.bmj.com/site/about/guidelines.xhtml

Figures and Tables

Table 1: Weighted background characteristics for military and non-military women, Kinshasa 2016

	Non-military	Military
**Age groups**		
15-20	21.60%	24.48%
21-25	20.94%	20.02%
26-30	16.69%	18.42%
31-35	14.42%	15.85%
36-40	11.54%	10.51%
41-45	8.53%	5.87%
46-49	6.28%	4.85%
**Level of education**		
No education	2.16%	4.31%
Primary	18.55%	13.53%
Middle secondary school	63.56%	66.76%
Advanced secondary or higher	15.72%	15.40%
**Marital status**		
Married/partnered	47.03%	48.16%
Separated/divorced/widowed	5.32%	4.75%
Never married	47.65%	47.09%
**Wealth index**		
Quintile 1 (lowest)	15.09%	26.43%
Quintile 2	19.33%	27.02%
Quintile 3	21.28%	20.65%
Quintile 4	21.33%	15.05%
Quintile 5 (highest)	22.97%	10.85%
**Fertility and children**		
Number lifetime births	1.82	1.71
Experienced child mortality	19.65%	12.44%*
Do not want another child	18.03%	46.72%**
Last birth unintended	7.36%	26.29%**
**Family planning use**		
Contraceptive use	42.28%	17.43%**
Traditional contraceptive use	21.39%	6.56%*
Modern contraceptive use	20.89%	10.87%**
Obtained desired the FP method	92.41%	68.25%**
**Family planning knowledge**		
Heard of implants	83.27%	55.82%**
Heard of injectables	87.83%	60.91%**
Heard of condoms	94.73%	81.91%**
Heard of withdrawal	80.83%	46.94%**
Heard of rhythm	91.50%	44.57%**
**Family planning exposure**		
Visited health facility in past 12 months	55.18%	28.84%**
Heard about FP on the radio	34.65%	58.58%*

For peer review only - http://bmjopen.bmj.com/site/about/guidelines.xhtml

Read about FP in a magazine/newspaper	13.12%	50.76%**
Saw FP billboard advertisement	46.12%	89.34%**
N=	2607	514

Notes: Difference between military and non-military significant at *$p \leq 0.05$; **$p \leq 0.01$.

For peer review only

For peer review only - http://bmjopen.bmj.com/site/about/guidelines.xhtml

Table 2: Weighted regression results for differences in fertility and children between military and non-military, Kinshasa 2016

	Number of births		Child mortality		Do not want another		Last birth unintended	
	Coef	95% CI	Odds	95% CI	Odds	95% CI	Odds	95% CI
**Military**	**-0.05**	**-0.31, 0.22**	**0.53***	**0.33, 0.87**	**5.17****	**2.52, 10.62**	**5.19****	**2.31, 11.62**
Number of births	----	----	1.16**	1.05, 1.29	1.55**	1.31, 1.84	1.43**	1.23, 1.66
Age	0.33**	0.28, 0.37	1.03**	1.01, 1.06	1.02*	1.00, 1.05	0.99	0.96, 1.03
Age squared	-0.01**	-0.00, -0.00	----	----	----	----	----	----
Level of education								
No education (ref.)	----	----	----	----	----	----	----	----
Primary	0.13	-0.13, 0.38	1.05	0.50, 2.20	0.57	0.14, 1.07	1.01	0.14, 1.07
Middle secondary school	0.03	-0.28, 0.33	0.81	0.34, 1.93	0.48	0.20, 1.16	0.51	0.19, 1.32
Advanced secondary or higher	-0.37*	-0.73, -0.02	0.43	0.16, 1.18	0.39	0.14, 1.07	0.67	0.23, 1.98
Marital status								
Married/partnered (ref.)	----	----	----	----	----	----	----	----
Separated/divorced/widowed	-0.16**	-0.28, -0.05	0.72	0.40, 1.29	0.88	0.51, 1.53	2.82**	1.47, 5.42
Never married	-1.06**	-1.32, -0.80	1.03	0.67, 1.59	1.06	0.64, 1.75	2.81*	1.19, 6.66
Household wealth								
Quintile 1 (lowest, ref.)	----	----	----	----	----	----	----	----
Quintile 2	-0.13	-0.27, 0.01	0.85	0.48, 1.50	0.94	0.52, 1.63	0.54	0.20, 1.48
Quintile 3	-0.16*	-0.31, -0.01	0.88	0.57, 1.37	0.92	0.19, 1.26	0.69	0.26, 1.83
Quintile 4	-0.12	-0.29, 0.04	0.67	0.41, 1.10	0.48	0.31, 1.22	1.01	0.47, 2.16
Quintile 5 (highest)	-0.23**	-0.40, -0.06	0.77	0.44, 1.35	0.61	0.50, 1.64	0.82	0.30, 2.27
N=	3085		1795		2830		1813	

Notes: *p ≤ 0.05; **p ≤ 0.01; child mortality and last birth unintended are limited to women who have ever given birth.

For peer review only

Table 3: Weighted logistic regression results for differences in family planning use for military, Kinshasa 2016

	Using Contraception		Modern Contraception		Traditional Contraception		Obtained desired method	
	**Odds**	**95% CI**	**Odds**	**95% CI**	**Odds**	**95% CI**	**Odds**	**95% CI**
**Military**	**0.24****	**0.11, 0.53**	**0.25***	**0.08, 0.79**	**0.41****	**0.24, 0.71**	**0.14****	**0.03, 0.64**
Number of births	1.29**	1.17, 1.43	1.16**	1.05, 1.28	1.22**	1.13, 1.32	0.98	0.80, 1.20
Age	1.53**	1.43, 1.64	1.23**	1.14, 1.32	1.60**	1.46, 1.75	0.99	0.77, 1.28
Age quadratic	0.99**	0.99, 0.99	0.99**	0.99, 0.99	0.99**	0.99, 0.99	1.00	0.99, 1.00
Level of education								
No education (ref.)	----	----	----	----	----	----	----	----
Primary	1.68	0.75, 3.78	0.70	0.35, 1.41	3.41*	1.24, 9.33	1.59	0.25, 9.95
Middle secondary school	2.33*	1.03, 5.27	1.16	0.55, 2.45	3.42*	1.30, 9.00	1.69	0.27, 10.63
Advanced secondary or higher	3.16**	1.33, 7.53	1.55	0.65, 3.71	3.98**	1.44, 11.02	1.13	0.16, 8.00
Marital status								
Married/partnered (ref.)	----	----	----	----	----	----	----	----
Separated/divorced/widowed	0.45**	0.27, 0.75	0.41*	0.20, 0.81	0.74	0.46, 1.19	0.77	0.35, 1.71
Never married	1.52*	1.10, 2.11	1.16	0.83, 1.63	1.55**	1.12, 2.13	1.34	0.75, 2.39
Household wealth								
Quintile 1 (lowest, ref.)	----	----	----	----	----	----	----	----
Quintile 2	0.91	0.45, 1.83	1.27	0.70, 2.30	0.73	0.41, 1.30	0.46	0.13, 1.70
Quintile 3	0.82	0.41, 1.66	1.17	0.57, 2.38	0.68	0.41, 1.14	0.45	0.11, 1.77
Quintile 4	0.99	0.47, 2.11	1.29	0.60, 2.79	0.81	0.50, 1.33	0.28	0.07, 1.18
Quintile 5 (highest)	0.68	0.34, 1.34	0.96	0.48, 1.93	0.62	0.36, 1.07	0.32*	0.08, 0.92
N=	3072		3072		3072		1322	

Notes: *p ≤ .05; **p ≤ .01; analysis for obtaining desired method is limited to women currently using contraception.

For peer review only - http://bmjopen.bmj.com/site/about/guidelines.xhtml

Table 4: Weighted logistic regression results for differences in family planning knowledge among military, Kinshasa 2016

	Heard of implants		Heard of injectables		Heard of condoms		Heard of withdrawal		Heard of rhythm	
	Odds	95% CI	Odds	95% CI	Odds	95% CI	Odds	95% CI	Odds	95% CI
**Military**	**0.23****	**0.11, 0.48**	**0.19****	**0.08, 0.44**	**0.23****	**0.12, 0.47**	**0.05****	**0.02, 0.17**	**0.12****	**0.03, 0.44**
Number of births	1.06	0.95, 1.19	1.27**	1.08, 1.50	0.83*	0.72, 0.97	1.09	0.91, 1.32	1.11	0.93, 1.34
Age	1.49**	1.31, 1.70	1.31**	1.20, 1.43	1.47**	1.26, 1.72	1.27**	1.12, 1.45	1.57**	1.39, 1.77
Age squared	0.99**	0.99, 0.99	0.99**	0.99, 0.99	0.99**	0.99, 0.99	0.99**	0.99, 0.99	0.99**	0.99, 0.99
Level of education										
No education (ref.)	----	----	----	----	----	----	----	----	----	----
Primary	3.01**	1.40, 6.45	1.12	0.64, 1.95	0.36	0.07, 1.90	3.14**	1.58, 6.24	2.40**	1.30, 4.41
Middle secondary school	5.51**	2.32, 13.10	3.15**	1.60, 6.22	0.97	0.13, 7.18	7.85**	3.11, 19.85	4.29**	1.97, 9.31
Advanced secondary or higher	10.85**	4.11, 28.62	6.23**	2.34, 16.56	6.57	0.55, 78.84	27.43**	5.77, 130.44	12.80**	3.74, 43.81
Marital status										
Married/partnered (ref.)	----	----	----	----	----	----	----	----	----	----
Separated/divorced/widowed	1.18	0.56, 2.48	0.96	0.39, 2.36	0.97	0.24, 3.87	1.21	0.63, 2.34	1.44	0.57, 3.64
Never married	0.65*	0.46, 0.94	0.89	0.56, 1.41	1.27	0.58, 2.75	0.95	0.68, 1.33	0.63	0.37, 1.08
Household wealth										
Quintile 1 (lowest, ref.)	----	----	----	----	----	----	----	----	----	----
Quintile 2	0.95	0.48, 1.88	0.83	0.45, 1.54	0.75	0.27, 2.07	0.38**	0.18, 0.79	0.41*	0.18, 0.92
Quintile 3	0.79	0.39, 1.62	0.86	0.42, 1.75	0.99	0.38, 2.60	0.45*	0.20, 0.98	0.48	0.21, 1.14
Quintile 4	0.68	0.33, 1.43	0.86	0.44, 1.70	0.65	0.27, 1.53	0.77	0.31, 1.93	0.60	0.26, 1.38
Quintile 5 (highest)	0.72	0.30, 1.71	0.64	0.28, 1.48	1.06	0.37, 3.01	0.60	0.25, 1.47	0.41*	0.19, 0.93
N=	3078		3077		3080		3079		3077	

Notes: *p ≤ 0.05; **p ≤ 0.01.

For peer review only - http://bmjopen.bmj.com/site/about/guidelines.xhtml

Table 5: Weighted logistic regression results for differences in family planning exposure among military, Kinshasa 2016

	Visited health facility		Heard about FP- radio		Read about FP		FP billboard	
	**Odds**	**95% CI**	**Odds**	**95% CI**	**Odds**	**95% CI**	**Odds**	**95% CI**
**Military**	**0.28****	**0.14, 0.55**	**2.81****	**1.34, 5.92**	**7.72****	**3.13, 19.04**	**9.71****	**3.63, 25.20**
Number of births	1.07	1.00, 1.15	0.96	0.90, 1.03	0.86*	0.77, 0.97	0.97	0.90, 1.05
Age	1.11**	1.04, 1.17	1.11*	1.03, 1.20	1.12	1.00, 1.25	1.11**	1.05, 1.18
Age squared	0.99**	0.99, 0.99	1.00	0.99, 1.00	1.00	0.99, 1.00	0.99**	0.99, 1.00
Level of education								
No education (ref.)	----	----	----	----	----	----	----	----
Primary	1.21	0.60, 2.42	3.30**	1.52, 7.18	3.17	0.96, 10.50	1.22	0.60, 2.56
Middle secondary school	1.36	0.67, 2.76	6.00**	2.33, 15.48	6.05**	1.80, 20.34	1.34	0.75, 2.48
Advanced secondary or higher	1.91	0.85, 4.28	13.93**	4.88, 39.74	15.99**	4.26, 60.00	2.18*	1.12, 4.30
Marital status								
Married/partnered (ref.)	----	----	----	----	----	----	----	----
Separated/divorced/widowed	1.05	0.62, 1.76	0.89	0.57, 1.38	1.00	0.55, 1.80	0.94	0.56, 1.66
Never married	0.53**	0.42, 0.68	0.84	0.59, 1.18	0.82	0.55, 1.24	1.03	0.74, 1.42
Household wealth								
Quintile 1 (lowest, ref.)	----	----	----	----	----	----	----	----
Quintile 2	0.52**	0.33, 0.83	1.05	0.55, 2.00	0.81	0.36, 1.85	1.03	0.62, 1.69
Quintile 3	0.55*	0.35, 0.88	0.86	0.43, 1.72	0.58	0.25, 1.36	1.08	0.63, 1.94
Quintile 4	0.48**	0.31, 0.75	0.84	0.43, 1.66	0.47	0.18, 1.20	0.85	0.53, 1.49
Quintile 5 (highest)	0.59*	0.37, 0.95	0.71	0.30, 1.69	0.73	0.24, 2.20	0.91	0.50, 1.77
N=	3079		3072		3042		3072	

Notes: *p ≤ 0.05; **p ≤ 0.01.

For peer review only - http://bmjopen.bmj.com/site/about/guidelines.xhtml

References

[1] C. F. Djoko *et al.*, "High HIV Type 1 Group M pol Diversity and Low Rate of Antiretroviral Resistance Mutations Among the Uniformed Services in Kinshasa, Democratic Republic of the Congo," *AIDS Res. Hum. Retroviruses*, vol. 27, no. 3, pp. 323–329, Mar. 2011.

[2] M. Grillo *et al.*, "Prevention Interventions for People Living with HIV in Military Settings," *Curr. HIV Res.*, vol. 15, no. 2, pp. 90–94, 2017.

[3] S. H. Miles, "HIV in insurgency forces in sub-Saharan Africa—a personal view of policies," *Int. J. Std Aids*, vol. 14, no. 3, pp. 174–178, 2003.

[4] U. A. Nwokoji and A. J. Ajuwon, "Knowledge of AIDS and HIV risk-related sexual behavior among Nigerian naval personnel," *BMC Public Health*, vol. 4, no. 1, p. 24, 2004.

[5] E. A. Tarimo, T. W. Kohi, M. Bakari, and A. Kulane, "A qualitative study of perceived risk for HIV transmission among police officers in Dar es Salaam, Tanzania," *BMC Public Health*, vol. 13, no. 1, p. 785, 2013.

[6] C. C. Nnebue, U. C. Chimah, T. O. Lawoyin, A. L. Ilika, and C. B. Duru, "Original Article: Socio-demographic Determinants of Sexual Risk Behavior among Senior Secondary Schools Students in a Military Barracks in Nigeria," *Am. J. Public Health Res. Am. J. Public Health Res.*, vol. 3, no. 4, pp. 148–156, Jul. 2015.

[7] E. van der Ryst, G. Joubert, F. Steyn, C. Heunis, J. le Roux, and C. Williamson, "HIV/AIDS-related knowledge, attitudes and practices among South African military recruits," *S. Afr. Med. J.*, vol. 91, no. 7, pp. 587–591, 2001.

[8] E. J. Essien, O. Mgbere, E. Monjok, E. Ekong, S. Abughosh, and M. M. Holstad, "Predictors of frequency of condom use and attitudes among sexually active female military personnel in Nigeria," *HIVAIDS Auckl. NZ*, vol. 2, pp. 77–88, Apr. 2010.

[9] R. N. Hiremath, A. K. Yadav, S. Ghodke, and J. Yadav, "Contraceptive use and its determinants amongst armed forces personnel," *Med. J. Armed Forces India*, 2017.

[10] A. G. Abdulrazaq, S. Kabir, N. S. Mohammad, and I. H. Suleiman, "The effect of educational intervention on family planning knowledge, attitudes, and practices among married women in a military barrack in Northern Nigeria," *Afr. J. Reprod. Health*, vol. 18, no. 1, pp. 93–101, 2014.

[11] U. C. Chimah, T. O. Lawoyin, A. L. Ilika, and C. C. Nnebue, "Contraceptive knowledge and practice among senior secondary schools students in military barracks in Nigeria," *Niger. J. Clin. Pract.*, vol. 19, no. 2, pp. 182–188, 2016.

[12] N. A. Hussain, T. M. Akande, G. K. Osagbemi, S. T. Olasupo, K. Y. Salawu, and E. T. Adebayo, "Perception and practice of contraception among male soldiers in Sobi barracks, Ilorin, Nigeria," *Afr. Health Sci.*, vol. 13, no. 2, pp. 415–422, 2013.

[13] B. Dogo, "A Survey of Knowledge, Attitude and Practice of Family Planning among the Military in the Rukuba Cantonment of Kaduna," *J. Environ. Sci.*, vol. 1, no. 2, pp. 124–132, 1998.

[14] S. Ahmed, A. A. Creanga, D. G. Gillespie, and A. O. Tsui, "Economic status, education and empowerment: implications for maternal health service utilization in developing countries," *PloS One*, vol. 5, no. 6, p. e11190, 2010.

[15] A. A. Creanga, D. Gillespie, S. Karklins, and A. O. Tsui, "Low use of contraception among poor women in Africa: an equity issue," *Bull. World Health Organ.*, vol. 89, no. 4, pp. 258–266, 2011.

For peer review only - http://bmjopen.bmj.com/site/about/guidelines.xhtml

[16] K. Wood and R. Jewkes, “Blood blockages and scolding nurses: barriers to adolescent contraceptive use in South Africa,” *Reprod. Health Matters*, vol. 14, no. 27, pp. 109–118, 2006.
[17] J. Hickes Lundquist and H. L. Smith, “Family formation among women in the US military: Evidence from the NLSY,” *J. Marriage Fam.*, vol. 67, no. 1, pp. 1–13, 2005.
[18] A. K. Erickson, D. A. Nelson, J. G. Shaw, P. D. Loftus, L. M. Kurina, and K. A. Shaw, “Long-Acting Reversible Contraceptive Placement Among Active-Duty U.S. Army Servicewomen,” *Obstet. Gynecol.*, vol. 129, no. 5, pp. 800–809, May 2017.
[19] V. Goyal, S. Borrero, and E. B. Schwarz, “Unintended pregnancy and contraception among active-duty servicewomen and veterans,” *Am. J. Obstet. Gynecol.*, vol. 206, no. 6, pp. 463–469, Jun. 2012.
[20] L. A. Harrington, K. A. Shaw, and J. G. Shaw, “Contraception in US servicewomen: emerging knowledge, considerations, and needs,” *Curr. Opin. Obstet. Gynecol.*, Sep. 2017.
[21] K. Holt, K. Grindlay, M. Taskier, and D. Grossman, “Unintended pregnancy and contraceptive use among women in the U.S. military: a systematic literature review,” *Mil. Med.*, vol. 176, no. 9, pp. 1056–1064, Sep. 2011.
[22] L. Enewold, L. A. Brinton, K. A. McGlynn, S. H. Zahm, J. F. Potter, and K. Zhu, “Oral contraceptive use among women in the military and the general US population,” *J. Womens Health*, vol. 19, no. 5, pp. 839–845, 2010.
[23] D. Gehlbach, “Contraceptive needs complications and new directions for research.,” *Womens Health Issues*, vol. 6, no. 6, pp. 355–8, 1996.
[24] B. R. Karney and J. S. Crown, *Families Under Stress: An Assessment of Data, Theory, and Reseach on Marriage and Divorce in the Military*. Rand Corporation, 2007.
[25] United Nations, *World Population Prospects: The 2015 Revision, Volume II: Demographic Profiles*, 2015th ed. United Nations, New York, NY.
[26] Ministère du Plan et Macro International, *Enquête Démographique et de Santé, République Démocratique du Congo 2007*. Calverton, Maryland, U.S.A.: Ministère du Plan et Macro International., 2008.
[27] Ministère du Plan et Suivi de la Mise en oeuvre de la Révolution de la Modernité (MPSMRM), Ministère de la Santé Publique (MSP) et ICF International, *Enquête Démographique et de Santé en République Démocratique du Congo 2013-2014*. Rockville, Maryland, USA: MPSMRM, MSP et ICF International., 2014.
[28] D. Filmer and L. H. Pritchett, “Estimating wealth effects without expenditure data—or tears: an application to educational enrollments in states of India,” *Demography*, vol. 38, no. 1, pp. 115–132, 2001.
[29] P. Anglewicz, J. Corker, and P. Kayembe, “The fertility of internal migrants to Kinshasa,” *Genus*, vol. 1, no. 73, pp. 1–18, 2017.

For peer review only

For peer review only - http://bmjopen.bmj.com/site/about/guidelines.xhtml

Female Questionnaire

Female Questionnaire

NO	QUESTIONS AND FILTERS	CODING CATEGORIES	SKIP
**IDENTIFICATION**			
A	**Are you in the correct household?**<br>**This is the picture of the front of the home taken during the Household Questionnaire.**<br>**IF NOT, RETURN TO INTERVIEW THE CORRECT HOUSEHOLD.**<br>[ODK will display the photo attached to the linked Household Questionnaire]	Yes ....1<br>No......................0	
B	**Your name:**<br>[Interviewer name from **Female Questionnaire**]<br>**Is this your name?**	Yes ....1<br>No......................0	
	**Enter your name below.**<br>*Please record your name*	Interviewer's Name	
C	**Current date and time.** [ODK will display on screen]<br>**Is this date and time correct?**	Yes ....1<br>No......................0	Skip to E if Yes
D	**Record the correct date and time.**	**Day** **Month** **Year**<br>**Hours** **Min** **AM/PM**	
E	**The following information is from the Female Questionnaire. Please review to make sure you are interviewing the correct respondent.**<br>[ODK will display the province, city, commune, and quartier for Kinshasa EAs and the province, district, *aire de santé*, and village for Kongo Central EAs. In addition, the Enumeration Area, Structure Number, and Household Number entered into the Household Questionnaire linked to this Female Questionnaire will be displayed.]<br>**Is the above information correct?**	Yes ....1<br>No......................0	
	**CHECK: You should be attempting to interview [Respondent's Name]. Is that correct?**<br>*If misspelled, select "yes" here and update the name in question "L."*<br>*If this is the wrong person, you have two options:*<br>*(1) exit and ignore changes to this form. Open the correct form.*<br>*Or*<br>*(2) find and interview the person whose name appears above.*	Yes ....1<br>No......................0	
F	**Is the respondent present and available to be interviewed today?**	Yes ....1<br>No......................0	Skip to K if No

For peer review only

For peer review only - http://bmjopen.bmj.com/site/about/guidelines.xhtml

Female Questionnaire

G	**How well acquainted are you with the respondent?**	Very well acquainted........................1<br>Well acquainted ....2<br>Not well acquainted..........................3<br>Not acquainted.................................4	
G2	**Has the respondent participated in a PMA2020 survey before?**	Yes ....1<br>No...0<br>Don't know ....-88<br>No response.................................-99	

**INFORMED CONSENT**<br>*Find the woman between the ages of 15-49 associated with this Female Follow Up Questionnaire. The interview must have auditory privacy. Read the following greeting:*			
Hello. My name is ________________________________ and I am working for the Kinshasa School of Public Health in collaboration with the Ministry of Health. We are conducting a survey in Kinshasa and Kongo Central that asks women about various reproductive health issues. We would very much appreciate your participation in this survey. This information will help us inform the government to better plan health services. The survey usually takes between 15 and 20 minutes to complete. Whatever information you provide will be kept strictly confidential and will not be shown to anyone other than members of our survey team. Participation in this survey is voluntary, and if we should come to any question you don't want to answer, just let me know and I will go on to the next question; or you can stop the interview at any time. However, we hope that you will participate in this survey since your views are important.<br>At this time, do you want to ask me anything about the survey?			
H	Provide a paper copy of the Consent Form to the respondent and explain it. Then, ask:<br>**May I begin the interview now?**	Yes ....1<br>No..0	Skip to K if No
	**Respondent's signature**<br>*Please ask the respondent to sign or check the box in agreement of their participation.*	GATHER SIGNATURE:<br>Check box: ☐	
I	**Interviewer's name:** [Interviewer name from Household Questionnaire]<br>*Mark your name as a witness to the consent process.*	[ ]	
J	**Respondent's name**<br>[ODK will display the Respondent's name from linked Household Roster]<br>*You may correct the spelling here if it is not correct, but you must be interviewing the person whose name appears below.*	[ ]	
**Section 1 – Respondent's Background, Marital Status, HH characteristics**<br>*Now I would like to ask about your background and socioeconomic conditions.*			
0	**In what month and year were you born?**<br>**The age in the household roster is [AGE].**	Month [ ]<br>Year [ ]	
1	**How old were you at your last birthday?**<br>*Must be more than 14. Must agree with FQ0.*	Age [ ]	
2	**What is the highest level of school you attended?**	Never Attended ....0<br>Primary..1<br>Secondary ....2<br>Tertiary……………………………… 3	

For peer review only - http://bmjopen.bmj.com/site/about/guidelines.xhtml

Female Questionnaire

		No response .... -99	
3	**Are you currently married or living together with a man as if married?**<br>*Probe: If no, ask whether the respondent is divorced, separated, or widowed.*	No, never in union ....0<br>Yes, currently married ....1<br>Yes, living with a man ....2<br>Not currently in union:<br>Divorced / separated ....3<br>Not currently in union: Widow ....4<br>No response .... -99	Skip to 8 if No, never in union
4	**Have you been married or lived with a man only once or more than once?**	Only once ....1<br>More than once ....2<br>No response .... -99	Skip to 5b if Only once
5a	**In what month and year did you start living with your FIRST husband / partner?**<br>*Enter Jan 2020 for no response.*	Month<br>Year	
	[If ≤15 years old at marriage date ODK will display:]<br>**CHECK: Based on the response you entered in FQ5a, the respondent was possibly 15 years old or younger at the time of her first marriage. Did you enter FQ5a correctly?**	Yes ....1<br>No 0	
5b	**Now I would like to ask about when you started living with your CURRENT or MOST RECENT husband / partner. In what month and year was that?**<br>*Enter Jan 2020 for no response.*	Month<br>Year	
	[If ≤15 years old at marriage date ODK will display:]<br>**CHECK: Based on the response you entered in FQ5b, the respondent was possibly 15 years old or younger at the time of her first marriage. Did you enter FQ5b correctly?**	Yes ....1<br>No 0	
	**CHECK 3:** Currently married/cohabitating?	Yes ....1<br>No 0	Skip to 8 if No
6	**Does your husband / partner have other wives or does he live with other women as if married?**	Yes ....1<br>No ....0<br>Don't know.................................. -88<br>No response .... -99	
7	**Is your husband / partner living with you now or is he staying elsewhere?**	Living with respondent ....1<br>Staying elsewhere ....2<br>No response .... -99	
**Section 2 – Reproduction, Pregnancy & Fertility Preferences**<br>*Now I would like to ask about all the births you have had during your life.*			
8a	**How many times have you given birth?**<br>*Enter -99 for no response. 0 is a possible answer.*	Number	Skip to 13 if 0
	**Were all of those live births?**<br>*If no, go back and change FQ8 to record only live birth events.*	Yes ....1<br>No 0	Skip to 9 if 8 was 1
8b	**How many sons and daughters have you given birth to and who were born alive?**		

For peer review only - http://bmjopen.bmj.com/site/about/guidelines.xhtml

Female Questionnaire

		Number ☐	
8c	**Have you ever given birth to a boy or girl who was born alive but later died?**<br>*IF NO, PROBE: Any baby who cried or showed signs of life but did not survive?*	Yes ....1<br>No 0	Skip to 8e if No
8d	**How many have died?**<br>*Enter -88 for do not know and -99 for No response.*<br>*Change FQ8c to 'No' if zero deaths.*	Number ☐	
	**READ THIS CHECK OUT LOUD: Just to make sure I have this right: you had a total of ___ birth(s) during your life, resulting in ____ son(s) or daughter(s) born alive.**<br>**Is that correct?**	Yes ....1<br>No 0	If no, go back and probe to correct 8a-c.
8e	**When was your FIRST birth?**<br>*Please record the date of the first live birth. Date should be found by calculating forward or backward from memorable events if needed. Enter Jan 2020 for no response.*	Month ☐<br>Year ☐	
9	**When was your MOST RECENT birth?**<br>*Please record the date of the MOST RECENT live birth. The date should be found by calculating backwards from memorable events if needed.*<br>*Enter Jan 2020 for no response.*	Month ☐<br>Year ☐	Skip to 11 if not in last year and/or Q8 is 1
10	**When did you give birth before the most recent one?**<br>*Please record the date of the birth before the last. The date should be found by calculating backwards from memorable events if needed.*<br>*Enter Jan 2020 for no response.*	Month ☐<br>Year ☐	
11	**Is your last baby / child still alive?**	Yes ....1<br>No ....0<br>Don't know .... -88<br>No response .... -99	Skip to 13 if Yes
12	**When did your last baby / child die?**<br>*Please record the date of the child's death.*<br>*The date should be found by calculating backwards from memorable events if needed.*<br>*Enter Jan 2020 for no response.*	Month ☐<br>Year ☐	
13	**When did your last menstrual period start?**<br>*If you select days, weeks, months or years, you will enter a number for x on the next screen.*<br>*Enter 0 days for today, not 0 weeks/months/years.*	______ days ago<br>______ weeks ago<br>_______ months ago<br>_______ years ago	

DRC - Round 5

For peer review only - http://bmjopen.bmj.com/site/about/guidelines.xhtml

Female Questionnaire

		Menopausal / Hysterectomy............5<br>Before last birth ....6<br>Never menstruated..........................7<br>No response .... -99	
14	**Are you pregnant now?**	Yes ....1<br>No...0<br>Unsure..2<br>No response .... -99	Skip to 16 if No or Unsure
15	**How many months pregnant are you?**<br>**The most recent birth was: [Date of most recent birth]**<br>*Please record the number of completed months. Enter -88 for do not know, -99 for no response.*	Number of months [ ]	
	**CHECK 14:** Currently pregnant?	Yes ....1<br>No...0	16a if no 16b if yes
16a	**Now I have some questions about the future. Would you like to have a/another child or would you prefer not to have any / any more children?**	Have a/another child ....1<br>No more..2<br>Says she can't get pregnant............3<br>Undecided / Don't know .... -88<br>No response .... -99	Skip to 17a if 1 and 18 for all other
16b	**Now I have some questions about the future. After the child you are expecting now, would you like to have another child, or would you prefer not to have any more children?**	Have a/another child ....1<br>No more/prefer no children ....2<br>Says she can't get pregnant............3<br>Undecided / Don't know .... -88<br>No response .... -99	Skip to 17b if 1 and 18 for all other
17a	**How long would you like to wait from now before the birth of a/another child?**<br>*If you select months or years, you will enter a number for x on the next screen.*<br>*Select "Years" if more than 36 months.*	Months [ ]<br>Years [ ]<br>Soon / now ....1<br>Says she can't get pregnant............2<br>Other ....3<br>Don't know.................................. -88<br>No response .... -99	
17b	**After the birth of the child you are expecting now, how long would you like to wait before the birth of another child?**<br>*If you select months or years, you will enter a number for x on the next screen.*<br>*Select "Years" if more than 36 months.*	Months [ ]<br>Years [ ]<br>Soon / now ....1<br>Says she can't get pregnant............2<br>Other ....3<br>Don't know.................................. -88<br>No response .... -99	
	**CHECK 8:** Number of births	Number of births [ ]	Skip to 19 if 0 births and 14:

For peer review only - http://bmjopen.bmj.com/site/about/guidelines.xhtml

Female Questionnaire

	**CHECK 14**: Currently pregnant?	Yes………………………………… 1<br>No……………………………….0	No. Skip to 18a if 14: no and 18b if 14: yes
18a	**Now I would like to ask a question about your last birth.**<br>**At the time you became pregnant, did you want to become pregnant then, did you want to wait until later, or did you not want to have any / any more children at all?**	Then ....1<br>Later ....2<br>Not at all ....3<br>No response .... -99	
18b	**Now I would like to ask a question about your current pregnancy.**<br>**At the time you became pregnant, did you want to become pregnant then, did you want to wait until later, or did you not want to have any / any more children at all?**	Then ....1<br>Later ....2<br>Not at all ....3<br>No response .... -99	

Section 3A – Contraception

Now I would like to talk about family planning - the various ways or methods that a couple can use to delay or avoid a pregnancy.

An image will appear on the screen for some methods. If the respondent says that she has not heard of the method or if she hesitates to answer, read the probe aloud and show her the image, if available.

19	**Have you ever heard of female sterilization?**<br>PROBE: Women can have an operation to avoid having any more children.<br>[NO IMAGE]	Yes.. 1<br>No .... 0<br>No response……………………..-99	
19	**Have you ever heard of male sterilization?**<br>PROBE: Men can have an operation to avoid having any more children.<br>[NO IMAGE]	Yes.. 1<br>No .... 0<br>No response……………………..-99	
19	**Have you ever heard of the contraceptive implant?**<br>PROBE: Women can have one or several small rods placed in their upper arm by a doctor or nurse, which can prevent pregnancy for one or more years.<br>[IMAGE OF METHOD WILL APPEAR ON SCREEN]	Yes.. 1<br>No .... 0<br>No response……………………..-99	
19	**Have you ever heard of the IUD?**<br>PROBE: Women can have a loop or coil placed inside them by a doctor or a nurse.<br>[IMAGE OF METHOD WILL APPEAR ON SCREEN]	Yes.. 1<br>No .... 0<br>No response……………………..-99	
19	**Have you ever heard of injectables?**	Yes.. 1<br>No 0	

For peer review only - http://bmjopen.bmj.com/site/about/guidelines.xhtml

Female Questionnaire

	PROBE: Women can have an injection by a health provider that stops them from becoming pregnant for one or more months.<br><br>[IMAGE OF SAYANA PRESS AND DEPO PROVERA WILL APPEAR ON SCREEN]	No response……………………..-99	
19	**Have you ever heard of the (birth control) pill?**<br><br>PROBE: Women can take a pill every day to avoid becoming pregnant.<br><br>[IMAGE OF METHOD WILL APPEAR ON SCREEN]	Yes... 1<br>No .... 0<br>No response……………………..-99	
19	**Have you ever heard of emergency contraception?**<br><br>PROBE: As an emergency measure after unprotected sexual intercourse women can take special pills at any time within five days to prevent pregnancy.<br><br>[NO IMAGE]	Yes... 1<br>No .... 0<br>No response……………………..-99	
19	**Have you ever heard of condoms?**<br><br>PROBE: Men can put a rubber sheath on their penis before sexual intercourse.<br><br>[IMAGE OF METHOD WILL APPEAR ON SCREEN]	Yes... 1<br>No .... 0<br>No response……………………..-99	
19	**Have you ever heard of female condoms?**<br><br>PROBE: Women can put a sheath in their vagina before sexual intercourse.<br><br>[IMAGE OF METHOD WILL APPEAR ON SCREEN]	Yes... 1<br>No .... 0<br>No response……………………..-99	
19	**Have you ever heard of the diaphragm?**<br><br>PROBE: Women can place a thin flexible disk in their vagina before sexual intercourse.<br><br>[IMAGE OF METHOD WILL APPEAR ON SCREEN]	Yes... 1<br>No .... 0<br>No response……………………..-99	
19	**Have you ever heard of foam or jelly as a contraceptive method?**<br><br>PROBE: Women can place a suppository, jelly, or cream in their vagina before sexual intercourse to prevent pregnancy.	Yes... 1<br>No .... 0<br>No response……………………..-99	

For peer review only - http://bmjopen.bmj.com/site/about/guidelines.xhtml

Female Questionnaire

	[IMAGE OF METHOD WILL APPEAR ON SCREEN]		
19	**Have you ever heard of the standard days method or Cycle Beads?**<br><br>PROBE: A Woman can use a string of colored beads to know the days she can get pregnant. On the days she can get pregnant, she and her partner use a condom or do not have sexual intercourse.<br><br>[IMAGE OF METHOD WILL APPEAR ON SCREEN]	Yes....... 1<br>No .... 0<br>No response…….-99	
19	**Have you ever heard of the Lactational Amenorrhea Method or LAM?**<br><br>[NO DESCRIPTION; NO IMAGE]	Yes....... 1<br>No .... 0<br>No response…….-99	
19	**Have you ever heard of the rhythm method?**<br><br>PROBE: Women can avoid pregnancy by not having sexual intercourse on the days of the month they think they can get pregnant.<br><br>[NO IMAGE]	Yes....... 1<br>No .... 0<br>No response…….-99	
19	**Have you ever heard of the withdrawal method?**<br><br>PROBE: Men can be careful and pull out before climax.<br><br>[NO IMAGE]	Yes....... 1<br>No .... 0<br>No response…….-99	
19	**Have you ever heard of any other ways or methods that women or men can use to avoid pregnancy?**	Yes....... 1<br>No .... 0<br>No response…….-99	
	**CHECK 14:** Currently pregnant?	Yes....... 1<br>No 0	Skip to 23 if yes
20	**Are you or your partner currently doing something or using any method to delay or avoid getting pregnant?**	Yes....... 1<br>No 0	Skip to 23 if No

For peer review only - http://bmjopen.bmj.com/site/about/guidelines.xhtml

Female Questionnaire

21	**Which method or methods are you using?**<br>**Probe: Anything else?**<br>*Select all methods mentioned. Be sure to scroll to bottom to see all choices.*	Female sterilization .... 1<br>Male sterilization .... 2<br>Implant .... 3<br>IUD .... 4<br>Injectable .... 5<br>Pill .... 7<br>Emergency Contraception .... 8<br>Male Condom .... 9<br>Female Condom .... 10<br>Diaphragm .... 11<br>Foam/Jelly .... 12<br>Std. Days/Cycle beads .... 13<br>LAM .... 14<br>Rhythm method .... 30<br>Withdrawal .... 31<br>Other traditional method .... 39<br>No response .... -99		Skip based on most effective method only<br><br>If injectable is selected, skip to FQ21a<br><br>If LAM is selected but not injectables, skip to 21b<br><br>If FS or MS was selected without injectables or LAM, skip to 22<br><br>If LAM, FS, MS, and injectables are not selected, skip to 26b
21a	**PROBE:** Was the injection administered via syringe or small needle?<br><br>*Show the image to the respondent.*<br><br>[IMAGES OF BOTH INJECTION SYSTEMS WILL APPEAR ON SCREEN]	Syringe .... 1<br>Small needle (Sayana Press) .... 2<br>Both .... 3<br>No response……………………..-99		Skip to CHECK FQ21
21b	**Are you breastfeeding to delay or avoid becoming pregnant?**	Yes........................ 1<br>No .... 0<br>No Response…………………… -99		
	**CHECK FQ21:** Using Female Sterilization and/or Male Sterilization?	Female Sterilization........................<br>Male Sterilization ....<br>None of the above	Y: 1 / N: 0<br>Y: 1 / N: 0<br>-77	Skip to 26b if -77
22	**Did the provider tell you or your partner that this method was permanent?**	Yes........................ 1<br>No .... 0<br>No response……………………..-99		Skip to 26b
23	**Do you know of a place where you can obtain a method of family planning?**	Yes........................ 1<br>No .... 0<br>No response……………………..-99		
	**CHECK 14:** Currently pregnant?	Yes........................ 1<br>No 0		Skip to 24b if yes
24a	**You said that you are not currently using a contraceptive method. Do you think you will use a contraceptive method to delay or avoid getting pregnant at any time in the future?**	Yes........................ 1<br>No .... 0<br>No response……………………..-99		

For peer review only - http://bmjopen.bmj.com/site/about/guidelines.xhtml

For peer review only

Female Questionnaire

24b	**Do you think you will use a contraceptive method to delay or avoid getting pregnant at any time in the future?**	Yes.. 1<br>No .... 0<br>No response……………………..-99	
25	**In the last 12 months, have you ever done something or used a method to delay or avoid getting pregnant?**	Yes.. 1<br>No .... 0<br>No response……………………..-99	Skip to 41 if No
26	**Which method did you use most recently?**<br>**Probe: Anything else?**<br>*Select most effective method (highest method on list). Scroll to bottom to see all choices.*	Implant .... 3<br>IUD .... 4<br>Injectable .... 5<br>Pill .... 7<br>Emergency Contraception .... 8<br>Male Condom .... 9<br>Female Condom .... 10<br>Diaphragm .... 11<br>Foam/Jelly .... 12<br>Std. Days/Cycle beads .... 13<br>LAM .... 14<br>Rhythm method .... 30<br>Withdrawal .... 31<br>Other traditional method .... 39<br>No response .... -99	Skip to FQ26b unless injectables selected
26a	**PROBE:** Was the injection administered via syringe or small needle?<br>*Show the image to the respondent.*<br>[IMAGES OF BOTH INJECTION SYSTEMS WILL APPEAR ON SCREEN]	Syringe .... 1<br>Small needle (Sayana Press).......... 2<br>Both .... 3<br>No response……………………..-99	
26b	**Before you started using [MOST RECENT / CURRENT METHOD], did you talk with your husband / partner about using a contraceptive method?**	Yes.. 1<br>No .... 0<br>Don't know .... -88<br>No response……………………..-99	
27	**When did you begin using your [MOST RECENT / CURRENT METHOD]?**<br>*Calculate backwards from memorable events if needed.*<br>**Age at first use: [Age from FQ20]**<br>**Most Recent Birth: [mm-yyyy]**<br>**Current Marriage: [mm-yyyy]**<br>*Must be at least the ages she started using a contraceptive method (FQ20).*<br>*Must be before today. Respondent must be at least 10 years old.*<br>*Enter Jan 2020 for no response.*	Month [ ]<br>Year [ ]	

For peer review only - http://bmjopen.bmj.com/site/about/guidelines.xhtml

Female Questionnaire

	**CHECK 20:** Currently using contraceptives?	Yes .... 1<br>No 0	Skip to 30 if Yes
28	**When did you stop using your [MOST RECENT METHOD]?**<br>*Please record the date.*<br>*The date should be found by calculating backwards from memorable events if needed. Must be after FQ27.*<br>*Enter Jan 2020 for no response.*	Month<br>Year	
29	**Why did you stop using your (MOST RECENT METHOD)?**	Infrequent sex / husband away.... 1<br>Became pregnant while using .... 2<br>Wanted to become pregnant .... 3<br>Husband / partner disapproved .... 4<br>Wanted more effective method.... 5<br>No method available.... 6<br>Health concerns.... 7<br>Fear of side effects .... 8<br>Lack of access / too far.... 9<br>Costs too much.... 10<br>Inconvenient to use .... 11<br>Fatalistic.... 12<br>Difficult to get pregnant / menopausal .... 13<br>Interferes with body's processes .... 14<br>Other .... 15<br>Don't know .... -88<br>No response .... -99	
30	**You first started using [CURRENT/MOST RECENT METHOD] in [DATE FROM FQ27]. Where did you get it at that time?**<br>*Scroll to bottom to see all choices.*	**PUBLIC SECTOR:**<br>**NATIONAL HOSPITAL....11**<br>**FAMILY PLANNING CLINIC.... 12**<br>**HEALTH CENTRE/POSTE DE SANTE....13**<br>**MATERNITY....14**<br>**COMMUNITY HEALTH VOLUNTEER....15**<br>**REGIONAL HOSPITAL....16**<br><br>**PRIVATE MEDICAL SECTOR:**<br>**PRIVATE HOSPITAL/CLINIC.... 21**<br>**PHARMACY ....22**<br>**NGO....23**<br>**PRIVATE HEALTH CENTER....24**<br>**PRIVATE PRACTICE ....25**<br>**PRIVATE DOCTOR ....26**<br>**MOBILE NURSE....27**<br>**Community health worker (ASC)....28**<br>**Community-based medical student....29**<br><br>**OTHER SOURCE:**<br>**BOUTIQUE....31**<br>**RELIGIOUS INSTITUTION....32**<br>**FRIEND/RELATIVE ....33**<br>**BAR/NIGHT CLUB....34**<br>**LIGABLO/KIOSK....35**<br>**CHAYEUR....36**<br>**OTHER .... 37**<br>**NO RESPONSE.... -99**	

For peer review only

For peer review only - http://bmjopen.bmj.com/site/about/guidelines.xhtml

Female Questionnaire

31	**When you obtained your [MOST RECENT / CURRENT METHOD], were you told by the provider about side effects or problems you might have with a method to delay or avoid getting pregnant?**	Yes.......... 1<br>No .... 0<br>No response .... -99	Skip to 33 if No
32	**Were you told what to do if you experienced side effects or problems?**	Yes.......... 1<br>No .... 0<br>No response .... -99	
33	**At that time, were you told by the family planning provider about methods of family planning other than the [MOST RECENT/CURRENT METHOD] that you could use?**	Yes.......... 1<br>No .... 0<br>No response .... -99	
34	**During that visit, did you obtain the method you wanted to delay or avoid getting pregnant?**	Yes.......... 1<br>No .... 0<br>No response .... -99	Skip to 36 if yes
35	**Why didn't you obtain the method you wanted?**	Method out of stock that day .... 1<br>Method not available at all.......... 2<br>Provider not trained to provide the method.......... 3<br>Provider recommended a different method.......... 4<br>Not eligible for method.......... 5<br>Decided not to adopt a method .... 6<br>Too costly .... 7<br>Other.......... 8<br>No response .... -99	
36	**During that visit, who made the final decision about what method you got?**	You alone.......... 1<br>Provider .... 2<br>Partner .... 3<br>You and provider .... 4<br>You and partner .... 5<br>Other.......... 6<br>No response .... -99	

For peer review only - http://bmjopen.bmj.com/site/about/guidelines.xhtml

Female Questionnaire

	**CHECK 30: You first started using [CURRENT/MOST RECENT METHOD] in [DATE FROM FQ27]. Where did you get it at that time?**	**PUBLIC SECTOR:**<br>**NATIONAL HOSPITAL……11**<br>**FAMILY PLANNING CLINIC…… 12**<br>**HEALTH CENTRE/POSTE DE SANTE……13**<br>**MATERNITY……14**<br>**COMMUNITY HEALTH VOLUNTEER……15**<br>**REGIONAL HOSPITAL……16**<br><br>**PRIVATE MEDICAL SECTOR:**<br>**PRIVATE HOSPITAL/CLINIC…… 21**<br>**PHARMACY ……22**<br>**NGO……23**<br>**PRIVATE HEALTH CENTER……24**<br>**PRIVATE PRACTICE ……25**<br>**PRIVATE DOCTOR ……26**<br>**MOBILE NURSE……27**<br>**Community health worker (ASC) ……28**<br>**Community-based medical student……29**<br><br>**OTHER SOURCE:**<br>**BOUTIQUE……31**<br>**RELIGIOUS INSTITUTION……32**<br>**FRIEND/RELATIVE ……33**<br>**BAR/NIGHT CLUB……34**<br>**LIGABLO/KIOSK……35**<br>**CHAYEUR……36**<br>**OTHER …… 37**<br>**NO RESPONSE…… -99**	Skip to 41 if 30 is 33 OR 37
37	**Would you return to this provider?**<br><br>**Provider: [Type of Provider from FQ30]**	Yes……1<br>No ……0<br>No response ……-99	
38	**Would you refer your relative or friend to this provider / facility?**	Yes……1<br>No ……0<br>No response ……-99	
39	**In the last 12 months, have you paid any fees for family planning services (including the most current method)?**	Yes……1<br>No ……0	Skip to 41 if No
40	**How much did you pay?**<br>*Enter all prices in Congolese Francs. Enter -88 if respondent does not know, -99 for no response.*	Fee [ ]	

For peer review only - http://bmjopen.bmj.com/site/about/guidelines.xhtml

Female Questionnaire

40a	**Where did you obtain [CURRENT METHOD] the last time?**<br><br>***Scroll to bottom to see all choices.***	**PUBLIC SECTOR:**<br>**NATIONAL HOSPITAL……………………………………11**<br>**FAMILY PLANNING CLINIC……………………………. 12**<br>**HEALTH CENTRE/POSTE DE SANTE…………………13**<br>**MATERNITE………………………………………………14**<br>**COMMUNITY HEALTH VOLUNTEER…………………15**<br>**REGIONAL HOSPITAL……………………………………16**<br><br>**PRIVATE MEDICAL SECTOR:**<br>**PRIVATE HOSPITAL/CLINIC……………………………. 21**<br>**PHARMACY ……………………………………………22**<br>**NGO…………………………………………………………23**<br>**PRIVATE HEALTH CENTER………………………………24**<br>**PRIVATE PRACTICE ……………………………………25**<br>**PRIVATE DOCTOR ……………………………………26**<br>**MOBILE NURSE………………………………………27**<br>**FIELDWORKER…………………………………………28**<br>**Community health worker (ASC)……………..…28**<br>**Community-based medical student……………………………………29**<br><br>**OTHER SOURCE:**<br>**BOUTIQUE………………………………………………31**<br>**RELIGIOUS INSTITUTION………………………………32**<br>**FRIEND/RELATIVE ……………………………………33**<br>**BAR/NIGHT CLUB……………………………………34**<br>**LIGABLO/KIOSK…………………………………………35**<br>**CHAYEUR………………………………………………36**<br>**OTHER ………………………………………………… 37**<br>**NO RESPONSE………………………………………… -99**	
41	**Have you ever done anything or tried in any way to delay or avoid getting pregnant?**	Yes ……………………………………1<br>No………………………………………0<br>No response …………………………. -99	Skip to 43 if No
41b	**How old were you when you first used a method to delay or avoid getting pregnant?**<br><br>**The respondent said she was [age from FQ1] years old at her last birthday.**<br><br>*Enter the age in years.*<br>*Enter -88 if respondent does not know.*<br>*Enter -99 if there is no response.*<br>*Cannot be younger than 9.*	Age	
41c	**How many living children did you have at that time, if any?**<br><br>**Note: the respondent said that she gave birth [number of live births] times in FQ8.**<br><br>*Enter -99 for no response*	Number	

For peer review only - http://bmjopen.bmj.com/site/about/guidelines.xhtml

Female Questionnaire

42	**Which method did you first use to delay or avoid getting pregnant?**<br>*Do not read the method choices. Be sure to scroll to bottom to see all choices.*	Female sterilization .... 1<br>Male sterilization .... 2<br>Implant .... 3<br>IUD .... 4<br>Injectable .... 5<br>Pill .... 7<br>Emergency Contraception .... 8<br>Male Condom .... 9<br>Female Condom .... 10<br>Diaphragm .... 11<br>Foam/Jelly .... 12<br>Std. Days/Cycle beads .... 13<br>LAM .... 14<br>Rhythm method .... 30<br>Withdrawal .... 31<br>Other traditional method............. 39<br>No response .... -99	If Injectables is selected, go to 42a
42a	**PROBE:** Was the injection administered via syringe or small needle?<br>*Show the image to the respondent.*<br>[IMAGES OF BOTH INJECTION SYSTEMS WILL APPEAR ON SCREEN]	Syringe .... 1<br>Small needle (Sayana Press) .... 2<br>Both .... 3<br>No response……………………..-99	
	**CHECK 16:** Desire for future child?<br><br>**CHECK 17**: 2 or more years before next child?<br><br>**CHECK 20:** Currently using contraceptive method?	Have a/another child ....1<br>No more/none.................................2<br>Says she can't get pregnant............3<br>Undecided / Don't know .... -88<br><br>No more/none.................................1<br>Less than 2 years ....2<br>2 or more years ....3<br><br>Yes, using contraceptive ....1<br>No, not using contraceptive.............0	Ask 43 to non-users (current or ever) who do not want a/another child or not before 2 years.
43	**You said that you do not want any / anymore children and that you are not using a method to avoid pregnancy.**<br>**Can you tell me the reason why you are not using a method to prevent pregnancy?**<br>**PROBE: Any other reason?**<br>*RECORD ALL REASONS MENTIONED.*<br>*Cannot select "Do Not Know" or "No response" with other options.*<br>*Cannot select "Not married" if FQ3 is "Yes, currently married".*<br>*Scroll to the bottom to see all choices.*	Not married.......................................1<br>Infrequent sex / husband away ....2<br>Menopausal/Hysterectomy...............3<br>Subfecund / infecund ....4<br>Not menstruated since last birth.......5<br>Breastfeeding ....6<br>Husband away for many days..........7<br>Up to God / fatalistic........................8<br>Respondent opposed ....9<br>Husband / partner opposed............10<br>Others opposed..............................11<br>Religious prohibition.......................12<br>Knows no method ....13<br>Knows no source............................14<br>Fear of side effects.........................15<br>Health concerns ....16<br>Lack of access / too far ....17<br>Costs too much18Preferred method n<br>No method available ....20<br>Inconvenient to use 21	

For peer review only - http://bmjopen.bmj.com/site/about/guidelines.xhtml

Female Questionnaire

		Interferes with body's processes....22<br>Takes too much time away from regular duties / too busy to go get one.…………..23<br>Intention to use one but did not get a chance to go to the clinic yet………24<br>Mother-in-law opposed..................25<br>Other ....36<br>Don't know.................................... -88<br>No response .... -99	
44	**In the last 12 months, were you visited by a community health worker who talked to you about family planning?**	Yes ....1<br>No...0<br>No response .... -99	
44a	**In the last 12 months, did you participate in a group talk at the community level about family planning?**	Yes... 1<br>No .... 0<br>No response ....-99	
45	**In the last 12 months, have you visited a health facility for care for yourself or your children?**<br>*For any health services*	Yes... 1<br>No .... 0<br>No response ....-99	Skip to 47 if no
46	**Did any staff member at the health facility speak to you about family planning methods?**	Yes... 1<br>No .... 0<br>No response ....-99	
47	**In the last few months have you:**<br>**Heard about family planning on the radio?**<br>**Seen anything about family planning on the television?**<br>**Read about family planning in a newspaper or magazine?**	Yes / No / NR<br>1 / 0 / -99<br>1 / 0 / -99<br>1 / 0 / -99	
47b	**Have you seen this image before?**<br>***Show the logo to the respondent.***	Yes... 1<br>No .... 0<br>Don't know/Don't recall.................-88<br>No response ....-99	Skip to 47d if 0, -88, or -99
47b 1	**Where did you see this image?**<br>**PROBE: Anywhere else?**<br>*Select all that apply.*	In a health center .... 1<br>In the street .... 2<br>In a pharmacy .... 3<br>On a billboard ……………………….4<br>On the TV ……………………………5<br>On a leaflet …………………………6<br>On a community heath agent's vest…7	

For peer review only - http://bmjopen.bmj.com/site/about/guidelines.xhtml

		Other .... 8<br>Don't know/Don't recall .... -88<br>No response .... -99	
47c	**What is this image trying to say?**<br><br>***Select all that apply.***	Family planning............................... 1<br>Birth spacing .... 2<br>Reproductive health........................ 3<br>The family unit .... 4<br>Other .... 5<br>Don't know .... -88<br>No response .... -99	
47d	**Have you seen the billboard that states "How many children do you want?", and shows the FP logo ?**<br><br>**Show a photo of the billboard to the respondent.**<br>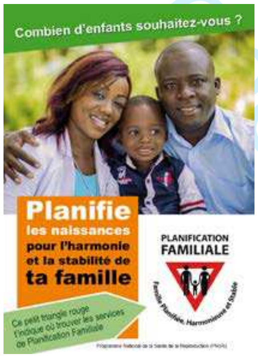	Yes.. 1<br>No .... 0<br>Don't know/Don't recall .... -88<br>No response .... -99	Skip to 48 if 0, -88 or -99
47e	**What is this image trying to say?**<br><br>***Select all that apply.***	Family planning............................... 1<br>Birth spacing .... 2<br>Reproductive health........................ 3<br>The family unit .... 4<br>Other .... 5<br>Don't know / Don't recall .... -88<br>No response .... -99	
	**CHECK FOR THE PRESENCE OF OTHERS. BEFORE CONTINUING, MAKE EVERY EFFORT TO ENSURE PRIVACY.**<br>***Verbally prepare the respondent for sexual activity questions.***		
48	**How old were you when you first had sexual intercourse?**<br><br>***The respondent said she was [age from FQ1] years old at her last birthday.***<br><br>***[She has had x live births.]***<br><br>*Enter the age in years.*<br><br>*Enter -77 if she never had sex.*<br>*Enter -88 if respondent does not know.*<br>*Enter -99 for no response.*	Age	Skip to MM_1 if -77

For peer review only

For peer review only - http://bmjopen.bmj.com/site/about/guidelines.xhtml

Female Questionnaire

	[If age at first sex <10 years:]<br>**You have entered that the respondent was X years old when she first had sexual intercourse. Is this what she said?**<br>*Go back and correct FQ48 if it is not correct.*	Yes ....1<br>No..0	
49	**When was the last time you had sexual intercourse?**<br>*If less than 12 months ago, answer must be recorded in months, weeks, or days.*<br>*Enter 0 days for today.*<br>*You will enter a number for X on the next screen.*	_______ days ago<br>_______ weeks ago<br>________ months ago<br>________ years ago	

For peer review only - http://bmjopen.bmj.com/site/about/guidelines.xhtml
For peer review only

Section 4 – Exposure to Mass Media			
Now I would like to talk in more detail about your exposure to family planning information through mass media.			
MM_1	**Did you watch the television show 'Libala Ya Bosembo' within the past 6 months?**	Yes ....1<br>No ....0<br>No response .... -99	Skip to MM_6 if 0 or -99
MM_2	At the end of each libala ya bosembo show a message is given. The last time you watched the show, what messages had the greatest impression on you? ?<br>**PROBE: What else?**<br>***Select all that apply.***	Couple communication....1<br>Harmony in the family ....2<br>Family planning methods ....3<br>Antenatal care ....4<br>Care for mother and child's health in the postpartum period ....5<br>Amenorrheic period after pregnancy 6<br>Birth spacing ....7<br>Breast feeding ....8<br>Other ....9<br>Don't know / Don't recall .... -88<br>No response .... -99	
MM_3	**Did they talk about using family planning methods during the show?**	Yes ....1<br>No ....0<br>Don't know / Don't recall .... -88<br>No response .... -99	Skip to MM_6 if 0, -88 or -99
MM_4	**Did you speak with someone about the family planning messages from the television show 'Libala Ya Bosembo?'**	Yes ....1<br>No ....0<br>Don't know / Don't recall .... -88<br>No response .... -99	Skip to MM_6 if 0, -88 or -99
MM_5	**With whom did you speak?**<br>**PROBE: Anyone else?**<br>***Select all that apply.***	Medical provider ....1<br>Spouse ....2<br>Other relatives ....3<br>Friends/neighbors ....4<br>Pharmacist/pharmacist's aid ....5<br>Outreach worker ....6<br>People at a seminar/community meeting ....7<br>Other ....8<br>Don't know / Don't recall .... -88<br>No response .... -99	
MM_6	**Have you ever watched the mini-television show called "Elengi"?**	Yes ....1<br>No ....0<br>No response .... -99	Skip to MM_8 if 0 or -99
MM_7	**Last time you watched the mini-television show Elengi, what was it about?**<br>**PROBE: What else?**<br>***Select all that apply.***	Couple communication....1<br>Harmony in the family ....2<br>Family planning methods ....3<br>Antenatal care ....4<br>Care for mother and child's health in the postpartum period ....5<br>Amenorrheic period after pregnancy 6<br>Birth spacing ....7<br>Breast feeding 8	

For peer review only - http://bmjopen.bmj.com/site/about/guidelines.xhtml

Female Questionnaire

		Other ....9<br>Don't know / don't recall .... -88<br>No response .... -99	
MM_8	**Have you ever called the telephone hotline '3-2-1'?**	Yes ....1<br>No....................0<br>No response .... -99	Skip to SE_1 if 0 or -99
MM_9	The last time you called the hotline 3-2-1, what did you want information about?<br><br>**PROBE: Anything else?**<br><br>***Select all that apply.***	Family planning service locations ...1<br>Information about family planning methods....................2<br>Side-effects of FP methods ....3<br>Post-abortion care ....4<br>Post intimate partner violence care..5<br>HIV/AIDS ....6<br>Health in general ....7<br>Other ....8<br>Don't know.................... -88<br>No response .... -99	

	**<u>Section 5 – Perceived self-efficacy in contraceptive use</u>**<br><br>*Now I would like to talk about your level of confidence in your ability to access and use family planning methods. If you are not currently married or have a regular partner, try to imagine how you would respond if you were currently in union.*					
	How confident are you that:	*Very Confident*	*Confident*	*Somewhat confident*	*Not very confident*	*Not at all confident*
SE_1	You can start a conversation with your husband/spouse/partner about family planning/birth spacing?	5	4	3	2	1
SE_2	You can convince your husband/spouse/partner to use a method of family planning/birth spacing?	5	4	3	2	1
SE_3	You can go to a site where methods of family planning / birth spacing are available if you decide to use one?	5	4	3	2	1
SE_4	You can get a method of family planning / birth spacing if you decide to use one?	5	4	3	2	1
SE_5	You could use a method of family planning / birth spacing, even if your husband/partner does not want to?	5	4	3	2	1
SE_6	You can use a method of family planning / birth spacing, even if none of your friends or neighbors use one?	5	4	3	2	1
SE_7	You can use a method of family / child spacing planning, even if your religious leader does not think you should use it?	5	4	3	2	1
SE_8	You will continue to use a method of family planning / birth spacing, even if you experience side effects?	5	4	3	2	1

For peer review only - http://bmjopen.bmj.com/site/about/guidelines.xhtml

Female Questionnaire

**Section 6 – Diarrheal Disease Among Children**<br>*Now I would like to ask about your water practices.*			
50	**How many children under age 5, if any, live in this household for which you are the primary caregiver?**	Number ☐	
	Starting with the youngest child, I'd like to ask you some questions.<br><br>**[ODK Will repeat the FQ51-FQ53 each child under age 5.]**		
51	In what month and year was this child born?<br>**ENTER JAN 2020 FOR NO RESPONSE.**	Month ☐<br>Year ☐	
52	The last time this child passed stools, what was done to dispose of the stools?<br>**FOR ALL FECES, NORMAL OR DIARRHEA.**	Yes	No
	Children use a latrine / toilet........	1	0
	Leave waste where it is........	1	0
	Bury waste in field / yard........	1	0
	Dispose of waste in latrine / toilet........	1	0
	Dispose of waste with rubbish / garbage........	1	0
	Dispose of waste with waste water........	1	0
	Use it as manure........	1	0
	Burn it........	1	0
	No response........	-99	
53	In the past 7 days, has this child had diarrhea?<br><br>***Diarrhea is determined as perceived by mother/ caretaker. If the respondent is not sure what we mean by diarrhea, tell her it means "three or more runny stools per day."***	Yes........ 1<br>No........ 0<br>No Response........ -99	
54	Is it you or your spouse who is military/police personnel?	1) Myself<br>2) Spouse<br>3) Son<br>4) Daughter<br>5) Other (specify): ______	
55	In total how many members of your immediate family are part of the FARDC?	Number____________________________	

DRC - Round 5

For peer review only - http://bmjopen.bmj.com/site/about/guidelines.xhtml

Female Questionnaire

56	What is your/his rank in this service?	**1** Soldier 1st class<br>**2** Soldier 2nd class<br>**3** Corporal<br>**4** Sargent<br>**5** Sargent major<br>**6** 1st Sargent<br>**7** 1st Sargent Adjoint<br>**8** Adjudant de 2eme classe<br>**9** Adjudant<br>**10** Adjudant in chef<br>**11** Second Lieutenant<br>**12** Lieutenant<br>**13** Captain<br>**14** Major<br>**15** Lieutenant Colonel<br>**16** Colonel<br>**17** General Bragadier<br>**18** Lieutenant General<br>**19** General of Army Corps<br>**20** General or Army
57	Have you seen a billboard or banner about family planning that shows a military family?	0 No<br>1 Yes<br>88 Don't know
58	In what province were you born?<br><br>If Kinshasa, skip to question "K"	List provinces
59	For how many years have you lived in Kinshasa?<br><br>0=less than one year	____________Years
**Thank the respondent for her time**<br>*The respondent is finished, but there are still 2 more questions for you to complete outside the home.*		
**LOCATION**		
K	**Location**<br>*Take a GPS point near the entrance to the household. Record location when the accuracy is smaller than 6m.*<br>*GPS coordinates can only be collected when outside.*	Record Location
**QUESTIONNAIRE RESULT**		
L	**How many times have you visited this household to interview this female respondent?**	1st time .... 1<br>2nd time .... 2<br>3rd time 3
M	**Questionnaire result**<br>*Record the result of the Female Questionnaire*	Completed .... 1<br>Not at home .... 2<br>Postponed .... 3<br>Refused .... 4<br>Partly completed .... 5<br>Incapacitated 6

DRC - Round 5

For peer review only

Household Questionnaire

NO	QUESTIONS AND FILTERS	CODING CATEGORIES	SKIP
**IDENTIFICATION**<br>**Please record the following identifying information prior to beginning the interview.**			
A	**Your name: Is this your name?**<br>[ODK will display the name associated with the phone's serial number.]<br>*Check the button next to the name if that is your name and select 'yes' here. Do not check the button if that is not your name and select 'no' here (long press to remove response next to the name if needed).*	Yes ....1<br>No 0	
	**Enter your name below.**<br>*Please record your name*	Interviewer's Name	
B	**Current date and time.**<br>[ODK will display on screen]<br>**Is this date and time correct?**	Yes ....1<br>No 0	Skip to D if Yes
C	**Record the correct date and time**	Date / Month / Day / Year<br>Time / Hour / Minutes / AM/PM	
D1	**Province**	Kinshasa .... 1<br>Kongo Central 2	
D2	**City (Kinshasa) / District (Kongo Central)**	ODK will populate the city of Kinshasa and districts in Kongo Central based on the province selected.	
D3	**Commune (Kinshasa) / Aire de santé (Kongo Central)**	ODK will populate a list of appropriate communes in Kinshasa and *aire de santé* in Kongo Central based on the city or district selected.	If Kinshasa was selected in D1, skip to D6
D4	**Quartier (Kinshasa) / Village (Kongo Central)**	ODK will populate a list of appropriate quartiers in Kinshasa and villages in Kongo Central and chiefdoms based on the commune or *aire de santé* selected.	
D5	**Enumeration area**	ODK will populate a list of appropriate enumeration areas based on the neighborhood in Kinshasa or the village in Kongo Central selected.	If Kongo Central selected in D1, skip to E
D6	**Please note the street or avenue number where the household is located**	*THE SUPERVISOR WILL GIVE YOU THE STREET OR AVENUE NAME.*	
E	**Structure number**<br>*Please record the structure number from the household listing form.*	Number [ ]	

For peer review only - http://bmjopen.bmj.com/site/about/guidelines.xhtml

Household Questionnaire

NO	QUESTIONS AND FILTERS	CODING CATEGORIES	SKIP
F	**Household number**<br>*Please record the household number from the household listing form.*	Number ☐	
	**Check: Have you already sent a form for this structure and household?**<br>*Do not duplicate any form unless you are correcting a mistake in an earlier form.*	Yes ....1<br>No 0	Skip to G if No
	**WARNING: Contact your supervisor before sending this form again.**		
	**CHECK: Why are you resending this form?**<br>*Choose al that apply.*	There are new household members on this form ....1<br>I am correcting a mistake made on a previous form ....2<br>The previous form disappeared from my phone without being sent....3<br>I submitted the previous form and my supervisor told me that is was not received ....4<br>Other reason(s) 5	
G	**Is a member of the household and competent respondent present and available to be interviewed today?**	Yes ....1<br>No 0	Skip to 31 if No
G2	**Did this household participate in a previous PMA2020 survey?**	Yes ....1<br>No ....0<br>Do not know....-88<br>No response ....-99	
**INFORMED CONSENT**<br>**Find a competent member of the household. Read the greeting on the following screen.**			
Hello. My name is ________________________ and I am working for the School of Public Health of Kinshasa in collaboration with the Ministry of Health. We are conducting a survey in Kinshasa and Kongo Central about various health issues in the Democratic Republic of Congo. We would very much appreciate your participation in this survey. This information will help us inform the government to better plan health services. Whatever information you provide will be kept strictly confidential and will not be shown to anyone other than members of our survey team.<br>Participation in this survey is voluntary, and if we should come to any question you don't want to answer, just let me know and I will go on to the next question; or you can stop the interview at any time. However, we hope that you will participate in this survey since your views are important.<br>I am going to ask you questions about your family and other household members. We would then like to ask a different set of questions to female members of this household who are between the ages of 15 and 49 years.<br>At this time, do you want to ask me anything about the survey?			
H	Provide a paper copy of the Consent Form to the respondent and explain it. Then, ask:<br>**May I begin the interview now?**	Yes ....1<br>No 0	Skip to 32 if No
	**Respondent's signature**<br>*Please ask the respondent to sign or check the box in agreement of their participation.*	Gather signature:<br>Check box: ☐	

For peer review only - http://bmjopen.bmj.com/site/about/guidelines.xhtml

Household Questionnaire

NO	QUESTIONS AND FILTERS	CODING CATEGORIES	SKIP
I	**Interviewer's name**<br>*Please record your name as a witness to the consent process. You previously entered "[NAME FROM HQ B]."*		
J	**Respondent's first name.**<br>*Please record the first name of the respondent.*		

For peer review only

For peer review only - http://bmjopen.bmj.com/site/about/guidelines.xhtml

SECTION 1 – Household Roster

I am now going to ask you a series of questions about each usual member of the household or anyone who slept in the house last night.

	1	2	3	4	5	6	7	8
No	First name	Sex	Age (years) If less than one year old, record 0.	Marital Status	Relationship to head of household	Family ID	Is this person a usual member of the household or has he/she slept in the house last night?	Eligible female respondent
		Male .... 1<br>Female 2		Married .... 1<br>Living with a partner .. 2<br>Divorced / separated . 3<br>Widow / widower .... 4<br>Never Married .... 5<br>No response........... -99	Head .... 1<br>Wife/Husband .... 2<br>Son/Daughter .... 3<br>Son/Daughter-in-law . 4<br>Grandchild................ 5<br>Parent .... 6<br>Parent in law .... 7<br>Brother/Sister .... 8<br>Other .... 9<br>Don't know .... -88<br>No response.......... -99		Usual member of the household who slept in the house last night .... 1<br>Usual member of the household who did NOT sleep in the house last night .... 2<br>Visitor who slept in the house last night . 3<br>No response .... -99	Yes ..... 1<br>No....... 0<br><br>ODK will determine and display eligibility
1								
2								
3								
4								
5								

After recording information for one household member, the following prompt is asked to activate a looping script to record information for another member

9	Are there any other usual members of your household or persons who slept in the house last night?	Yes .... 1<br>No 0	
	**READ THIS CHECK OUT LOUD: There are [NUMBER OF HOUSEHOLD MEMBERS ENTERED] household members who are named [NAMES OF ENTERED HOUSEHOLD MEMBERS]. Is this a complete list of the household members?**<br><br>*Remember to include all children in the household.*	Yes .... 1<br>No 0	Skip to 10 if Yes

For peer review only - http://bmjopen.bmj.com/site/about/guidelines.xhtml

Section 2 – Household Characteristics<br>Now I would like to ask you a few questions about the characteristics of your household.					
**NO**	**QUESTIONS AND FILTERS**	**CODING CATEGORIES**			**SKIP**
10	**Please tell me about the items you're your household owns. Does your household have:**<br>*Read out all types and select all that apply. Scroll to bottom to see all choices.*<br>*If an item is reported broken but said to be out of use only temporarily, select the item. Otherwise do not select the item.*		Yes	No	
		Electricity?	1	0	
		A wall clock?	1	0	
		A radio?	1	0	
		A black/white television?	1	0	
		A color television?	1	0	
		A mobile phone?	1	0	
		A landline telephone?	1	0	
		A refrigerator?	1	0	
		A freezer?	1	0	
		A generator/invertor(s)?	1	0	
		A washing machine?	1	0	
		A computer?	1	0	
		A digital photo camera?	1	0	
		A non digital photo camera?	1	0	
		A video deck?	1	0	
		A DVD/CD?	1	0	
		A sewing machine?	1	0	
		A bed?	1	0	
		A table?	1	0	
		A cabinet/cupboard?	1	0	
		A bicycle?	1	0	
		A motorcycle or motor scooter?	1	0	
		A car or truck?	1	0	
		A boat with a motor?	1	0	
		A boat without a motor?	1	0	
		A gas or electric stove?	1	0	
		A chair(s)?	1	0	
		A lamp(s)?	1	0	
		An oven?	1	0	
		A hoe(s)?	1	0	
		Animal-drawn cart?	1	0	
		A canoe or motorized canoe?	1	0	
		A rental house?	1	0	
		None of the above	-77		
		No response	-99		
11a	**Does this household own any livestock, herds, other farm animals, or poultry?**<br>*These livestock can be kept anywhere, not necessarily on the homestead.*	Yes ....1<br>No ....0<br>No response ....-99			Skip to 12a if No or NR

For peer review only - http://bmjopen.bmj.com/site/about/guidelines.xhtml

Household Questionnaire

NO	QUESTIONS AND FILTERS	CODING CATEGORIES	SKIP
11b	**How many of the following animals does this household own?**<br>**Zero is a possible answer. Enter -88 for do not know. Enter -99 for no response.**<br>*The household can keep the livestock anywhere but must own the livestock recorded here.*	Cows or bulls ☐<br>Horses, donkeys, or mules ☐<br>Goats ☐<br>Sheep ☐<br>Hogs/pigs ☐<br>Ducks/canes ☐<br>Hens/cocks/other poultry ☐<br>Other ☐	
12a	**Does this household keep any livestock, herds, other farm animals, or poultry ON THE HOMESTEAD, regardless of who owns these livestock?**<br>*Homestead includes the structure and yard that is close to the structure.*	Yes .... 1<br>No .... 0<br>No response .... -99	Skip to 13 if No or NR
12b	**How many of the following animals does this household keep ON THE HOMESTEAD?**<br>**Zero is a possible answer. Enter -88 for do not know. Enter -99 for no response.**<br>*The household does not need to own the livestock recorded here.*	Cows or bulls ☐<br>Horses, donkeys, or mules ☐<br>Goats ☐<br>Sheep ☐<br>Hogs/pigs ☐<br>Ducks/canes ☐<br>Hens/cocks/other poultry ☐<br>Other ☐	
**<u>Section 3 – Household Observation</u>**<br>**Please observe the floors, roof and exterior walls.**			
13	**Main material of the floor**<br>*Observe.*	NATURAL MATERIAL<br>EARTH / SAND .... 11<br>COW DUNG .... 12<br><br>RUDIMENTARY MATERIAL<br>WOODEN BOARDS .... 21<br>PALM / BAMBOO .... 22<br><br>DEVELOPED MATERIAL<br>PARQUET OR POLISHED WOOD .. 31<br>TAPES VINYL / ASPHALT .... 32<br>TILES .... 33<br>CEMENT .... 34<br>CARPET .... 35<br>OTHER .... 96<br>NO RESPONSE .... -99	

For peer review only - http://bmjopen.bmj.com/site/about/guidelines.xhtml

NO	QUESTIONS AND FILTERS	CODING CATEGORIES	SKIP
14	**Main material of the roof**<br>*Observe.*	NATURAL MATERIAL<br>NO ROOF .... 11<br>THATCH/PALM ROOF .... 12<br>EARTH MOTTES .... 13<br><br>RUDIMENTARY MATERIAL<br>MATS. .... 21<br>PALM / BAMBOO .... 22<br>WOODEN BOARDS .... 23<br>CARDBOARD .... 24<br><br>DEVELOPED MATERIAL<br>SHEET METAL .... 31<br>WOOD .... 32<br>ZINC / FIBER CEMENT .... 33<br>TILE .... 34<br>CEMENT .... 35<br>SHINGLES .... 36<br>OTHER .... 96<br>NO REPONSE .... -99	
15	**Main material of the exterior walls**<br>*Observe.*	NATURAL MATERIAL<br>NO WALL .... 11<br>BAMBOO / CANE / PALM / TRUNK. 12<br>EARTH .... 13<br><br>RUDIMENTARY MATERIAL<br>BAMBOO WITH MUD .... 21<br>STONES WITH MUD .... 22<br>ADOBE NOT COVERED .... 23<br>PLYWOOD .... 24<br>CARDBOARD .... 25<br>RECOVERED WOOD .... 26<br><br>DEVELOPED MATERIAL<br>CEMENT .... 31<br>STONES WITH LIME / CEMENT ... 321<br>BRICKS .... 332<br>CEMENT BLOCKS .... 343<br>COVERED ADOBE .... 354<br>WOOD BOARD / SHINGLES .... 365<br>OTHER .... 96<br>NO RESPONSE .... -99	
**<u>Section 4 – Water, Sanitation and Hygiene</u>**<br>**Now I would like to ask you a few questions about water, sanitation and hygiene.**			
16	**Do you have a place to wash your hands, or do you have a movable container that is not kept in a fixed location, such as a bowl or kettle, that is commonly used for hand washing?**<br><br>*If the container is always in the same location, then count it as a fixed place*	Yes, fixed place .... 1<br>Yes, movable container .... 2<br>No .... 0<br>Don't know .... -88<br>Refuse to answer .... -99	Skip to 19 if No<br><br>Go to 17a if 16 is 1<br><br>Skip to 17b if 16 is 2
17a	**Can you show it to me?**	Yes .... 1<br>No 0	Skip to 19 if No

For peer review only - http://bmjopen.bmj.com/site/about/guidelines.xhtml

Household Questionnaire

NO	QUESTIONS AND FILTERS	CODING CATEGORIES	Yes	No	SKIP
18a	**AT THE PLACE WHERE THE HOUSEHOLD WASHES THEIR HANDS, OBSERVE IF:**	Soap is present ....<br>Water source is present: stored water ....<br>Water source is present: running water ....<br>Hand washing area is near a sanitation facility ....<br>None of the above	1<br>1<br>1<br>1<br>-77	0<br>0<br>0<br>0	
17b	**Can you show me any soap, water, and movable container available in the household used for hand washing?**	Yes .... 1<br>No 0			
18b	**ANYWHERE WITHIN THE HOUSEHOLD, OBSERVE IF:**	Soap is present ....<br>Water source is present: stored water ....<br>Water source is present: running water ....<br>Hand washing container Is observed ....<br>None of the above	1<br>1<br>1<br>-77	0<br>0<br>0	
19	**Which of the following water sources does your household use on a regular basis for any part of the year for any purpose?**<br>*Read out all types and check all that are used. Scroll to the bottom to see all choices.*	Piped Water<br>Piped into dwelling/indoor ....<br>Pipe to yard/plot ....<br>Public tap/standpipe ....<br>Tube well or borehole ....<br>Dug Well<br>Protected Well ....<br>Unprotected Well ....<br>Water from Spring<br>Protected Spring ....<br>Unprotected Spring ....<br>Rainwater........<br>Tanker Truck ....<br>Cart with Small Tank ....<br>Surface water<br>(River / Dam / Lake / Pond / Stream /Canal / Irrigation Channel) ....<br>Bottled Water ....<br>Sachet Water ....<br>No Response	 1<br>1<br>1<br>1<br><br>1<br>1<br><br>1<br>1<br>1<br>1<br>1<br><br>1<br>1<br>1<br>-99	 0<br>0<br>0<br>0<br><br>0<br>0<br><br>0<br>0<br>0<br>0<br>0<br><br>0<br>0<br>0	If only one source is selected, skip to HQ22
20	**What is the main source of drinking water for members of your household?**<br>**Selections from HQ19: [ODK will list water sources selected for HQ19]**<br>*Read out HQ19 selections only.*	Piped Water<br>Piped into dwelling/indoor ....1<br>Pipe to yard/plot ....2<br>Public tap/standpipe ....3<br>Tube well or borehole ....4<br>Dug Well<br>Protected Well ....5<br>Unprotected Well ....6<br>Water from Spring<br>Protected Spring ....7<br>Unprotected Spring ....8<br>Rainwater........ 9<br>Tanker Truck ....10<br>Cart with Small Tank ....11<br>Surface water<br>(River / Dam / Lake / Pond / Stream / Canal / Irrigation Channel) .... 12<br>Bottled Water ....13<br>Sachet Water ....14<br>No Response ....-99			

For peer review only

For peer review only - http://bmjopen.bmj.com/site/about/guidelines.xhtml

Household Questionnaire

NO	QUESTIONS AND FILTERS	CODING CATEGORIES	SKIP
21	**What is the main source of water used by your household for other purposes such as cooking and hand washing?**<br>**Selections from HQ19: [ODK will list water sources selected for HQ19]**<br>*Read out HQ19 selections only.*	Piped Water<br>Piped into dwelling/indoor ....1<br>Pipe to yard/plot ....2<br>Public tap/standpipe ....3<br>Tube well or borehole ....4<br>Dug Well<br>Protected Well ....5<br>Unprotected Well ....6<br>Water from Spring<br>Protected Spring ....7<br>Unprotected Spring ....8<br>Rainwater.................... 9<br>Tanker Truck ....10<br>Cart with Small Tank ....11<br>Surface water<br>(River / Dam / Lake / Pond / Stream / Canal / Irrigation Channel).................... 12<br>Bottled Water ....13<br>Sachet Water ....14<br>No Response ....-99	
	**Questions HQ 22 to HQ 25 will repeat x times, once for each water source selected in HQ 19. These sources include:**<br>**[ODK will display HQ19 selections.]**		
22	**You mentioned that you used [WATER SOURCE]. At any time of the year, does your household use water from this source for:**	Yes / No<br>Drinking .... 1 / 0<br>Cooking .... 1 / 0<br>Livestock .... 1 / 0<br>Gardening / agriculture .... 1 / 0<br>Business venture.................... 1 / 0<br>Washing .... 1 / 0<br>No response.................... -99	
23	**Is [WATER SOURCE] typically available:**<br>*Read all choices out loud.*	All of the year .... 1<br>Some of the year.................... 2<br>Small part of the year .... 3<br>No response.................... -99	
24	**At a time of year when you expect to have water from [WATER SOURCE], is it usually available?**	Yes, always .... 1<br>No, intermittent and predictable .... 2<br>No, intermittent and unpredictable .... 3<br>No response.................... -99	
25	**How long does it take to go to [WATER SOURCE], get water, and come back?**<br>*Zero is a possible answer*<br>*Enter -88 for do not know*<br>*Enter -99 for no response*<br>*Convert time into minutes.*<br>*Answer includes waiting time in line.*	Minutes: [ ]	
26	**Does your household have a garden?**<br>*A garden is a place to grow vegetables.*	Yes .... 1<br>No.................... 0<br>No response.................... -99	

For peer review only - http://bmjopen.bmj.com/site/about/guidelines.xhtml

Household Questionnaire

NO	QUESTIONS AND FILTERS	CODING CATEGORIES	SKIP
27	**Do members of your household use any of the following toilet facilities?**<br>*Read out all types and check all that are used. Scroll to the bottom to see all choices.*	Yes / No<br>Flush/pour flush toilets connected to:<br>Piped sewer system .... 1 0<br>Septic tank .... 1 0<br>Pit Latrine .... 1 0<br>Elsewhere .... 1 0<br>Unknown / Not sure / Don't know .... 1 0<br>Ventilated improved pit latrine .... 1 0<br>Pit latrine with slab .... 1 0<br>Pit latrine without slab .... 1 0<br>Bucket toilet .... 1 0<br>Composting toilet .... 1 0<br>Hanging toilet /Hanging latrine .... 1 0<br>No facility / bush / field .... 1 0<br>Other: .... 1 0<br>No Response .... -99	
28	**What is the main toilet facility used by members of your household?**<br>**HQ27: [ODK will display HQ27 selections]**<br>*The main facility must be selected in HQ 27.*	Flush/pour flush toilets connected to:<br>Piped sewer system .... 1<br>Septic tank .... 2<br>Pit Latrine .... 13<br>Elsewhere .... 3<br>Unknown / Not sure / Don't know ..... 4<br>Ventilated improved pit latrine .... 5<br>Pit latrine with slab .... 6<br>Pit latrine without slab/open pit .... 7<br>Bucket toilet .... 8<br>Composting toilet .... 9<br>Hanging toilet /Hanging latrine .... 10<br>No facility / bush / field .... 11<br>Other: .... 12<br>No Response .... -99	
	**Question HQ 29 will repeat x times, once for each sanitation facility selected in HQ27. These facilities include:**<br>**HQ27: [ODK will display HQ27 selections]**		
29	**How often does your household typically use: [TOILET FACILITY TYPE]?**<br>*Regular practices at the household only.*	Always .... 1<br>Most of the time .... 2<br>Occasionally .... 3<br>Rarely .... 4<br>No response .... -99	
29b	**Do you share this toilet facility with other households or the public?**	Not shared .... 1<br>Shared with less than ten households .... 2<br>Shared with ten or more households .... 3<br>Shared with the public. .... 4<br>No response .... -99	Skip to HQ30 if not 2

For peer review only

For peer review only - http://bmjopen.bmj.com/site/about/guidelines.xhtml

Household Questionnaire

NO	QUESTIONS AND FILTERS	CODING CATEGORIES	SKIP
29c	**Enter the number of households that share this facility (including your own).**<br>**[TOILET FACILITY TYPE]**<br>*Must be between 2 and 9.*<br>*If 10 or greater, swipe back to HQ29b and choose "shared with ten or more households."*<br>*Enter -99 for no response.*	Number of Households: ☐	
30	**How many people within your household regularly use the bush / field at home or at work?**<br>**There are x people in this household. Enter -88 for do not know, -99 for no response.**	Number of People: ☐	
31	**Ask permission to take a photo of the entrance of the house.**<br>**Did you get consent to take the photo?**	Yes .... 1<br>No 0	Skip L if No
**Thank the respondent for her/his time.**<br>*The respondent is finished, but there is still more for you to complete outside the home.*			
**LOCATION AND QUESTIONNAIRE RESULT**			
K	**Location**<br>**Take a GPS point near the entrance to the household. Record location when the accuracy is smaller than 6m.**	RECORD LOCATION	
	**CHECK 32:** Permission to take photo?		Skip to M if No
L	**Ensure that no people are in the photo**	TAKE PICTURE<br>CHOOSE IMAGE	
M	**How many times have you visited this household?**	1st time .... 1<br>2nd time .... 2<br>3rd time 3	
N	**Questionnaire result**<br>*Record the result of the Household Questionnaire*	Completed .... 1<br>No household member at home or no competent respondent at home at time of visit .... 2<br>Postponed .... 3<br>Refused .... 4<br>Partly completed .... 5<br>Dwelling vacant or address not a dwelling .... 6<br>Dwelling destroyed .... 7<br>Dwelling not found .... 8<br>Entire household absent for extended period of time 9	

For peer review only - http://bmjopen.bmj.com/site/about/guidelines.xhtml

STROBE Statement—Checklist of items that should be included in reports of ***cross-sectional studies***

	Item No	Pg #	Recommendation
**Title and abstract**	1	1-2	(*a*) Indicate the study's design with a commonly used term in the title or the abstract
			(*b*) Provide in the abstract an informative and balanced summary of what was done and what was found
		**Introduction**	
Background/rationale	2	3-4	Explain the scientific background and rationale for the investigation being reported
Objectives	3	4	State specific objectives, including any prespecified hypotheses
		**Methods**	
Study design	4	5-7	Present key elements of study design early in the paper
Setting	5	4	Describe the setting, locations, and relevant dates, including periods of recruitment, exposure, follow-up, and data collection
Participants	6	5	(*a*) Give the eligibility criteria, and the sources and methods of selection of participants
Variables	7	6	Clearly define all outcomes, exposures, predictors, potential confounders, and effect modifiers. Give diagnostic criteria, if applicable
Data sources/ measurement	8*	5-6	For each variable of interest, give sources of data and details of methods of assessment (measurement). Describe comparability of assessment methods if there is more than one group
Bias	9	10	Describe any efforts to address potential sources of bias
Study size	10	5-6	Explain how the study size was arrived at
Quantitative variables	11	6	Explain how quantitative variables were handled in the analyses. If applicable, describe which groupings were chosen and why
Statistical methods	12	7	(*a*) Describe all statistical methods, including those used to control for confounding
		7	(*b*) Describe any methods used to examine subgroups and interactions
		n/a	(*c*) Explain how missing data were addressed
		7	(*d*) If applicable, describe analytical methods taking account of sampling strategy
		n/a	(*e*) Describe any sensitivity analyses
		**Results**	
Participants	13*	6	(a) Report numbers of individuals at each stage of study—eg numbers potentially eligible, examined for eligibility, confirmed eligible, included in the study, completing follow-up, and analysed
		n/a	(b) Give reasons for non-participation at each stage
		n/a	(c) Consider use of a flow diagram
Descriptive data	14*	7-8	(a) Give characteristics of study participants (eg demographic, clinical, social) and information on exposures and potential confounders
		n/a	(b) Indicate number of participants with missing data for each variable of interest
Outcome data	15*	7-8	Report numbers of outcome events or summary measures
Main results	16	7-9	(*a*) Give unadjusted estimates and, if applicable, confounder-adjusted estimates and their precision (eg, 95% confidence interval). Make clear which confounders were adjusted for and why they were included
		n/a	(*b*) Report category boundaries when continuous variables were categorized
		n/a	(*c*) If relevant, consider translating estimates of relative risk into absolute risk for a meaningful time period
Other analyses	17	n/a	Report other analyses done—eg analyses of subgroups and interactions, and

For peer review only - http://bmjopen.bmj.com/site/about/guidelines.xhtml

			sensitivity analyses
		**Discussion**	
Key results	18	9-10	Summarise key results with reference to study objectives
Limitations	19	10	Discuss limitations of the study, taking into account sources of potential bias or imprecision. Discuss both direction and magnitude of any potential bias
Interpretation	20	10	Give a cautious overall interpretation of results considering objectives, limitations, multiplicity of analyses, results from similar studies, and other relevant evidence
Generalisability	21	10	Discuss the generalisability (external validity) of the study results
		**Other information**	
Funding	22	10-11	Give the source of funding and the role of the funders for the present study and, if applicable, for the original study on which the present article is based

*Give information separately for exposed and unexposed groups.

Note: An Explanation and Elaboration article discusses each checklist item and gives methodological background and published examples of transparent reporting. The STROBE checklist is best used in conjunction with this article (freely available on the Web sites of PLoS Medicine at http://www.plosmedicine.org/, Annals of Internal Medicine at http://www.annals.org/, and Epidemiology at http://www.epidem.com/). Information on the STROBE Initiative is available at www.strobe-statement.org.

For peer review only
